# Supplementary material for: A virtual library for behavioral performance in standard conditions—rodent spontaneous activity in an open field during repeated testing and after treatment with drugs or brain lesions
Source: Gigascience. 2022 Oct 20;11:giac092. doi: 10.1093/gigascience/giac092 (PMC9581716; doi:10.1093/gigascience/giac092)
Supplement: giac092_GIGA-D-22-00119_Revision_1 [file giac092_giga-d-22-00119_revision_1.pdf]

GigaScience

A Virtual Library for Behavioral Performance in Standard Conditions – Rodent Spontaneous Activity in an Open Field during Repeated Testing and after Treatment with Drugs or Brain Lesions

--Manuscript Draft--

**Manuscript Number:**	GIGA-D-22-00119R1
**Full Title:**	A Virtual Library for Behavioral Performance in Standard Conditions – Rodent Spontaneous Activity in an Open Field during Repeated Testing and after Treatment with Drugs or Brain Lesions
**Article Type:**	Data Note
**Funding Information:**	Institute of Neurosciences, Mental Health and Addiction — Dr Henry Szechtman<br>Ontario Mental Health Foundation — Dr Henry Szechtman<br>Natural Sciences and Engineering Research Council of Canada — Dr Henry Szechtman
**Abstract:**	Background : Beyond their specific experiment, video records of behavior have future value; for example, as inputs for new experiments or for yet unknown types of analysis of behavior— similar to tissue or blood sample banks in life sciences where clinically-derived or otherwise well-described experimental samples are stored to be available for some unknown potential future purpose.<br>Findings : Research using an animal model of obsessive-compulsive disorder employed a standardized paradigm where the behavior of rats in a large open field was video recorded for 55 min on each test. From 43 experiments, there are 19,976 such trials that amount to over 2 years of continuous recording. In addition to videos, there are two video-derived raw data objects: XY locomotion coordinates, and plots of animal trajectory. To motivate future use, the three raw data objects are annotated with a general schema—one that abstracts the data records from their particular experiment while providing, at the same time a detailed list of independent variables bearing on behavioral performance. The raw data objects are deposited as 43 datasets but constitute, functionally, a library containing one large dataset.<br>Conclusions : Size and annotation schema give the library high re-use potential: in applications using machine learning techniques, statistical evaluation of subtle factors, simulation of new experiments, or as educational resource. Ultimately, the library can serve both as the seed and the test bed to create a machine searchable virtual library of linked open datasets for behavioral performance in defined conditions.
**Corresponding Author:**	Henry Szechtman, Ph.D.<br>McMaster University Department of Psychiatry and Behavioural Neurosciences<br>Hamilton, CANADA
**Corresponding Author Secondary Information:**	
**Corresponding Author's Institution:**	McMaster University Department of Psychiatry and Behavioural Neurosciences
**Corresponding Author's Secondary Institution:**	
**First Author:**	Henry Szechtman, Ph.D.
**First Author Secondary Information:**	
**Order of Authors:**	Henry Szechtman, Ph.D.<br>Anna Dvorkin-Gheva<br>Alex Gomez-Marin
**Order of Authors Secondary Information:**	
**Response to Reviewers:**	Dear Dr Nogoy<br>We thank you and the two reviewers for their positive and helpful comments on our

Powered by Editorial Manager® and ProduXion Manager® from Aries Systems Corporation

manuscript. Below we respond to the reviewers' comments and indicate how we revised the manuscript.

Reviewer 1

Reviewer was overall positive about the submission but had 4 areas of concern:

1.Reviewer did not believe that the Introduction was sufficiently comprehensive in putting our database into the context of other available datasets. Furthermore, we believe the Reviewer was concerned that our lack of comprehensiveness could be interpreted by readers that video datasets of behavior do not exist and ours is the first one, or completely unique.

a.We apologize if the Introduction gave the impression that ours is the only video dataset of behavior. We certainly did not intend to suggest this. The references cited in the first paragraph of the Introduction point to the breadth of video analysis tools and explicitly or by implication to video datasets. In the revised manuscript, we added several more references to the list of video analysis tools/datasets that we became aware of, following the Reviewer's critique. We did not add a citation to OpenMonkeyStudio as this tool (using 64 cameras) seems to be in a league way above our video recordings.

2.Reviewer suggests we should be "more specific about impacts and intended use cases", by describing in greater detail the type of analyses that should be performed on the dataset.

a.We added to the section "Re-use Potential" (p. 25) a few more examples of the type of questions that could be explored using the deposited digital library. However, we do not go into detail because the purpose of the submitted Data Note is quite focused—describe in detail the properties of a database that has re-use potential. Unlike a GigaScience Research Report, a Data Note is not intended to demonstrate novel findings obtained by some re-analysis of the database.

3.Reviewer suggests that "sample data analysis scripts would elevate the manuscript's impact".

a.We considered following this suggestion and including the Mathematica scripts we employed in our workflow. However, because of dependencies of those scripts on the SEE Workshop, the task became onerous since the Mathematica scripts would need modifications and this would be a project in itself. Similarly, the Reviewer's suggestion that we provide "some simple analysis scripts" in python, is not something that we have available or can do for inclusion with this Data Note.

4.Reviewer suggests that the readability of the paper would be enhanced by converting some of the tables to figures, e.g., Table 4 to a series of bar graphs.

a.We looked whether the reader would be served better by converting the tables (including Table 4) to a series of graphs. We do not believe so. Tables provide lots of detail information, which is not easily visible with graphs; graphs are best for spotting trends with a glance, not for conveying details.

Reviewer 2

We are grateful to the Reviewer for the detailed summary of the manuscript content. Grateful—because the summary makes clear that the manuscript achieved its intended goal of describing the properties of a database that has re-use potential. Nevertheless, the Reviewer stated several specific concerns, to which we responded in the revised manuscript as follows:

I.Reviewer highlights that our "annotation schema is well constructed" and could be taken as an example to organize "own data". Thank you for this feedback!

II.In points ii.and iii, Reviewer comments that the manuscript does not mention limitations on re-use potential such as image resolution and effective size. Moreover, the Reviewer indicates that there is no clear information as to the effective size of available data objects meeting more than two conditions.

a.On pages 25-26 we added a section called Limitations on Re-use Potential where we address the points raised by the Reviewer, including how to obtain the count of available data objects meeting multiple criteria. We trust that this section is satisfactory.

b.Although in the manuscript we did not elaborate on it, it seems that in terms of video resolution the issue is not the dimensions of the image per se but rather how much of the image is occupied by the object of interest. For example, in the paper on whisker analysis cited in subsection Video resolution, video resolution is also 352x240 pixels but almost the entire head of the mouse occupies the video frame.

III.This comment is a continuation of point ii addressed above.

Powered by Editorial Manager® and ProduXion Manager® from Aries Systems Corporation

	IV.As we understand it, Reviewer seems to suggest that because activity in the open field is affected by environmental structure this limits the re-use potential of the database to those interested in OCD.<br>a.We don't fully understand the above comment and the implication of database usefulness limited OCD research. Spontaneous behavior in general is subject to environmental constraints, and the empirical question is to what degree this is the case in specified situations, a question that could be addressed using the library since the conditions are well specified. Moreover, not all treatments yield OCD behavior; e.g., saline injections. We did not respond to point iv in the revision of the manuscript.<br>V.Reviewer indicates that the Data Note lacks "scientific novelty" but this would be remedied if the manuscript included results from some analysis using the library to address "new questions".<br>a.As noted in the response to Reviewer 1 (point 2) we added a few more examples of interesting questions that could be addressed in re-using the database. We do not provide results from any potential analysis since this is not a requirement of a Data Note publication, and was not our intended objective. Rather, our scientific objective was to "describe in detail the properties of a database that has re-use potential", as noted above in response to Reviewer 1.<br>VI.Reviewer notes that "even the database itself is not novel because at least partly is "available in publications of the original studies and in the FRDR".<br>a.On top of page 6, we indicated that much of the pertinent information describing the raw data objects was collated from our published reports, which may have led the Reviewer to infer that together with those publications we deposited the underlying raw data objects. However, this is not the case. Consequently, we appended the following sentence to the end of the paragraph: "Note that those reports were not accompanied with the publication of datasets containing the raw data objects described in the present Data Note paper."<br>b.It is the case that the raw data objects were deposited in FRDR and the submitted Data Note manuscript indicates that those data objects would be mirrored in the GigaDB database. However, the latter is no longer the case (see below), so there is only one deposit of the raw data objects, and that is in the FRDR repository. Importantly, what is new and novel is the Data Note report, which provides in a publication form a detailed exposition of the annotation schema, the associated metadata, the re-use potential of the database, and the suggestion that our library could be used as a seed towards an effort to develop a schema and a platform which would allow the construction of a "machine searchable virtual library of behavioral performance in defined conditions".<br>VII.Reviewer suggests we would make a more significant scientific contribution if we developed our "elegant schema further and transform it into an indexing tool that organizes broader datasets, already available in different repositories".<br>a.Alas, what the Reviewer suggests is correct. When preparing to put our data in a repository I looked for templates how to annotate the raw data objects, but did not find a suitable one. Further inquiries on metadata annotations made it abundantly clear that this is a complex issue, beyond my current skill set, and hence came out with the annotation schema described here. Nevertheless, as stated in the last paragraph of the Re-use Potential section: "The library may have yet another utility, a less obvious one but potentially impactful. As raised in the Introduction, the underlying motivation in making the raw data objects publically available is to ultimately create a machine searchable virtual library of behavioral performance in defined conditions. The approach used here in annotations of the raw data objects is a step towards this goal. However, to achieve the goal of searchable linked open datasets requires a more sophisticated and formal schema [68-70]. The library described here could be of value both as the seed and test bed in this endeavor."<br><br>Other changes in the revised manuscript<br>After the manuscript was submitted, the journal editors re-evaluated their original suggestion to mirror in the GigaScience Database the raw data objects available in the FRDR repository. Instead, it was proposed that the metadata annotations be deposited in GigaDB and include download links to the files in FRDR. Consequently, we changed the pertinent text in the revised manuscript to reflect this state.<br>In addition, the revised manuscript underwent some minor edits, including the addition of a few references.<br><br>We thank you for your efforts to make our report a first rate publication – we truly

	appreciate this. We hope you find this revision suitable and we look forward to hearing from you.<br><br>Sincerely yours,<br>Henry Szechtman, PhD<br>Professor Emeritus
Additional Information:	
**Question**	**Response**
Are you submitting this manuscript to a special series or article collection?	No
**Experimental design and statistics**<br><br>Full details of the experimental design and statistical methods used should be given in the Methods section, as detailed in our Minimum Standards Reporting Checklist. Information essential to interpreting the data presented should be made available in the figure legends.<br><br>Have you included all the information requested in your manuscript?	Yes
**Resources**<br><br>A description of all resources used, including antibodies, cell lines, animals and software tools, with enough information to allow them to be uniquely identified, should be included in the Methods section. Authors are strongly encouraged to cite Research Resource Identifiers (RRIDs) for antibodies, model organisms and tools, where possible.<br><br>Have you included the information requested as detailed in our Minimum Standards Reporting Checklist?	Yes
**Availability of data and materials**<br><br>All datasets and code on which the conclusions of the paper rely must be either included in your submission or deposited in publicly available repositories (where available and ethically	Yes

Powered by Editorial Manager® and ProduXion Manager® from Aries Systems Corporation

appropriate), referencing such data using a unique identifier in the references and in the “Availability of Data and Materials” section of your manuscript.<br><br>Have you have met the above requirement as detailed in our Minimum Standards Reporting Checklist?	

Powered by Editorial Manager® and ProduXion Manager® from Aries Systems Corporation

Manuscript

Click here to access/download;Manuscript;GigaScience_ms_20220510_revisi

A Virtual Library for Behavioral Performance in Standard Conditions – Rodent Spontaneous Activity in an Open Field during Repeated Testing and after Treatment with Drugs or Brain Lesions

Henry Szechtman[1§], **Anna Dvorkin-Gheva**[2] and **Alex Gomez-Marin**[3]

[1]Department of Psychiatry and Behavioural Neurosciences
[2]Department of Pathology & Molecular Medicine
McMaster University, Hamilton, Ontario, Canada
and
[3]Instituto de Neurociencias (CSIC-UMH), Alicante, Spain.

Running Title: Library of behavioral performance data objects

Figures: 5; Tables: 7; Abstract: 243 words

§Correspondence:
Dr. H Szechtman
Department of Psychiatry and Behavioural Neurosciences
McMaster University
1280 Main Street West
Health Science Centre, Room 4N82
Hamilton, Ontario
CANADA L8S 4K1
Email: szechtma@mcmaster.ca

ABSTRACT

Background: Beyond their specific experiment, video records of behavior have future value; for example, as inputs for new experiments or for yet unknown types of analysis of behavior—similar to tissue or blood sample banks in life sciences where clinically-derived or otherwise well-described experimental samples are stored to be available for some unknown potential future purpose.

Findings: Research using an animal model of obsessive-compulsive disorder employed a standardized paradigm where the behavior of rats in a large open field was video recorded for 55 min on each test. From 43 experiments, there are 19,976 such trials that amount to over 2 years of continuous recording. In addition to videos, there are two video-derived raw data objects: XY locomotion coordinates, and plots of animal trajectory. To motivate future use, the three raw data objects are annotated with a general schema—one that abstracts the data records from their particular experiment while providing, at the same time a detailed list of independent variables bearing on behavioral performance. The raw data objects are deposited as 43 datasets but constitute, functionally, a library containing one large dataset.

Conclusions: Size and annotation schema give the library high re-use potential: in applications using machine learning techniques, statistical evaluation of subtle factors, simulation of new experiments, or as educational resource. Ultimately, the library can serve both as the seed and the test bed to create a machine searchable virtual library of linked open datasets for behavioral performance in defined conditions.

KEYWORDS

animal model of obsessive compulsive disorder
open field
exploration
chronic drug treatments
brain lesion treatments
behavioral sensitization
video recording
repeated testing
male rats Long-Evans
large dataset

> *If meaningful correlations are to be made between brain mechanisms and behavior, then the analysis of behavior will require as much sophistication and attention to detail as the analysis of the brain [1]; see also, [2, 3].*

INTRODUCTION

Technological innovations and methodological advances make possible increasingly sophisticated and detailed analysis of behavior [4-6]. A pertinent example here is videography [7, 8]. Video playback at various speeds, like a microscope at higher powers of magnification, allows for ever more detailed analysis of motor activity, whether through manual or semi-automatic scoring [9-11] or by the application of automated computer software systems [12-22]. Furthermore, the collected videos form a permanent record, useful for re-checking of experimental findings.

Video records, by capturing the entire richness of behavior, have potential utility well beyond their immediate value in a particular experiment. They could be reused as inputs for new experiments to test different hypotheses. They can be reused to analyze aspects of behavior not of interest in the particular experiment. Importantly, they could serve as data for new types of analysis of behavior, not yet available. This is not unlike the banking of tissues or blood samples in life sciences where clinically-derived or otherwise well-described experimental samples are stored to be available for some unknown potential future purpose [23, 24].

Videos have this potential utility if annotated properly. Clearly, there is little potential utility if annotations have meaning limited to the specific experiment. Instead, the videos must be annotated with a more general schema—one that abstracts the records from their particular experiment while providing, at the same time a detailed list of independent variables bearing on behavioral performance. Ultimately, such a well-developed schema can serve as a platform to implement machine searchable linked open datasets, forming thereby a virtual library of behavioral performance.

Note that this conceptualization differs from the typical objectives motivating the deposit of raw and processed data in a public repository. The usual motivation for making a dataset available is to provide the opportunity for other scientists to assess whether the experimental data and analysis warrant the conclusions reached in the published report. In contrast, the motivation proposed here is to provide raw data that can be of value outside the given experiment, for yet unknown analysis or potential use.

Here we describe a library of video records of rodent behavior collected from 43 experiments during 15 years of research employing an animal model of obsessive compulsive disorder (OCD)

[25]. Use of this model involved testing of rats in a uniform environment (a large open field) in a standardized paradigm under standardized conditions. Across all experiments, the library has a total of 19,976 trials of rat behavior in an open field apparatus. The duration of each test is 55 minutes. In total, this constitutes over 2 years of continuous recording. In addition to the raw data videos of each trial, the library includes two video-derived raw data objects: XY locomotion coordinates, and plots of animal trajectory. Importantly, these 3 data objects were annotated in a manner that functionally abstracts the trials from the context of the particular experiment and hence the library as a whole constitutes one large dataset of trials with well-specified independent variables.

HISTORICAL BACKGROUND

In an open field the spatiotemporal pattern of behavior of rats treated chronically with the dopamine agonist quinpirole (QNP)[26] is strikingly transformed compared to the behavior of rats treated with saline (see Figure 1). A series of studies showed that this transformation constitutes an animal model of OCD because quinpirole-induced behavior possessed the structural features of compulsive checking shown by individuals diagnosed with OCD [25, 27-30]. In the animal model, compulsive checking is manifested by exaggerated preoccupation with one location in the environment, to which the animal returns repeatedly. A number of reviews of the model had been published; e.g., [31-41].

This animal model had been used in the laboratory of the principal investigator (HS) as the research preparation for investigations of OCD, addressing questions such as the neural circuits mediating compulsive checking, their neurochemistry and pharmacology, possible drug treatments, biological markers of the disorder, factors that modulate the development and the expression of compulsive checking, and what are the constitutive components of compulsive behavior. Altogether these investigations can be grouped into 12 projects that encompass 43 experiments. Table 1 lists the titles of those projects and shows how the 43 experiments are distributed amongst the projects. (Table 1 includes additional columns that are considered later).

Note that titles in Table 1 often mention "sensitization", in addition to "compulsive checking". This is because repeated injections of QNP induce also locomotor sensitization [42-48]. For this reason, the QNP preparation is often called the "quinpirole sensitization rat model of compulsive checking" [41] and studies of compulsive checking may concurrently encompass research on mechanisms of sensitization.

Below we describe the organization and properties of the 43 datasets derived from this research. Given the above claim that the raw data objects should be viewed as "one large dataset," it may be asked why there are 43 separate dataset deposits and not a repository of

only one dataset? This is because there is no convenient infrastructure to host a searchable dataset that is 11 terabytes with close to 60,000 data objects. Moreover, currently, most dataset deposits are intended to supplement a published report and are of data from a single study or project; typically, the dataset deposit includes an account of the research question and a detailed description of the experimental methods. Here we follow this template as the raw data objects were obtained piecemeal in the context of separate and distinct studies on mechanisms of OCD. The notion that the collected raw data objects are a large dataset emerged after the close of active research when the principal investigator considered the utility of making the collected video and video-derived data objects available in a public repository. Hence, the approach taken was to adapt the standard practice of separate datasets for different studies, but use a common annotation schema across all datasets.

STANDARDIZED PARADIGM

Studies using the animal model employed a standard apparatus and followed a standard protocol described below; details of particular experiments are provided separately in the "Readme_Experiment##_Q##.txt" files deposited with each dataset on the GigaDB website.

Together with describing the paradigm, we consider the annotation schema for data objects in the library. As noted in the Introduction, the motivation underlying such annotation was to create one large dataset, by functionally abstracting the open field trials from their specific experiments. Our approach towards this aim was to emulate the structure of the methods section of a behavioral neuroscience research report.

A generic Methods section of a research report includes headings such as Apparatus, Subjects, Treatment, and Procedure. Accordingly, metadata variables were constructed that would capture the information within those headings. Metadata variables were named according to a hierarchical schema, where the top of the hierarchy was a heading category. For Apparatus, Subjects, and Treatment, the corresponding name prefix of the metadata variables were APPARATUS_, SUBJECTS_, and TREATMENT_. For the Procedure category, the hierarchical prefix was TRIAL_ as annotations of a trial would provide the procedural information.

As will be apparent, often the same independent variable is annotated twice: as a numeric value (VariableName_value) and as the corresponding label for this value (VariableName_valueLabel). This is to permit more convenient search using numeric code, rather than long strings of text.

Below, we use the template of a generic research report with Apparatus, Subjects, Treatment, and Procedure as headings. Within each category we provide the pertinent information for studies using the animal model together with an explanation of the corresponding metadata

variables. Much of the pertinent information was collated from our published reports [29, 49-59]. Note that those reports were not accompanied with the publication of datasets containing the raw data objects described in the present Data Note paper.

APPARATUS

OPEN FIELD

In investigations of compulsive checking, rats were tested in a large open field consisting of a table (160 x 160 and 60 cm high) that was standing at least 70 cm away from walls. The table top was constructed of material used in making kitchen counter-tops – it was smooth, non-porous, composed of unsaturated polyester and acrylic resin blends (Acryflek Industries), and had a custom blue color to facilitate video detection of dark and white objects. Four small boxes were present at the same fixed location of the open field throughout the study: two at the corners and two at places near the center of the open field. Three of the boxes were 8-cm Plexiglas cubes (two clear and one black), all of which had the tops painted blue to match the table and contained weights to secure the objects to the table. All of the cubes had the top half of one side open to allow the rat to put its head and front paws inside the box. The fourth object was a rectangular glass container (10.5 cm x 8.5 cm x 7.0 cm) with the top side open but covered with a wire mesh; the glass dish was secured to the table with translucent caulking (see Figure 2).

Two such open field tables were located in one testing room, and two tables in another room. The identity of these open fields are annotated by the meta variables **APPARATUS_ArenaID_value** and **APPARATUS_ArenaID_valueLabel**, with the values 1 to 4 corresponding to labels “OF #1 in room U59_south”, “OF #2 in room U59_north”, “OF #3 in room U60_south”, and “OF #4 in room U60_north”, respectively. The address of the testing room (U59/U60) refers to its designation in the Central Animal Facility in the Health Sciences Center building at McMaster University; north/south refers to open field location in the testing room (“north” is in the half of the room near the door and “south” is in the other half of the room).

The four small objects in the open field were moved elsewhere for a trial called the “object rotation test” [25]. This test evaluated whether rats showing compulsive behavior are aware of their surroundings, like are OCD patients who despite their compulsions remain well connected with the environment and reality [60]. To assess awareness of the surroundings, the position of the four objects was rotated in space by 180 degrees (see Figure 3), with the expectation that this should alter behavior.

The annotation of where in the open field the four objects were located is done under the metadata variables **APPARATUS_ArenaObjects_value** and **APPARATUS_ArenaObjects_valueLabel**. The annotation for the standard fixed position of the four items is given by the code value "1" for **APPARATUS_ArenaObjects_value** and by the corresponding label "objects in arena locales 10 14 5 and 8" for **APPARATUS_ArenaObjects_valueLabel**. The annotation of these variables for the "object rotation test" is: "2" for **APPARATUS_ArenaObjects_value** and "objects in arena locales 18 22 9 and 4 (objects ROTATION 180 deg)" for **APPARATUS_ArenaObjects_valueLabel** (Table 2).

Because compulsive behavior is strongly modulated by environmental cues [25, 28, 30], an experiment evaluated the effect of removing all the objects from the open field. For those trials, **APPARATUS_ArenaObjects_value** had the value "0" and **APPARATUS_ArenaObjects_valueLabel** had the label "no objects in arena".

Table 2 shows that for 18,689 trials in the open field, the four small objects were in their standard location, while for 879 trials the items were shifted in space 180 degrees; for another 55 tests the open field was empty of any items in it. Moreover, Table 2 shows that there are more tests in OF#1 and OF#2 than in OF#3 and OF#4. This reflects the fact that at the start of this research only one testing room was available.

Aside from the standard open field arena, Table 2 lists three other ones (see variables **APPARATUS_ArenaType_value** and **APPARATUS_ArenaType_valueLabel**). One arena is the same as the standard open field except that edges of the table were fenced by a 5 cm high Plexiglas border. This tiny fence was erected to determine whether it would eliminate jumping or falling off the open field, which some rats may do and which necessitates the presence of an experimenter in the testing room to place the animal back onto the open field. The pilot experiment of 208 trials (Table 2) showed that the number of falls/jumps was reduced but not eliminated, indicating the need for an experimenter in the room. Moreover, it was found that the Plexiglas fence made it more difficult to clean the open field after each trial because when rats urinated near the fence the urine seeped to the joint between the Plexiglas wall and table edge, making it difficult to wipe clean.

The remaining two types of arenas listed in Table 2 under the variables **APPARATUS_ArenaType_value** and **APPARATUS_ArenaType_valueLabel** are a circular open field (220 cm in diameter and with a 50 cm high wall) and a small locomotor activity chamber (Plexiglas box, 40x40x35 cm; AccuScan Instruments, Columbus, Ohio). The number of video data objects from those two arenas is limited, 140 and 5 respectively.

TESTING ROOM

The two open fields in each testing room were partitioned from each other by curtains, such that the two rats were not in visual contact during testing. Moreover, the experimenter was located in the space between the two open fields, so when the two curtains were drawn, the experimenter was also isolated from visual contact with the rats. The experimenter recorded and watched the rats' activity on a monitor-recording set up, located on shelves in the space between the two open fields. Photographs of the experimental set up are shown in Figure 4.

For all but 55 trials, the testing room was illuminated by its ceiling fluorescent lights, and this provided the lighting required for video recording. For 55 trials, the ceiling lights were off and the room was in darkness; infrared lights provided illumination for video recording (these trials were motivated as a pilot run to examine whether as found in intact rats [61], there is a shift from directional to positional progression under quinpirole; regrettably, an analysis of these trials was not completed). The state of illumination of the testing room at the time of the trial is annotated by the variables **APPARATUS_ArenaTestingRoomLightCondition_value** and **APPARATUS_ArenaTestingRoomLightCondition_valueLabel (**Table 2). This information is recorded also in the GigaDB Sample Tab under the variable "exp_scan_method"[a], which includes a notation that the rat was tested during its "subjective night/sleep" (the day/night cycle in the colony room was lights ON from 7 AM to 7 PM). The light/dark cycle in the colony room is annotated with the metadata variables **SUBJECTS_LightDarkCycleInColonyRoom_value** and **SUBJECTS_LightDarkCycleInColonyRoom_valueLabel**. The actual time and date of the trial is annotated with the variable **TRIAL_DateTime_str;** this information is duplicated in the GigaDB Sample Tab under the variable "collection_date".

VIDEO RECORDING

Up to March of 2015, behavior in the open field was captured on video with a stationary overhead camera interfaced with a consumer grade VHS video-cassette recorder (vcr). There was one such camera-vcr system for each one of the 4 open fields. At end of testing, the video cassettes were brought to the laboratory and converted to MPEG files using "Canopus MPEGPRO EMR - video capture adapter - USB 2.0" at a resolution of 352x240 pixels or a PCI-based version of the capture device (MPEGPRO MVR), also at a resolution of 352x240 pixels. MediaCruise software supplied with the hardware was used to control the conversion to digitized video. Overhead CCD cameras (Ikegami CD-47 for open field #1 and Panasonic WV-BP334 for open fields #2, #3 and #4) provided an analog B&W video signal at the rate of 29.97 fps that was recorded by the vcr on video cassettes. After completion of a study, the MPEG video files were burned on DVD for archival storage.

In subsequent years, videos of behavior in the open field were recorded as MPEG files directly. The CCD cameras were interfaced with a PC computer that captured the video signal and stored

the videos as MPEG files on the hard drive. There was one such camera-computer system for each open field. For open fields 1 and 2, the video signal was captured with "Canopus MPEGPRO EMR - video capture adapter - USB 2.0" at a resolution of 352x240 pixels while for open fields 3 and 4, a PCI-based version of the capture device was used (MPEGPRO MVR), also at a resolution of 352x240 pixels. MediaCruise software supplied with the capture device hardware was used to control the video recording. The MPEG files from the computers in the testing rooms were transferred to computers in the laboratory for offline analysis. Table 3 gives the number of trials recorded with the camera-vcr and camera-computer systems. The annotation identifying which video recording system was used is found under the metadata variable “exp_scanner”.

The frame rate of 29.97 fps for all videos is annotated in the GigaDB Sample Tab under the variable “exp_scan_parameters”. The resolution of all videos (352x240 pixels and 8x8 mm per pixel) is annotated under the variable “exp_scan_resolution”.

SUBJECTS

Subjects in all 43 experiments were adult male Long-Evans rats, sourced from a single supplier. They were housed individually with food and water freely available in a climate controlled colony room on a 12 hour light/dark cycle. The home cage was a polyethylene cage (35 cm x 30 cm x 16 cm) lined with absorbent bedding material but otherwise empty of any items (see bottom photo in Figure 4). However, the housing condition was altered slightly for the last two studies in the library (Study Q47 and Q48). Specifically, the home cage was altered to an “enriched” housing environment, by placing inside the cage a black plastic tube that the rat could crawl into or play with. A comparison of study Q47 with Q46 showed that "enriched" housing did not influence the development of sensitization or compulsive checking. In all studies, animals were housed and tested as approved by the Animal Research Ethics Board, McMaster University in compliance with the Canadian Council on Animal Care guidelines.

Most of the above details were annotated under self-evident metadata variables in the GigaDB Sample Tab (“Taxonomic_ID”,”Common_name”,”Genbank_name”,”Scientific_name”, “genotype”,”age”,”sex”,”Strain”,”subject_supplier”, **SUBJECTS_LightDarkCycleInColonyRoom_value**, **SUBJECTS_LightDarkCycleInColonyRoom_valueLabel**, **SUBJECTS_LightDarkCycleWhenTested_value**, **SUBJECTS_LightDarkCycleWhenTested_valueLabel**, **SUBJECTS_HomeCageInColonyRoom_value,** **SUBJECTS_HomeCageInColonyRoom_valueLabel**). However, two more annotations pertaining to the category of Subjects should be mentioned.

First is the metadata variable **SUBJECTS_RatUniqueID**, which holds a unique 10 characters identifier for every rat in the library. For consistency with GigaDB nomenclature, this ID is duplicated under the variable "host_subject_id". Using this ID, it is possible to follow the performance of an individual rat during repeated testing. Moreover, a count of unique IDs will give the total number of rats in the library (1758 rats).

Second is the metadata variable **SUBJECTS_RatUniqueID_BodyWeightAtTRIAL_str**. This variable holds the body weight in grams of the rat before the trial. Rats were weighed because using the animal model involved drug treatment, and the amount of drug administered was on a per kilogram basis (mg/kg). Weight was annotated as string to allow for text input indicating missing values, according to GigaDB format.

TREATMENT

Considering that the preparation of the animal model of OCD required repeated injections of quinpirole, studies with this model necessitated some type of drug treatment. Indeed, drug treatment is a characteristic of all data objects in the library. For some data objects there was a second treatment: brain manipulation. This treatment was employed in studies that probed the neural underpinnings of OCD (Table 1). Below we describe the two types of treatment and how they were annotated.

DRUGS

The canonical preparation to induce compulsive checking involved 8 to 10 injections of quinpirole (0.5 mg/kg) administered 3-4 days apart; the rat was placed in the open field after each injection for 55 min. Control rats received an injection of saline. Studies that addressed questions of neurochemistry and pharmacology of OCD, pharmacotherapy, or constitutive elements of OCD, utilized a variety of drug treatments. Below we describe how those drug treatments were annotated.

Table 4 shows that across the 43 experiments rats were injected with 9 different drugs (clorgyline, CP809101, DPAT, escitalopram, imipramine, mCPP, QNP, ritanserin, U69593) and three different control substances (saline and two other vehicle preparations). These formed 20 different drug treatments because drugs were injected either alone or in some combination of two or even three drugs. As shown in the table, the two highest numbers of trials were with injections of QNP (8012 trials) and with injections of SAL (7551 trials).

The metadata variable **TREATMENT_DrugRx_NameOfDrugs** (column 1 in Table 4) specifies the prescribed drug treatment, that is, name(s) of drug(s) injected before a trial. When the prescribed treatment was a single drug, **TREATMENT_DrugRx_NameOfDrugs** was annotated

with the name of the drug, or with the name of the control substance (typically, saline). When the prescribed treatment was co-injection of two or three drugs, then **TREATMENT_DrugRx_NameOfDrugs** was annotated as Drug1+Drug2 or Drug1+Drug2+Drug3. For treatment with two drugs, the prescribed control conditions were Drug1+SAL, SAL+Drug2, and SAL+SAL; however, the corresponding annotations for **TREATMENT_DrugRx_NameOfDrugs** were Drug1, Drug2, and SAL. That is to say, annotations of drug treatment for prescribed control conditions ignored co-injected SAL. Similarly, with three drugs in an experiment, **TREATMENT_DrugRx_NameOfDrugs** was annotated in control trials as Drug1+Drug2 or Drug1+Drug3 or Drug2+Drug3 or Drug1 or Drug2 or SAL.

Drug treatment ought to be considered together with annotations of another variable, **TREATMENT_DrugRx_1or2or3substancesInjectedBeforeTest**. This metadata variable, which may take a value from 1 to 3, has a dual role. First, for the experiment, it specifies whether the prescribed drug treatment was comprised of one drug, or co-injections of two drugs or co-injections of three drugs. Second, it specifies the number of items injected before a trial, including co-injected saline. Hence, if for example, **TREATMENT_DrugRx_NameOfDrugs** was "QNP" and **TREATMENT_DrugRx_1or2or3substancesInjectedBeforeTest** was "2" then before the trial QNP and SAL were co-injected.

Table 4 includes a breakdown of drug treatment by dose, shown under **TREATMENT_DrugRx_SubstancesAndDosesInjected** (column 2). This variable, for prescribed drug treatment with 1, 2, and 3 drugs, had the values "Drug1/ControlSubstance1 Dose", "Drug1/ControlSubstance1 Dose and Drug2/ControlSubstance2 Dose", and "Drug1/ControlSubstance1 Dose and Drug2/ControlSubstance2 Dose and Drug3/ControlSubstance3 Dose", respectively. Dose is amount of the drug in mg/kg.

All drug doses were administered at a volume of 1.0 ml/kg and injected subcutaneously under the nape of the neck. Equivalent volumes of vehicle were used for control injections. With two exceptions, all drugs were dissolved in physiological saline and hence their control injection was saline (SAL) and the dose necessarily 0 mg/kg. The first exception was U69593, which was dissolved in sterile water containing 20% propylene glycol (vol/vol), and hence this vehicle was used for control injections; it is annotated as "VEHa 0" under **TREATMENT_DrugRx_SubstancesAndDosesInjected**. The other exception was ritanserin, which was dissolved in a vehicle containing 67% ethanol and 33% saline; this control substance is annotated as "VEHb 0".

Inspection of Table 4 shows that a particular drug treatment may include several different doses of the drug; for example, QNP was administered at 7 different doses, ranging from 0.0312 mg/kg to 0.5 mg/kg. Moreover, a particular drug treatment may include trials with co-injections of a control substance; for example, QNP, which was administered on 8012 trials, was

co-injected with saline (SAL) on 1189 trials and with another control substance (VEHa) on 144 trials.

Are the effects of a drug different from the effects of the drug co-injected with saline (or another vehicle)? The design of our experiments did not address this question. However, our impression has been that if any effects exist, they are very subtle. The same reasoning applies to possible differences between one and two needle pokes for treatment with SAL.

As noted above, the canonical preparation to induce compulsive checking involved 8 to 10 injections of quinpirole. Hence, datasets in the library have another salient characteristic, namely, repeated drug treatment, which is annotated under **TREATMENT_DrugRx_Number_str**. Annotations of this metadata variable specify how many injections of the prescribed drug(s) the rat received before being placed on the open field (current trial injection is included). As expected, across all experiments, the number of repetitions of treatments with QNP or SAL was high: 617 trials with 10 injections of QNP and 580 trials with 10 injections of SAL; the maximum number of QNP treatments was 14 (59 trials) and the maximum number of SAL treatments was 15 (18 trials). This information is provided in Table 5 which shows the number of trials that a given drug treatment was administered on successive injections of it.

In some tests of compulsive checking or behavioral sensitization, there was a change in the prescribed drug treatment; for instance, when examining conditioned effects, rats were injected with SAL (rather than the drug). For those trials, the value for the variable **TREATMENT_DrugRx_Number_str** became 1 since that was the first exposure to the new treatment. If further testing continued with the original drug treatment, then counting resumed from the value before treatment change.

LESIONS

Table 1 lists experiments which probed the neural underpinnings of OCD; several of those studies had been published [51, 52, 55, 57, 62]. The data objects from those experiments had, in addition to drug treatment, a second one, namely, a surgical procedure to lesion a specific part of the brain (region of interest; ROI). This treatment was annotated using two sets of metadata variables.

The first set consists of the variables **TREATMENT_BrainSurgery_manipulation_value** and **TREATMENT_BrainSurgery_manipulation_valueLabel**, with the former coded as a value and the latter as the label for this value. Annotations of these variables specify the anatomical targets of brain surgery, namely, Basal Lateral Amygdala (BLA), Infralimbic Cortex (ILC), Nucleus

Accumbens Core (NAc), Orbital Frontal Cortex (OFC) and the Pituitary; a notation for sham surgery is also included (Table 6).

Neurotoxic lesions aimed at BLA, ILC, NAc, and OFC were performed using the excitotoxin N-methyl-D-aspartate (NMDA) that was dissolved in phosphate-buffered saline (PBS) and administered via intracranial injection using a 10 µl non-coring Hamilton syringe mounted in a motorized Ultra Micro Pump (World Precision Instruments, U.S.A.) attached to the arm of a Kopf Stereotaxic Apparatus (David Kopf Instruments, U.S.A.). For sham lesions, an equivalent volume of PBS was injected. Surgery to remove the pituitary was done by Charles-River, Canada using the parapharyngeal approach.

In all, using the metadata variables **TREATMENT_BrainSurgery_manipulation_value** and **TREATMENT_BrainSurgery_manipulation_valueLabel**, 4058 trials were annotated with a target ROI and another 2269 trials with "sham" lesion as the target (Table 6). Data objects for the remaining trials in the library (13,649 trials) were annotated with "No surgery done" under these variables (Table 6).

The second set consists of the variables **TREATMENT_BrainSurgery_HistologyFindingsOutcome_value** and **TREATMENT_BrainSurgery_HistologyFindingsOutcome_valueLabel**, coded as value and label, respectively. These variables annotate findings from histology, indicating degree of success in destroying ROI cells. As shown in Table 6, there were three different annotations for the success of surgery: "Lesion does NOT meet criteria", "Lesion meets criterion of at least 55% of ROI lesioned bilaterally", and, "No lesion present", the latter indicating that despite surgery to administer NMDA into the brain, there was no evidence of cell loss, equivalent to a sham lesion. "No lesion present" was annotated also for trials where rats received no brain surgery.

Table 6 shows that these variables had two more annotations: "Histology not available" and "Complete hypophysectomy". The former annotation was applied to brain lesions not verified by histology due to technical difficulties. The latter annotation indicated successful removal of the pituitary, as confirmed by no gain in body weight [51, 63].

PROCEDURE

As noted before, repeated testing in the open field was a salient attribute of studies using the animal model of OCD. Properties of test repetition were annotated using several metadata variables, the names of which all start with the prefix TRIAL_. Below we described those variables and their annotations.

An Experimenter was present in the testing room throughout the trial, but hidden behind a curtain from the animal's sight. Experimenter observed activity of the rat on a TV monitor and

retrieved the rat if it fell or jumped off the arena. The rat was generally placed back at the spot from which it left the arena.

Any written observations of the trial made by the experimenter were transcribed and incorporated as notes for the trial in EthoVision software and became part of the record for the trial.

Several metadata variables were used to annotate particulars of the trial or of the video recording session, as described below.

TEST NUMBER

Typically, in experiments using the animal model, the rat is administered the prescribed drug treatment, placed on the open field, and the trial recorded for 55 min. Hence, it would seem that **TREATMENT_DrugRx_Number_str** described above should also indicate the sequential number of the current trial. However, this is not the case because on some tests there was a change in treatment, resetting **TREATMENT_DrugRx_Number_str** to “1”. For instance, on a test for conditioned activity, rats treated chronically with quinpirole received an injection of saline (rather than quinpirole) and their behavior compared to rats treated chronically with saline; similarly, on a test for sensitization, rats treated chronically with saline received an acute injection of quinpirole and their response compared to the chronic quinpirole response (see, for example [52]). Clearly, these tests were not the first trial in the open field.

The situation is even more complex because in some experiments, the standard protocol of testing the rat in the open field after every administration of drug treatment was not followed. Rather, the rat was also tested in an alternate environment (e.g., [49]). In still other experiments, the rat was placed for the first time on the open field only after chronic drug treatment in a different environment. For instance, in an experiment aimed to separate the effects of mere exposure to quinpirole from the effects of exposure to quinpirole in the open field, study Q27 (Table 1) compared the effects of chronic drug treatment in the home cage, the activity chambers, and the open field on performance of compulsive checking when all rats were tested in the open field (results showed that on the test in the open field rats which received chronic treatment in the open field rats showed robust compulsive checking, rats which received chronic treatment in activity cages showed some degree of compulsive behavior on the open field test, and rats which received chronic drug treatment in their home cage showed little to none compulsive checking in the open field test). Clearly, of relevance to the annotation schema, test number in the open field can differ from the number of drug treatments administered.

To account for such different situations, two metadata variables were used: **TRIAL_InjectionNumber_str** and **TRIAL_OFtestNumber_str**. The first one keeps a running tally of how many times drug treatments were administered, that is, how many treatment injections the rat received before the current trial (injection just before the current trial is included). The second one keeps a running tally of how many times the rat was tested in the open field (the current trial is included). For most data objects in the library both variables have the same tally during repeated testing, but when not, then the rat was not tested in the open field following one or more drug treatments.

There are 5 trials where rats were not filmed in an open field but rather in a different arena, Plexiglas box (Table 2). Corresponding to the metadata variable **TRIAL_OFtestNumber_str**, the metadata variable **TRIAL_AMtestNumber_str** tracks the number of tests in the activity monitor Plexiglas box.

Note that juxtaposition of the three variables (**TREATMENT_DrugRx_Number_str**, **TRIAL_InjectionNumber_str** and **TRIAL_OFtestNumber_str**) provides opportunity to evaluate factors which may contribute to the effect observed with repeated drug treatment in the same environment.

DATE, TIME, AND DURATION OF TRIAL

The date of the trial and its starting time were annotated under the variable **TRIAL_DateTime_str**, formatted as YYYY-MM-DD HH:MM:SS. Together, this variable and **TREATMENT_DrugRx_Number_str** give the schedule of prescribed drug treatments.

Start time of some trials were not logged, and for those trials the missing time is indicated as 00:00:00. **TRIAL_DateTime_str** is duplicated in the GigaDB database as “collection_date”.

Each trial was 55 min long and this duration was annotated as the numeral 55 under the variable **TRIAL_duration**.

OBSERVATIONS DURING TRIAL AND NOTES

The person responsible for testing was instructed to note anything of potential impact on the rat’s performance. Those observations, if any, were annotated under the variable **TRIAL_ObservationsAndNotes**. The metadata variable **TRIAL_ObservationsAndNotes** included also any notes on the trial made by others in the workflow of processing the video file.

In preparing the datasets for deposit, the principal investigator extracted from the notes two pieces of information, for annotation separately under the variables **TRIAL_FallsDuringTest_Number_str** and **TRIAL_FallsDuringTest_TimeWhenFell**. The former

variable recorded the number of times that the rat fell or jumped off the open field, and the latter recorded at what time the event occurred. The annotated time is in minutes from start of the trial; if more than one fall occurred then the times are given as a list of space separated numbers. Note that if notes did not mention falls, then the number of falls was annotated as "not provided" rather than as 0. Conceivably, there was a failure to record the event; if experimenter noted that there were no falls during the trial then 0 was annotated for **TRIAL_FallsDuringTest_Number_str**.

PERSON CONDUCTING THE TRIAL

An aspect of the protocol using the animal model was to maintain consistent conditions for open field testing since that seemed to provide robust performance. With this in mind, individual rats were assigned to be tested in the same open field, at the same time of day, and handled by the same experimenter. Individual rats were consistently tested in the same open field, but scheduling issues did not permit a strict adherence as to the time of day or the same experimenter. Variations in time of day can be discerned from annotations in the time portion of **TRIAL_DateTime_str**. Consistency in handing a given rat can be discerned from annotations under the variable **TRIAL_ExperimenterNAME**, which provides First LASTNAME of the person conducting the trial, with last name in CAPS as per GigaDB format.

TYPES OF DATA FROM TRIAL

A trial generated three types of raw data: a video of the trial (file with mpg extension), the time series of XY coordinates of locomotion (file with csv extension), and a plot of the trajectory of locomotion (file with gif extension). However, for technical reasons, the library does not have all three raw data objects for every trial—some trials may lack one or two data types. What data types or files are actually available for a particular trial is annotated under **TRIAL_RAWDATAObjectsAvailable_value** and **TRIAL_RAWDATAObjectsAvailable_valueLabel**. Table 7 shows that for the majority of trials (18436 of 19976 trials) all three data objects are available and that for only 928 trials a video of the trial is not available. Generally a video was unavailable for deposit because the file archived on DVD was corrupted and could not be restored when depositing files into the repository.

ANNOTATIONS SPECIFIC TO VIDEO FILES

When behavior in the open field was filmed using a camera-vcr system, multiple trials were recorded on a single VHS cassette. This, in turn, resulted in a single digitized video file (mpg) with up to 6 tests. Information specific to the video file was annotated under three variables described below.

One key variable was **VIDEOinfo_FrameStart2trackRat_str**; it marked the position in the video file when a trial begins. A trial started at the moment the rat was released onto the open field. The frame in the video file when this occurred was noted and the number of the frame annotated under **VIDEOinfo_FrameStart2trackRat_str**; this frame number was also the point from which EthoVision software would start tracking the XY coordinates of locomotion. Each video file with more than one trial would have associated with it multiple instances of **VIDEOinfo_FrameStart2trackRat_str**, one for each trial.

For documentation, a blackboard with some pertinent information was filmed preceding the trial. This information included a code for the rat and its experimental group/drug treatment, annotated under **VIDEOinfo_RatIDonVideoBlackboard** and **VIDEOinfo_ExptGroupOnVideoBlackboard**, respectively. However, in the context of the library, the variables on the blackboard are of limited value. This is because information on the blackboard was re-mapped into a schema that was consistent across all studies. In particular, rat ID and experimental group were annotated under the variables **SUBJECTS_RatUniqueID** and **QSTUDYinfo_ExptGroups**, respectively, as elaborated later.

IDENTIFICATION METADATA VARIABLES

There were two, redundant, housekeeping variables used for data management: **ID7STRING** and **TRIAL_ID**. **ID7STRING** was assigned as the primary key variable to every record maintained in a flat SPSS database. Its value was incremented by one for every new trial read into the database.

A 28 characters long unique code for every trial was also constructed, intended to provide more informative identification. It is annotated under the variable **TRIAL_ID**, and was generated according to a specific rule that is shared with the naming convention for track and path plot files; the rule is described below in the section on naming raw data objects.

TRIAL_ID is duplicated as “Sample_ID”. In the GigaDB database, the variable “Sample_ID” refers to the unique identifier of a particular “sample”, usually a physical entity. The data objects (files) from our experiments also derive from a “sample”, but the sample is of “performance in a specific environment in a specified length of time”, and as such the sample is an event entity, called here a “trial”. Hence, **TRIAL_ID** and “Sample_ID” are equivalent and the two variables are interchangeable.

ANNOTATIONS OF ADMINISTRATIVE AND HISTORICAL VARIABLES

In our research using the animal model each new study was assigned a 3 character code “Q##” that ranged from Q17 to Q48 for datasets in the library. This study code was annotated under the metadata variable **QSTUDYinfo_3characterCode**.

In addition to the Q## code, each study had an identifier 4 character label that was a tag for the type of experiment in the study. This label was annotated under the metadata variable **QSTUDYinfo_Expt4characterCode**. Most Q## studies had only one such label but studies Q25, Q26, and Q28 had two. Those studies used the lesion approach to probe the role of a brain region (region of interest; ROI) in compulsive checking, and addressed two separate questions: What is the ROI role in the *development* (pathogenesis) of compulsive checking, and what is its role in the *expression* (maintenance) of compulsive checking? For the first question, the ROI was lesioned and the effects of the standard protocol on the induction of compulsive checking monitored. For the second question, the standard protocol to induce compulsive checking was administered first, the ROI was lesioned, and the effects of the lesion on the performance of compulsive checking measured. To distinguish between those two types of experiments, **QSTUDYinfo_Expt4characterCode** consisted of a 3 character ROI label and a “1” for a development experiment and a “2” for an expression type of experiment.

The two metadata variables, **QSTUDYinfo_StudyTitle** and **QSTUDYinfo_ExptTitle**, contain, respectively, title of Q## study and title of the experiment in Q## (Table 1). A Q## study may contain more than one experiment either by proper design of a second experiment or by virtue of a pilot experiment that continued with the testing of rats beyond experiment proper.

The metadata variable **QSTUDYinfo_ExptGroups** is annotated with the name of the experimental group to which the indicated trial belongs. Annotations of this variable use a long descriptive name of the experimental/control group, corresponding to codes annotated under **VIDEOinfo_ExptGroupOnVideoBlackboard**.

In preparations to deposit the datasets in a repository, an attempt was made to group the various research studies into themes or projects. This grouping into projects is annotated under the metadata variables **QSTUDYinfo_Project_value** and **QSTUDYinfo_Project_valueLabel**, with the latter containing the label or title of the project and the former containing a numeric code for this title (Table 1). The numeric codes range from 1 to 12, but these ordinal values do not represent ordering of an attribute. The highest number of trials in a project is 7037 trials (Project 1) and the lowest 137 (Project 6) (see Table 1).

The 43 experiments in the library were manually assigned a number from 1 to 43 (annotated under **QSTUDYinfo_ExptNumberInProject**) according to their position in the sorted list of projects from 1 to 12 (Table 1). Within a project, ordering of experiments followed aims of the experiments or historical precedence.

It is worth highlighting that the "**QSTUDYinfo_**" annotations are not relevant to the framework of a library with one large dataset. This is because, by design, data objects in the library were annotated in a manner that would abstract them from the particulars of an experiment. For example, explicit group membership (**QSTUDYinfo_ExptGroups**) is not needed because pertinent information is annotated as distinct independent variables. Similarly, the titles of experiment/study/project conveying the intent of the experiment which generated the data objects are not relevant because the key independent variables with impact on behavior were annotated separately. Nevertheless, these historical variables are provided as acknowledgement that the data objects are contained in deposits of distinct datasets and to give a historical context in which the data objects were generated.

RAW DATA OBJECTS

THREE TYPES OF DATA OBJECTS

As noted in section "Types of data from trial", datasets in the repository contain files with three extensions: mpg, csv, and gif. Files with the first extension are raw data videos of the trial and files with the other two extensions are video-derived track and path plot data objects, respectively. Below is additional information on those files, including an explanation of the rules in naming them.

VIDEO FILES

A typical recording session of a trial began with the experimenter taking a video of a blackboard with particulars of the trial, and then placing the injected rat in the center of the open field, facing the far edge (locales 23-25; see Figure 4, middle row, right photo). Experimenter removed the rat from the arena after it was there for 55 min. Experimenter then stopped the video recording.

TRACK FILES

In studies using the animal model of OCD, we derived measures of compulsive checking from the trajectories of locomotion in the open field. Thus, the first step for this endeavor was to obtain the coordinates of locomotion. This was accomplished with EthoVision (Noldus Information Technology bv, Netherlands) software (version 3.0/3.1) [12, 64]. The program extracted the XY position of the rat from each frame of the video and the time series of those coordinates was exported from EthoVision as a comma-delimited (csv) tabular track file. Track files were processed further with custom software to derive the criteria measures of compulsive checking [29, 52]. However, data from processed track files are not included—only the raw unfiltered time series of XY coordinates are in the library.

Importantly, for every study, each open field was calibrated separately in EthoVision (see Figure 5). Therefore, the exported XY tracking coordinates refer to the same Cartesian space, regardless of any image differences introduced by separate video recordings of the open fields.

The list of sample data points in the csv track files is preceded by a header line, which can be either “Sample no.,Time,X,Y,Area,ZONES”, or, “Sample no.,Time,X,Y,Area,ZONES,Distance moved,Velocity”. However, the relevant raw data fields are just “Time,X,Y,”. "Sample no." is equivalent to "Time". The other columns are variables computed internally by EthoVision; they were not exported consistently across studies. Importantly, in our analyses, we made use and processed just the “Time,X,Y,” columns.

Missing values in the list of coordinates in the csv file are indicated by -. Missing values may have occurred because the animal fell or jumped from the open field and hence was absent for part of the trial, or artifacts were deleted and could not be replaced by valid points.

The EthoVision .trk track files were set for export as csv files together with all the independent and system variables. Those variables are at the top of the csv file, preceding the header line “Sample no.,Time,X,Y,Area,ZONES”, or, “Sample no.,Time,X,Y,Area,ZONES,Distance moved,Velocity”. Those exported variables have two comma delimited columns, with the first column specifying the name of the variable and the second column indicating its value. These variables should be ignored as the relevant ones are annotated as metadata variables.

PATH PLOT FILES

A second video-derived data object is a plot of the time series of XY points, representing the locus of locomotion in 55 min. This graphic was generated in Mathematica [65], after smoothing of the coordinates to remove noise [66], and was saved as a static image file with the gif extension. In our workflow, these path plots served to provide a graphic record of performance in the open field and as a tool to spot errors or artifacts, necessitating a re-processing of the video with EthoVision.

In the path plot image, the trajectories of locomotion are displayed on top of a virtual grid superimposed on the open field (Figure 3). The virtual grid was used for computations of checking behavior [29, 52] but the raw data objects in the library do not require or depend on this grid.

The 4 green squares in the path plot image indicate location of the 4 small objects. However, this representation is not true in path plot images from the “object rotation test” when the 4 objects were in a different location. In images from those tests, the Mathematica script that rendered the trajectories of locomotion was not programmed to relocate the green squares to their new position (Figure 3).

Finally, the ID of the trial is shown at the top of the path plot image (Figure 3). This ID is annotated by the metadata variable **TRIAL_ID**, and is duplicated in the GigaDB Sample Tab under the variable “Sample_ID”.

NAMING CONVENTION

Files of the three raw data objects were named according to a convention that allows for ready identification of the trial.

VIDEO FILES (MPG)

The name for a video file was a concatenation of 6 elements and extension “mpg”. These elements are described below, using for illustration the video file name “Q30DRCq_of1_inj01_20080623_029_050_020_052.mpg”:

- Column 1-3 contains a code for the study, given by **QSTUDYinfo_3characterCode** (here, Q30)
- Column 4-7 contains a unique 4 character label for an experiment in the study, given by **QSTUDYinfo_Expt4characterCode** (here, DRCq)
- Column 8 has the underscore character (_) to mark the separation from the next code
- Column 9-11 shows in which arena the rat was tested (here, of1); “of” indicates “open field” and “1” is the value given by **APPARATUS_ArenaID_value**
- Column 12 has the underscore character (_) to mark the separation from the next code
- Column 13-17 shows the injection # (here, inj01); “inj” indicates “injection #” and “01” is the value given by **TRIAL_InjectionNumber_str** (number format is 2 characters string)
- Column 18 has the underscore character (_) to mark the separation from the next code
- Column 19-26 contains the date of the trial, given by **TRIAL_DateTime_str** but includes only the date portion formatted as YYYYMMDD
- After the date of the trial, there is a list of rats in the video file, separated by the underscore character (_). Here, there are 4 rats but there may be 1 to 6 rats recorded in a video file. Rat number is given by the last 3 characters of **SUBJECTS_RatUniqueID**
- Column 28-30 contains the rat number in the study (here, 029)
- Column 32-34 contains the rat number in the study (here, 050)
- Column 36-38 contains the rat number in the study (here, 020)

- Column 40-42 contains the rat number in the study (here, 052)
- File extension .mpg indicates that this is a video file and is a recording of the trial.

LOCOMOTION XY TRACK FILES (CSV)

The name for a track file was a concatenation of several elements and extension “csv” as described below, using for illustration the track file name “Q30DRCq001_01_1_0023_0008736_TrackFile.csv”:

- Column 1-10 contains the value for **SUBJECTS_RatUniqueID** (here, Q30DRCq001), a unique 10 characters long ID for every individual rat tested in studies Q17 to Q48. **SUBJECTS_RatUniqueID** is composed of **QSTUDYinfo_3characterCode**+**QSTUDYinfo_Expt4characterCode**+“rat number in the study”. Values for “rat number in the study” ranged from 001 to the number of rats that entered a given study
- Column 11 is the underscore character (_) to mark the separation from the next code
- Column 12-13 shows the injection # given by **TRIAL_InjectionNumber_str** and this value (here, 01) indicates that it was injection number 1 (number format is 2 characters string)
- Column 14 is the underscore character (_) to mark the separation from the next code
- Column 15 contains a one character code for the dose of the first drug in **TREATMENT_DrugRx_SubstancesAndDosesInjected** (here, 1 indicates 0.125 mg/kg)
- Column 16 is the underscore character (_) to mark the separation from the next code
- Column 17-20 contains a 4 character number assigned in EthoVision work file to the track (.trk) record of the trial (here, 0023)
- Column 21 is the underscore character (_) to mark the separation from the next code
- Column 22-28 contains a 7 characters unique ID number (here, 0008736) assigned to each trial in the SPSS database maintained for studies Q17 to Q48; annotation name of this metadata variable is **ID7STRING**.
- The ending of the file name “_TrackFile.csv” indicates that the file (here, Q30DRCq001_01_1_0023_0008736_TrackFile.csv) contains in tabular format the time series of XY coordinates of the trajectories of locomotion on the open field, as extracted from the video-recording of the trial by EthoVision software.

Altogether, columns 1-28 constitute **TRIAL_ID**, a unique ID for each TRIAL in studies Q17 to Q48 (here, Q30DRCq001_01_1_0023_0008736). Hence, the convention for naming track files is the concatenation of **TRIAL_ID** and "_TrackFile.csv".

PATH PLOT OF ANIMAL TRAJECTORY FILES (GIF)

The convention for naming path plot files is the concatenation of **TRIAL_ID** and "_PathPlot.gif". Corresponding to the above illustration for track file "Q30DRCq001_01_1_0023_0008736_TrackFile.csv", the path plot file is named "Q30DRCq001_01_1_0023_0008736_PathPlot.gif".

DEPOSITS IN GIGADB AND FRDR REPOSITORIES

This Data Note describes a schema to annotate raw data objects from research employing videography and in particular the raw data objects collected in the principal investigator's laboratory during the course of research on an animal model of OCD. Furthermore, with this Data Note, we deposit in the GigaDB repository the metadata annotations for these raw data objects and provide URL links to the actual raw data objects, which are hosted in the FRDR repository (described below).

As explained below, the raw data objects are grouped into different datasets in GigaDB and FRDR repositories. Moreover, the style of metadata annotation is different in GigaDB and FRDR repositories.

DESCRIPTION OF ARCHIVE IN FRDR REPOSITORY

The library of data objects from the animal model of OCD research was deposited as a collection of 29 datasets in the Canadian research data repository, Federated Research Data Repository (FRDR), as a Special Collection entitled *A Digital Library of Behavioural Performance in Standardized Conditions - Szechtman Lab Collection* [67]. Datasets were deposited one by one from 21-Jun-2021 to 6-Aug-2021. Key organizational highlights of FRDR deposits are as follows.

Each dataset in the FRDR repository is of a study assigned a unique 3 character code, Q##. Altogether, during the course of research using the animal model of OCD, 29 such codes were assigned; ranging in value from Q17 to Q48 (there is no study Q19, Q20 or Q32). These 29 studies from Q17 to Q48 constitute the 29 datasets in FRDR repository.

Each dataset in the FRDR repository has a unique title, DOI, authors, and metadata. As seen in Table 1, FRDR dataset titles are a concatenation of 4 elements, delimited by space:

- Project #, formatted as “Project_##” (## is a 2 character string)
- Q##, where ## is a value from 17 to 48
- StudyTitle, a literal string
- Title of study Q##.

Although all dataset titles refer to only one project and one study, some datasets have data objects from more than one experiment. Indeed, across the 29 datasets there are 43 experiments (see Table 1).

The directory structure for every FRDR dataset in the Szechtman Lab Collection is the same: The name of the parent folder is Q17 to Q48. There are four subfolders, each with one type of RAWDATA objects:

- Directory \Q##\03_Videos_mpgFiles\, contains RAWDATA video files
- Directory \Q##\05_EthoVision_csvTrackFiles\, contains RAWDATA track files
- Directory \Q##\06_Pathplots_individual\, contains RAWDATA path plot files
- Directory \Q##\04_EthoVision_backupFiles\, contains a backup of the RAWDATA EthoVision work file

FRDR datasets may have additional subfolders, with different types of files; for example, a montage for each individual rat of path plot images during the course of repeated injections, abstracts and poster presentations from the study, or copies of SPSS files with processed locomotion and checking data from the study.

The landing page for the Szechtman Lab Collection [67] lists the titles of the 29 datasets, and provides ready access to the landing page for each dataset. The dataset landing page shows metadata annotations, including Description of the dataset and Keywords for the dataset. However, the landing page does not expose the annotations for each raw data object in the dataset.

Raw data objects in each dataset were annotated with Hierarchical Keywords (HK), using classes of hierarchy that emulated the information in the Methods section of a research report and thereby applied to all datasets. Those HK are annotated in the meta field <dc:subject>, and mirrored in the meta field <lr:hierarchicalSubject>. HK are stored in xmp sidecar files in the appropriate subdirectory for video (mpg), track (csv), and EthoVision (ewb) files; they are embedded in the metadata fields of the path plot gif files.

Because HR annotations are contained in separate xmp files, there is no simple way to search across all FRDR datasets for raw data objects with specific parameters.

There are 4 characteristics that distinguish the deposits in FRDR and GigaDB repositories:

First, rather than 29 datasets in the FRDR repository, there are in the GigaDB repository 43 datasets, each with one experiment.

Second, metadata for only 3 of 4 raw data objects are deposited in the GigaDB repository. EthoVision backup files are not included because an open source reader for those files is not available and pertinent information from the work files is already contained in metadata annotations. Similarly, non-raw data files in FRDR are not included in the GigaDB repository because they do not contribute to the objective of a large dataset containing performance in a standard arena.

Third, by virtue of their presence in tabular format on the GigaScience and GigaDB websites, annotations for raw data objects within all datasets are clearly exposed and searchable, enhancing the perspective of a library with a single large dataset.

Finally, the GigaDB deposits use a cleaner and richer set of metadata variables to describe the raw data objects. The metadata variables are a more elegant set compared to HK annotations on FRDR because here we improved the naming of metadata variables so that variable names more readily identify their content. Furthermore, compared to FRDR, the GigaDB deposits use a larger set of metadata variables because the structure of a GigaDB database requires to specify several attributes of a record that was not annotated for the raw data objects in the FRDR repository; for example, video resolution, video recording equipment, taxonomic id of subjects, supplier from where the rats were purchased (these attributes were not annotated as HK but were specified in the readme.txt file for the dataset deposited in the FRDR repository).

RE-USE POTENTIAL

Two characteristics give the library high re-use potential: its sheer size and the annotation schema. Large size provides sufficient data for machine learning techniques in analysis of spontaneous behavior. For example, the library could be used to train neural networks to track body parts and extract postural kinematics; to identify and measure ritual behavior of relevance to OCD (along the lines scored manually in [27]); or to classify effects of different drugs on behavioral activity.

Similarly, large dataset makes feasible statistical evaluation of the effects of subtle factors, such as circadian, seasonal or annual rhythms or in the detection of drifts in dependent measures across years. Moreover, the library permits to quantify across trials the range of possible responses, across individuals the range of individual differences, and across time the change with repeated testing.

Lastly, the many tests in the library from a single paradigm provide an educational resource, for instance, for student research projects.

Re-use potential is further increased by the annotation schema, which effectively created a large pool of independent events with a variety of well specified parameters. This permits the simulation of new experiments through selection of particular parameters. As such, the library has the potential to test different hypotheses, and to make novel discoveries.

The library may have yet another utility, a less obvious one but potentially impactful. As raised in the Introduction, the underlying motivation in making the raw data objects publically available is to ultimately create a machine searchable virtual library of behavioral performance in defined conditions. The approach used here in annotations of the raw data objects is a step towards this goal. However, to achieve the goal of searchable linked open datasets requires a more sophisticated and formal schema [68-70]. The library described here could be of value both as the seed and test bed in this endeavor.

LIMITATIONS ON RE-USE POTENTIAL

At first blush the above assertion for re-use potential seems exaggerated for two reasons. First, video resolution is low (352 x 240 pixels), which may be insufficient for currently employed sophisticated methods of behavior analysis. Second, the effective size of the library is much smaller given differences in drug treatments, brain treatments, testing arenas, and number of tests rats received. We consider these points in turn.

VIDEO RESOLUTION

Our video recordings are a top-down view of a large open field (1.6 x 1.6 m) and of a (relatively small) rat within it. Clearly, the 352x240 pixel image size is inadequate to analyze small movements of body parts such as whiskers (e.g., [71]) but this resolution is more than adequate to track whole body locomotion. However, importantly, is that image size good enough to resolve kinematics and body postures tracked by several modern video analysis systems cited in the Introduction? This is an empirical question. Even though those video analysis systems were likely developed using higher resolution images, nevertheless their method could work on a lower resolution video or be adapted to work on it. This is not unlike the situation for the methods in molecular biology which were originally developed to analyze living tissue under optimum conditions but which can be employed now to examine archeological samples.

However, should video resolution indeed be a problem, perhaps such a limitation could be remedied with software to enhance and upscale videos (e.g., Topaz Video Enhance AI; [72]), an option which an interested user could explore.

OVERALL AND EFFECTIVE SIZE OF LIBRARY

The 19,976 records in the library are not a homogenous sample, with only one set of independent variables. Rather, the library is composed of tests with a range of independent variables specified by the metadata annotations. Hence, the effective size of the library for analysis depends on the question of interest. All records can be used if the question of interest is about the range of possible values. However, if the question of interest is about the range of values under a specific condition then clearly the effective size of the library for this analysis is smaller, equivalent to the number of trials in the library with the specified parameters of the independent variables. It follows that for some questions, the number of available trials may be inadequate or nil. For example, if the focus were on performance of rats injected specifically with CP809101 then only 6 such trials would be available for analysis (Table 4); moreover, if the question were narrowed down further to performance of these rats in the standard arena, then no trials would be available for analysis since rats injected with CP809101 were tested only in the arena with a small Plexiglas fence. In contrast, if the question of interest were on treatment with saline, then the effective size of 7551 records would be large (Table 4) but would be smaller by specifying additional constraints.

The size of a subgroup with any combination of parameters of the independent variables can be obtained by consulting Supplementary Material files and filtering the appropriate table by the parameters of interest. This can be accomplished relatively readily using worksheet "GigaDB_MetaDataTable_20220421" in file *GigaDB_MetaDataTable_20220421_SupplementaryMaterial.xlsx* and opening a variable in the header row and selecting the parameters of interest as filter. After filters are applied, the number of available track, path plot and video files is given in the bottom row labelled **Total,** in fields (variables) named **RAWDATA_TrackFileForTrial_AvailableYESorNO** (column AT), **RAWDATA_TrackPathplotForTrial_AvailableYESorNO** (column AX), and **RAWDATA_VideoFileForTrial_AvailableYESorNO** (column BA) respectively.

DATA AVAILABILITY

The described raw data objects and their metadata are available from the FRDR repository [67], where the data were first deposited in 2021 as 29 datasets [73-101]. The 29 datasets were packaged based on the code Q17 to Q48 assigned to successive studies during the course of research using the animal model of OCD.

Metadata for the same described raw data objects are also available in the GigaDB repository, where the data are packaged as 43 datasets [102-144]. This number of datasets corresponds to the number of experiments in the library. The data are packaged as one experiment per

dataset in the GigaDB repository because this provides a better fit between dataset titles and the grouping of datasets into projects (Table 1).

Metadata in the GigaDB repository are annotated in a simpler style than in FRDR. Annotations in GigaDB are visible on the dataset landing page as tabular data and can be searched readily. In contrast, in the FRDR repository, each raw data object has a separate annotation file within the dataset, and those metadata variables are not visible on the dataset landing page.

SUPPLEMENTARY MATERIAL

Included with this Data Note publication are three supplementary files:

TABLE OF ANNOTATIONS FOR ALL 43 DATASETS IN GIGADB REPOSITORY

GigaDB_SampleAndFilesTabs_20220420_SupplementaryMaterial.xlsx is an Excel workbook file with the annotations for the 43 datasets in the GigaDB repository, combined as one large table. There is one worksheet for the Sample tab (named, "GigaDB_SampleTab_20220420") and another worksheet for the Files tab ("GigaDB_FilesTab_20220420"). The two tables are sorted by **QSTUDYinfo_ExptNumberInProject**, **SUBJECTS_RatUniqueID**, **TRIAL_InjectionNumber_str**; in addition, the Files tab table is sorted (in descending sort order) by "Data_Type". This sorting gives across the 43 datasets a seamless view of the files (raw data objects) in the Files tab associated with the particular trial (Sample_ID) in the Sample tab. However, there are extra columns in the Files tab worksheet (columns L to R), copied from the Sample tab, which would permit to sort the tables in any other order, consistently across the Sample and Files tabs.

Note that the variables "File_Size" and "Release_Date" are empty and there are no columns with DOIs and URLs of the GigaDB datasets or files; the Excel workbook will need an update once those are assigned.

A CRUDE INTERFACE TO DATA OBJECTS IN FRDR REPOSITORY

FRDR_interface_20211029_SupplementaryMaterial.xlsx is an Excel workbook file with a rudimentary interface to the datasets and raw data objects in the FRDR repository. The source for the interface is a master table (named, tblMaster) in the "MetaDataTable" worksheet. This source master table is essentially a flat database of variables used in the construction of the Hierarchical Keywords with which the data objects were annotated. The table includes also the URL links to the data objects as well as to the xmp sidecars containing the annotations.

As is apparent from description in the worksheet "Background", the Hierarchical Keywords schema is the same as used for annotations of the GigaDB datasets. However, some of the variable names are not identical due to refinements introduced in GigaDB annotations. A copy

of the variable names and their descriptive labels are listed in the datasheet “VariableLabels” while the datasheet “VariableValueLabels” shows the values and the label of those values for some of the variables.

A rudimentary interface to FRDR datasets and data objects was constructed with Pivot Tables, using tblMaster as the source. Three examples are provided in the worksheets “WhatObjectsAvailable”, “ListingOfDrugRx” and URL_RAWDATA_forDownload” with the last one providing links to data objects with the selected attributes.

SOURCE TABLE FOR A GIGADB INTERFACE

The third file, *GigaDB_MetaDataTable_20220421_SupplementaryMaterial.xlsx*, is also an Excel workbook; it contains in the worksheet “MetaDataTable” a flat database of the variables used for annotation of the raw data objects in the GigaDB repository. The table is a duplicate of the tables in the Sample and Files tabs in the first supplementary file, *GigaDB_SampleAndFilesTabs_20220420_SupplementaryMaterial.xlsx*. It differs in the layout but not in the information contained in *GigaDB_SampleAndFilesTabs_20220420_SupplementaryMaterial.xlsx* file. Being a flat database, the table has only one record per trial (identified by the two redundant variables **ID7STRING** and **TRIAL_ID**), with the record containing all the attribute variables of the trial (“sample”) in separate columns or fields. Thus, rather than the layout in the Files tab, where each data object is on a separate record, attribute variables for different data files are all aligned on the **ID7STRING** record in separate fields. Necessarily, there is a unique variable name for each type of data objects; for example, there are three variables that hold the name of the file with data objects, **RAWDATA_VideoFileForTrial_FileName**, **RAWDATA_TrackFileForTrial_FileName**, **RAWDATA_TrackPathplotForTrial_FileName** (corresponding to **RAWDATA_FileName** in Files tab).

Like for the source table in the *FRDR_interface_20211029_SupplementaryMaterial.xlsx* workbook, Pivot Tables can use the current table to search the GigaDB datasets. One example is in the worksheet “ListingOfDrugRx”, which contains a Pivot Table similar to the one in the *FRDR_interface_20211029_SupplementaryMaterial.xlsx* workbook.

More importantly, the source table is provided to facilitate the efforts of building a platform towards a machine searchable virtual library of behavioral performance in defined conditions.

ABBREVIATIONS

BLA: Basal Lateral Amygdala

CP809101: (2-(3-chlorobenzyloxy)-6-(piperazin-1-yl)pyrazine), agonist of serotonin 2C receptors

DPAT: 8OHDPAT, 8-Hydroxy-N,N-dipropyl-2-aminotetralin, agonist of serotonin 1A receptors

DPAT+ES: 8OHDPAT co-administered with escitalopram, selective serotonin reuptake inhibitor

ILC: Infralimbic Cortex

NAc: Nucleus Accumbens Core

NMDA: N-methyl-D-aspartate, an excitotoxin

OFC: Orbital Frontal Cortex

PBS: phosphate-buffered saline

QNP: Quinpirole hydrochloride, (4aR,8aR)-5-propyl-4,4a,5,6,7,8,8a,9-octahydro-1H-pyrazolo[3,4-g]quinoline, agonist of dopamine D2/D3 receptors

QNP+IM: QNP co-administered with imipramine, a tricyclic antidepressant

QNP+clorgyline: QNP co-administered with clorgyline, a selective inhibitor of monoamine oxidase A

ROI: region of interest

SAL: 0.9% saline solution

U69593: N-methyl-2-phenyl-N-[(5R,7S,8S)-7-(pyrrolidin-1-yl)-1-oxaspiro[4.5]dec-8-yl]acetamide, agonist of opioid kappa receptors

mCPP: 1-(3-Chlorophenyl)-piperazine hydrochloride, agonist of serotonin receptors

mCPP+ritanserin: mCPP co-administered with ritanserin, antagonist of serotonin 2A/C receptors

vcr: video-cassette recorder

COMPETING INTERESTS

Authors declare that they have no competing interests.

AUTHORS' CONTRIBUTIONS

HS – principal investigator on all studies, maintained the SPSS flat database for all studies, consolidated SPSS data records and data object files into a consistent framework, developed

the annotation schema and annotated the datasets, wrote first draft of manuscript, contributed to and approved final version of manuscript.

ADG – responsible for data processing and analysis in all studies, developed procedures and software scripts used in data processing and in analysis of compulsive checking, was the lead investigator and author on several publications from studies on compulsive checking, contributed to design of the FRDR collection and to the annotation schema, worked on writing the manuscript and approved the final version.

AGM – provided input on submission of the library to a public repository, contributed to design of the annotation schema and to the writing of the report.

ACKNOWLEDGEMENTS

The endeavor to deposit in a public repository the data objects from studies using the animal model of OCD was initiated upon the encouragement of Natalia Bielczyk who appraised its value and who made introductory inquiries to *GigaScience* regarding potential interest; many thanks to Natalia for this. I am grateful to Isaac Pratt, Research Data Management Specialist at McMaster University Library who went over the options available, suggested the FRDR as a suitable repository and introduced the project to Erin Clary, Curation Coordinator at Portage Network. Erin was most generous with her time and guidance - she was instrumental in arranging for a Special Collection to house the archive and provided tireless help and encouragement throughout the entire process of making the library come to fruition; many thanks to Erin. Chris Amit of *GigaScience* provided detailed comments and feedback to our inquiries and made the invaluable suggestion to re-organize the datasets into their present, more elegant form.

Data in the library is the direct product of the work by many individuals, including: graduate students—Kirsten Culver, Melissa Perreault, Laura B Ray, Liane Bisnaire, Charmaine Silva, Thomas J.A. McMurran, and Mark C. Tucci—who addressed mechanisms of OCD and sensitization during the course of their thesis research; research staff, Donna Waxman and Dawn Graham, who managed the daily workflow in the lab; Boris Sakic, who was in charge of the lab and was instrumental in making the research happen in 2015-2016; undergraduate students, who worked in the lab during the summer months or carried out their research for undergraduate thesis projects; and Javier Ballester González, who undertook research training in the lab as part of a fellowship from Fundacion Espanola de Psiquiatria y Salud Mental, Madrid, Spain.

Research using the animal model of OCD was supported by grants to HS from the Canadian Institutes of Health Research (CIHR) operating grants MOP-64424 and MT-12852, Ontario

Mental Health Foundation (OMHF) Type A grant “Psychobiology of security motivation in an animal model of OCD”, and Natural Sciences and Engineering Research Council of Canada (NSERC) operating grant RGPIN A0544.

REFERENCES

1. Teitelbaum P, Szechtman H, Sirkin DW and Golani I. Dimensions of movement, movement subsystems and local reflexes in the dopaminergic systems underlying exploratory locomotion. In: Spiegelstein MY and Levy A, editors. Behavioral Models and the Analysis of Drug Action. Amsterdam: Elsevier Scientific Publishing Company; 1982. p. 357-85.

2. Szechtman H, Eilam D, Teitelbaum P and Golani I. A different look at measurement and interpretation of drug-induced stereotyped behavior. Psychobiology. 1988;16 2:164-73.

3. Teitelbaum P and Pellis SM. Toward a synthetic physiological psychology. Psychol Sci. 1992;3 1:4-20. doi:10.1111/j.1467-9280.1992.tb00249.x.

4. Golani I. A mobility gradient in the organization of vertebrate movement - the perception of movement through symbolic language. Behav Brain Sci. 1992;15 2:249-66. doi:10.1017/S0140525X00068539.

5. Gomez-Marin A, Paton JJ, Kampff AR, Costa RM and Mainen ZF. Big behavioral data: psychology, ethology and the foundations of neuroscience. Nat Neurosci. 2014;17 11:1455-62. doi:10.1038/nn.3812.

6. Berman GJ. Measuring behavior across scales. BMC Biol. 2018;16 1:23. doi:10.1186/s12915-018-0494-7.

7. White SR, Amarante LM, Kravitz AV and Laubach M. The Future Is Open: Open-Source Tools for Behavioral Neuroscience Research. eNeuro. 2019;6 4 doi:10.1523/ENEURO.0223-19.2019.

8. OpenBehavior Project: About OpenBehavior - OpenBehavior. https://edspace.american.edu/openbehavior/. Accessed 11 Apr 2022.

9. Noldus LP, Trienes RJ, Hendriksen AH, Jansen H and Jansen RG. The Observer Video-Pro: new software for the collection, management, and presentation of time-structured data from videotapes and digital media files. BehavResMethods InstrumComput. 2000;32 1:197-206.

10. Ben-Shaul Y. OptiMouse: a comprehensive open source program for reliable detection and analysis of mouse body and nose positions. BMC Biol. 2017;15 1:41. doi:10.1186/s12915-017-0377-3.

11. Franco-Restrepo JE, Forero DA and Vargas RA. A Review of Freely Available, Open-Source Software for the Automated Analysis of the Behavior of Adult Zebrafish. Zebrafish. 2019;16 3:223-32. doi:10.1089/zeb.2018.1662.

12. Noldus LP, Spink AJ and Tegelenbosch RA. EthoVision: a versatile video tracking system for automation of behavioral experiments. Behav Res Methods Instrum Comput. 2001;33 3:398-414.

13. Clever Sys: Automated Animal Behavior Analysis – Software, Hardware, and Services. http://cleversysinc.com/CleverSysInc/. Accessed 18 Jul 2022.

14. Wiltschko AB, Johnson MJ, Iurilli G, Peterson RE, Katon JM, Pashkovski SL, et al. Mapping Sub-Second Structure in Mouse Behavior. Neuron. 2015;88 6:1121-35. doi:10.1016/j.neuron.2015.11.031.

15. Mathis A, Mamidanna P, Cury KM, Abe T, Murthy VN, Mathis MW, et al. DeepLabCut: markerless pose estimation of user-defined body parts with deep learning. Nat Neurosci. 2018; doi:10.1038/s41593-018-0209-y.

16. de Chaumont F, Ey E, Torquet N, Lagache T, Dallongeville S, Imbert A, et al. Real-time analysis of the behaviour of groups of mice via a depth-sensing camera and machine learning. Nat Biomed Eng. 2019;3 11:930-42. doi:10.1038/s41551-019-0396-1.

17. Krynitsky J, Legaria AA, Pai JJ, Garmendia-Cedillos M, Salem G, Pohida T, et al. Rodent Arena Tracker (RAT): A Machine Vision Rodent Tracking Camera and Closed Loop Control System. eNeuro. 2020;7 3 doi:10.1523/ENEURO.0485-19.2020.

18. Geuther BQ, Peer A, He H, Sabnis G, Philip VM and Kumar V. Action detection using a neural network elucidates the genetics of mouse grooming behavior. Elife. 2021;10 doi:10.7554/eLife.63207.

19. Klibaite U, Kislin M, Verpeut JL, Sun X, Shaevitz JW and Wang SSH. Deep Behavioral Phenotyping of Mouse Autism Models using Open-Field Behavior. bioRxiv. 2021:2021.02.16.431500. doi:10.1101/2021.02.16.431500.

20. Marshall JD, Aldarondo DE, Dunn TW, Wang WL, Berman GJ and Olveczky BP. Continuous Whole-Body 3D Kinematic Recordings across the Rodent Behavioral Repertoire. Neuron. 2021;109 3:420-37 e8. doi:10.1016/j.neuron.2020.11.016.

21. Sun G, Lyu C, Cai R, Yu C, Sun H, Schriver KE, et al. DeepBhvTracking: A Novel Behavior Tracking Method for Laboratory Animals Based on Deep Learning. Front Behav Neurosci. 2021;15:750894. doi:10.3389/fnbeh.2021.750894.

22. Lauer J, Zhou M, Ye S, Menegas W, Schneider S, Nath T, et al. Multi-animal pose estimation, identification and tracking with DeepLabCut. Nat Methods. 2022;19 4:496-504. doi:10.1038/s41592-022-01443-0.

23. Qualman SJ, France M, Grizzle WE, LiVolsi VA, Moskaluk CA, Ramirez NC, et al. Establishing a tumour bank: banking, informatics and ethics. Br J Cancer. 2004;90 6:1115-9. doi:10.1038/sj.bjc.6601678.

24. Schrohl AS, Wurtz S, Kohn E, Banks RE, Nielsen HJ, Sweep FC, et al. Banking of biological fluids for studies of disease-associated protein biomarkers. Mol Cell Proteomics. 2008;7 10:2061-6. doi:10.1074/mcp.R800010-MCP200.

25. Szechtman H, Sulis W and Eilam D. Quinpirole induces compulsive checking behavior in rats: a potential animal model of obsessive-compulsive disorder (OCD). Behav Neurosci. 1998;112 6:1475-85. doi:10.1037//0735-7044.112.6.1475.

26. Wikipedia contributors. Quinpirole. Wikipedia, The Free Encyclopedia. 2021. https://en.wikipedia.org/w/index.php?title=Quinpirole&oldid=1023855664.

27. Ben-Pazi A, Szechtman H and Eilam D. The morphogenesis of motor rituals in rats treated chronically with the dopamine agonist quinpirole. Behav Neurosci. 2001;115 6:1301-17. doi:10.1037//0735-7044.115.6.1301.

28. Szechtman H, Eckert MJ, Tse WS, Boersma JT, Bonura CA, McClelland JZ, et al. Compulsive checking behavior of quinpirole-sensitized rats as an animal model of Obsessive-Compulsive Disorder(OCD): form and control. BMC Neurosci. 2001;2:4. doi:10.1186/1471-2202-2-4.

29. Dvorkin A, Perreault ML and Szechtman H. Development and temporal organization of compulsive checking induced by repeated injections of the dopamine agonist quinpirole in an animal model of obsessive-compulsive disorder. Behav Brain Res. 2006;169 2:303-11. doi:10.1016/j.bbr.2006.01.024.

30. Zadicario P, Ronen S and Eilam D. Modulation of quinpirole-induced compulsive-like behavior in rats by environmental changes: implications for OCD rituals and for exploration and navigation. BMC Neurosci. 2007;8:23. doi:10.1186/1471-2202-8-23.

31. Szechtman H, Culver K and Eilam D. Role of dopamine systems in obsessive-compulsive disorder (OCD): implications from a novel psychostimulant-induced animal model. Pol J Pharmacol. 1999;51 1:55-61.

32. Man J, Hudson AL, Ashton D and Nutt DJ. Animal models for obsessive-compulsive disorder. Curr Neuropharmacol. 2004;2 2:169-81.

33. Eilam D and Szechtman H. Psychostimulant-induced behavior as an animal model of obsessive-compulsive disorder: An ethologlocal approach to the form of compulsive rituals. CNS Spectrums. 2005;10 3:191-202. doi:10.1017/S109285290001004X.

34. Szechtman H and Eilam D. Psychiatric models. In: Whishaw IQ and Kolb B, editors. The Behavior of the Laboratory Rat: A Handbook With Tests. New York: Oxford University Press; 2005. p. 462-74.

35. Korff S and Harvey BH. Animal models of obsessive-compulsive disorder: rationale to understanding psychobiology and pharmacology. Psychiatr Clin North Am. 2006;29 2:371-90. doi:10.1016/j.psc.2006.02.007.

36. Westenberg HG, Fineberg NA and Denys D. Neurobiology of obsessive-compulsive disorder: serotonin and beyond. CNS Spectrums. 2007;12 2 Suppl 3:14-27.

37. Hoffman KL. Animal models of obsessive compulsive disorder: recent findings and future directions. Expert opinion on drug discovery. 2011;6 7:725-37. doi:10.1517/17460441.2011.577772.

38. Albelda N and Joel D. Current animal models of obsessive compulsive disorder: an update. Neuroscience. 2012;211 0:83-106. doi:10.1016/j.neuroscience.2011.08.070.

39. Ahmari SE and Dougherty DD. Dissecting OCD circuits: from animal models to targeted treatments. Depress Anxiety. 2015;32 8:550-62. doi:10.1002/da.22367.

40. Stuchlik A, Radostova D, Hatalova H, Vales K, Nekovarova T, Koprivova J, et al. Validity of Quinpirole Sensitization Rat Model of OCD: Linking Evidence from Animal and Clinical Studies. Front Behav Neurosci. 2016;10:209. doi:10.3389/fnbeh.2016.00209.

41. Szechtman H, Ahmari SE, Beninger RJ, Eilam D, Harvey BH, Edemann-Callesen H, et al. Obsessive-compulsive disorder: Insights from animal models. Neurosci Biobehav Rev. 2017;76 Pt B:254-79. doi:10.1016/j.neubiorev.2016.04.019.

42. Eilam D and Szechtman H. Dosing regimen differentiates sensitization of locomotion and mouthing to D2 agonist quinpirole. Pharmacol Biochem Behav. 1990;36 4:989-91. doi:10.1016/0091-3057(90)90111-t.

43. Einat H and Szechtman H. Environmental modulation of both locomotor response and locomotor sensitization to the dopamine agonist quinpirole. Behav Pharmacol. 1993;4 4:399-403.

44. Szechtman H, Talangbayan H and Eilam D. Environmental and behavioral components of sensitization induced by the dopamine agonist quinpirole. Behav Pharmacol. 1993;4 4:405-10.

45. Szechtman H, Dai H, Mustafa S, Einat H and Sullivan RM. Effects of dose and interdose interval on locomotor sensitization to the dopamine agonist quinpirole. Pharmacol Biochem Behav. 1994;48 4:921-8. doi:10.1016/0091-3057(94)90201-1.

46. Szechtman H, Talangbayan H, Canaran G, Dai H and Eilam D. Dynamics of behavioral sensitization induced by the dopamine agonist quinpirole and a proposed central energy control mechanism. Psychopharmacology (Berl). 1994;115 1-2:95-104. doi:10.1007/BF02244757.

47. Einat H, Einat D, Allan M, Talangbayan H, Tsafnat T and Szechtman H. Associational and nonassociational mechanisms in locomotor sensitization to the dopamine agonist quinpirole. Psychopharmacology (Berl). 1996;127 2:95-101. doi:10.1007/BF02805980.

48. Szumlinski KK, Allan M, Talangbayan H, Tracey A and Szechtman H. Locomotor sensitization to quinpirole: environment-modulated increase in efficacy and context-dependent increase in potency. Psychopharmacology (Berl). 1997;134 2:193-200. doi:10.1007/s002130050442.

49. Dvorkin A, Culver KE and Szechtman H. Differential effects of clorgyline on sensitization to quinpirole in rats tested in small and large environments. Psychopharmacology (Berl). 2006;186 4:534-43. doi:10.1007/s00213-006-0377-4.

50. Perreault ML, Seeman P and Szechtman H. Kappa-opioid receptor stimulation quickens pathogenesis of compulsive checking in the quinpirole sensitization model of obsessive-compulsive disorder (OCD). Behav Neurosci. 2007;121 5:976-91. doi:10.1037/0735-7044.121.5.976.

51. Dvorkin A, Culver KE, Waxman D, Szechtman H and Kolb B. Effects of hypophysectomy on compulsive checking and cortical dendrites in an animal model of obsessive-compulsive disorder. Behav Pharmacol. 2008;19 4:271-83. doi:10.1097/FBP.0b013e3283095223.

52. Dvorkin A, Silva C, McMurran T, Bisnaire L, Foster J and Szechtman H. Features of compulsive checking behavior mediated by nucleus accumbens and orbital frontal cortex. Eur J Neurosci. 2010;32 9:1552-63. doi:10.1111/j.1460-9568.2010.07398.x.

53. Alkhatib AH, Dvorkin-Gheva A and Szechtman H. Quinpirole and 8-OH-DPAT induce compulsive checking behavior in male rats by acting on different functional parts of an OCD neurocircuit. Behav Pharmacol. 2013;24 1:65-73. doi:10.1097/FBP.0b013e32835d5b7a.

54. Tucci MC, Dvorkin-Gheva A, Graham D, Amodeo S, Cheon P, Kirk A, et al. Effects of the serotonergic agonist mCPP on male rats in the quinpirole sensitization model of obsessive-compulsive disorder (OCD). Psychopharmacology (Berl). 2013;227 2:277-85. doi:10.1007/s00213-013-2976-1.

55. Tucci MC, Dvorkin-Gheva A, Johnson E, Cheon P, Taji L, Agarwal A, et al. Performance of compulsive behavior in rats is not a unitary phenomenon - validation of separate functional components in compulsive checking behavior. Eur J Neurosci. 2014;40 6:2971-9. doi:10.1111/ejn.12652.

56. Tucci MC, Dvorkin-Gheva A, Sharma R, Taji L, Cheon P, Peel J, et al. Separate mechanisms for development and performance of compulsive checking in the quinpirole sensitization rat model of obsessive-compulsive disorder (OCD). Psychopharmacology (Berl). 2014;231 18:3707-18. doi:10.1007/s00213-014-3505-6.

57. Ballester Gonzalez J, Dvorkin-Gheva A, Silva C, Foster JA and Szechtman H. Nucleus accumbens core and pathogenesis of compulsive checking. Behav Pharmacol. 2015;26 1-2:200-16. doi:10.1097/FBP.0000000000000112.

58. Tucci MC, Dvorkin-Gheva A, Johnson E, Wong M and Szechtman H. 5-HT2A/C receptors do not mediate the attenuation of compulsive checking by mCPP in the quinpirole sensitization rat model of obsessive-compulsive disorder (OCD). Behav Brain Res. 2015;279:211-7. doi:10.1016/j.bbr.2014.11.017.

59. Jung TD, Jung PS, Raveendran L, Farbod Y, Dvorkin-Gheva A, Sakic B, et al. Changes in gut microbiota during development of compulsive checking and locomotor sensitization induced by chronic treatment with the dopamine agonist quinpirole. Behav Pharmacol. 2018;29 2 and 3-Spec Issue:211-24. doi:10.1097/FBP.0000000000000363.

60. Szechtman H, Harvey BH, Woody EZ and Hoffman KL. The Psychopharmacology of Obsessive-Compulsive Disorder: A Preclinical Roadmap. Pharmacol Rev. 2020;72 1:80-151. doi:10.1124/pr.119.017772.

61. Avni R, Zadicario P and Eilam D. Exploration in a dark open field: a shift from directional to positional progression and a proposed model of acquiring spatial information. Behav Brain Res. 2006;171 2:313-23.

62. Ray L. *The effect of neurotoxic lessons to the infralimbic prefrontal cortex on quinpitole-induced compulsive checking behaviour*. Thesis (M Sc), McMaster University, [Hamilton, Ont.], 2006.

63. Culver KE and Szechtman H. Hypophysectomy does not block sensitization to the dopamine agonist quinpirole or its modulation by the MAOI clorgyline. Horm Behav. 2004;45 1:23-30. doi:10.1016/j.yhbeh.2003.07.004.

64. Spink AJ, Tegelenbosch RA, Buma MO and Noldus LP. The EthoVision video tracking system--a tool for behavioral phenotyping of transgenic mice. Physiol Behav. 2001;73 5:731-44.

65. Wolfram Research Inc. (2005). Mathematica (Version 5.2). https://www.wolfram.com/mathematica/.

66. Hen I, Sakov A, Kafkafi N, Golani I and Benjamini Y. The dynamics of spatial behavior: how can robust smoothing techniques help? J Neurosci Methods. 2004;133 1-2:161-72.

67. Federated Research Data Repository (FRDR): A Digital Library of Behavioural Performance in Standardized Conditions - Szechtman Lab Collection. https://www.frdr-dfdr.ca/repo/collection/szechtmanlab. Accessed 5 Apr 2022.

68. Gkoutos GV, Hoehndorf R, Tsaprouni L and Schofield PN. Best behaviour? Ontologies and the formal description of animal behaviour. Mamm Genome. 2015;26 9-10:540-7. doi:10.1007/s00335-015-9590-y.

69. Larson SD and Martone ME. Ontologies for Neuroscience: What are they and What are they Good for? Front Neurosci. 2009;3 1:60-7. doi:10.3389/neuro.01.007.2009.

70. Jovanovik M and Trajanov D. Consolidating drug data on a global scale using Linked Data. J Biomed Semantics. 2017;8 1:3. doi:10.1186/s13326-016-0111-z.

71. Azarfar A, Zhang Y, Alishbayli A, Miceli S, Kepser L, van der Wielen D, et al. An open-source high-speed infrared videography database to study the principles of active sensing in freely navigating rodents. Gigascience. 2018;7 12 doi:10.1093/gigascience/giy134.

72. Topaz Labs: Video Enhance AI – Video Quality Software. https://www.topazlabs.com/video-enhance-ai. Accessed 25 Jun 2022.

73. Szechtman H, Dvorkin-Gheva A and Ray LB. Project_01 Q22 StudyTitle Probing the neural circuit mediating sensitization and compulsive checking: role of the infralimbic

(IL) cortex on expression. Federated Research Data Repository. 2021. https://doi.org/10.20383/102.0442.

74. Szechtman H, Dvorkin-Gheva A and Bisnaire LD. Project_01 Q25 StudyTitle Probing the neural circuit mediating sensitization and compulsive checking: role of the basal lateral amygdala (BLA) on development, and, on expression. Federated Research Data Repository. 2021. https://doi.org/10.20383/102.0439.

75. Szechtman H, Dvorkin-Gheva A and McMurran TJA. Project_01 Q28 StudyTitle Probing the neural circuit mediating sensitization and compulsive checking: role of the lateral orbitofrontal cortex (OFC) on development, and, on expression. Federated Research Data Repository. 2021. https://doi.org/10.20383/102.0436.

76. Szechtman H, Dvorkin-Gheva A, Silva CA and Ballester Gonzalez J. Project_01 Q26 StudyTitle Probing the neural circuit mediating sensitization and compulsive checking: role of the nucleus accumbens core (NAc) on development, and, on expression. Federated Research Data Repository. 2021. https://doi.org/10.20383/102.0438.

77. Szechtman H, Dvorkin-Gheva A and Perreault ML. Project_02 Q23 StudyTitle Probing the neurochemistry of sensitization and compulsive checking: dopamine and kappa opioid systems. Federated Research Data Repository. 2021. https://doi.org/10.20383/102.0441.

78. Szechtman H and Dvorkin-Gheva A. Project_09 Q29 StudyTitle Probing the environmental modulation of sensitization and compulsive checking: influence of a small ledge around OF. Federated Research Data Repository. 2021. https://doi.org/10.20383/102.0435.

79. Szechtman H and Dvorkin-Gheva A. Project_04 Q33 StudyTitle Probing for drugs that mitigate sensitization and compulsive checking: acute treatment with escitalopram or imipramine. Federated Research Data Repository. 2021. https://doi.org/10.20383/102.0432.

80. Szechtman H, Dvorkin-Gheva A and Alkhatib AH. Project_02 Q34 StudyTitle Probing the neurochemistry of sensitization and compulsive checking: dopamine and serotonin systems. Federated Research Data Repository. 2021. https://doi.org/10.20383/102.0431.

81. Szechtman H and Dvorkin-Gheva A. Project_02 Q45 StudyTitle Probing the neurochemistry of sensitization and compulsive checking: dopamine and serotonin systems. Federated Research Data Repository. 2021. https://doi.org/10.20383/102.0420.

82. Szechtman H and Dvorkin-Gheva A. Project_02 Q46 StudyTitle Probing the neurochemistry of sensitization and compulsive checking: dopamine and serotonin systems. Federated Research Data Repository. 2021. https://doi.org/10.20383/102.0419.

83. Szechtman H and Dvorkin-Gheva A. Project_02 Q47 StudyTitle Probing the neurochemistry of sensitization and compulsive checking: dopamine and serotonin systems. Federated Research Data Repository. 2021. https://doi.org/10.20383/102.0418.

84. Szechtman H and Dvorkin-Gheva A. Project_03 Q30 StudyTitle Pharmacology of sensitization and compulsive checking: dose-response profile of quinpirole (0 0.0312 0.0625 0.125 0.25 and 0.5 mg/kg). Federated Research Data Repository. 2021. https://doi.org/10.20383/102.0434.

85. Szechtman H, Dvorkin-Gheva A and Alkhatib AH. Project_03 Q35 StudyTitle Pharmacology of sensitization and compulsive checking: dose-response profile of 8OHDPAT (0 0.03 0.125 0.25 and 1 mg/kg). Federated Research Data Repository. 2021. https://doi.org/10.20383/102.0430.

86. Szechtman H, Dvorkin-Gheva A and Tucci MC. Project_03 Q36 StudyTitle Pharmacology of sensitization and compulsive checking: dose-response profile of mCPP (0 0.3125 0.625 or 1.25 mg/kg). Federated Research Data Repository. 2021. https://doi.org/10.20383/102.0429.

87. Szechtman H, Dvorkin-Gheva A and Tucci MC. Project_04 Q37 StudyTitle Probing for drugs that mitigate sensitization and compulsive checking: effect of mCPP dose (0 0.625 and 1.25 mg/kg) on development produced by a low dose of QNP (0.125 mg/kg). Federated Research Data Repository. 2021. https://doi.org/10.20383/102.0428.

88. Szechtman H, Dvorkin-Gheva A and Tucci MC. Project_04 Q38 StudyTitle Probing for drugs that mitigate sensitization and compulsive checking: effect of mCPP dose (0 0.625 and 1.25 mg/kg) on development produced by a medium dose of QNP (0.25 mg/kg). Federated Research Data Repository. 2021. https://doi.org/10.20383/102.0427.

89. Szechtman H, Dvorkin-Gheva A and Tucci MC. Project_04 Q39 StudyTitle Probing for drugs that mitigate sensitization and compulsive checking: effect of mCPP dose (0 0.3125 0.625 and 1.25 mg/kg) on expression induced by a low dose of QNP (0.125 mg/kg). Federated Research Data Repository. 2021. https://doi.org/10.20383/102.0426.

90. Szechtman H, Dvorkin-Gheva A and Tucci MC. Project_05 Q44 StudyTitle Probing the mode of action of mCPP on compulsive checking: 5-HT2A/C receptors. Federated Research Data Repository. 2021. https://doi.org/10.20383/102.0421.

91. Szechtman H, Dvorkin-Gheva A, Culver KE and Waxman D. Project_06 Q17 StudyTitle Probing the mode of action of quinpirole on behavior: role of the monoamine oxidase inhibitor (MAOI) sensitive quinpirole binding site (MQB). Federated Research Data Repository. 2021. https://doi.org/10.20383/102.0445.

92. Szechtman H, Dvorkin-Gheva A, Culver KE and Waxman D. Project_07 Q18 StudyTitle Probing the role of hormones in compulsive checking: Is the endocrine system necessary for compulsive behavior? Federated Research Data Repository. 2021. https://doi.org/10.20383/102.0444.

93. Szechtman H, Dvorkin-Gheva A and Perreault ML. Project_08 Q21 StudyTitle Probing the parameters of sensitization and compulsive checking: characteristics of behavioral measures during pathogenesis of compulsive behavior. Federated Research Data Repository. 2021. https://doi.org/10.20383/102.0443.

94. Szechtman H and Dvorkin-Gheva A. Project_09 Q24 StudyTitle Probing the environmental modulation of sensitization and compulsive checking: effects of a walled circular arena. Federated Research Data Repository. 2021. https://doi.org/10.20383/102.0440.

95. Szechtman H and Dvorkin-Gheva A. Project_09 Q27 StudyTitle Probing the environmental modulation of sensitization and compulsive checking: effect of having a different environment for the development and the expression of behavior under QNP. Federated Research Data Repository. 2021. https://doi.org/10.20383/102.0437.

96. Szechtman H, Dvorkin-Gheva A and Tucci MC. Project_10 Q31 StudyTitle Probing for comorbidity: evidence for other psychiatric disorders in compulsive checking. Federated Research Data Repository. 2021. https://doi.org/10.20383/102.0433.

97. Szechtman H and Dvorkin-Gheva A. Project_11 Q48 StudyTitle Probing for biological markers of the pathogenesis of compulsive checking: mRNA expression and changes in gut microbiota. Federated Research Data Repository. 2021. https://doi.org/10.20383/102.0417.

98. Szechtman H, Dvorkin-Gheva A and Tucci MC. Project_12 Q40 StudyTitle Resynthesis of compulsive checking: NAc lesion plus 8OHDPAT (0.0625 mg/kg) on injections 1-4 and mCPP (0.625 mg/kg) on injections 5-10. Federated Research Data Repository. 2021. https://doi.org/10.20383/102.0425.

99. Szechtman H, Dvorkin-Gheva A and Tucci MC. Project_12 Q43 StudyTitle Resynthesis of compulsive checking: NAc lesion plus 8OHDPAT (0.0625 mg/kg) on injections 1-4. Federated Research Data Repository. 2021. https://doi.org/10.20383/102.0422.

100. Szechtman H, Dvorkin-Gheva A and Tucci MC. Project_12 Q41 StudyTitle Resynthesis of compulsive checking: NAc lesion plus mCPP (0.625 mg/kg) on injections 1-6 and 8OHDPAT (0.0625 mg/kg) on injections 7-10. Federated Research Data Repository. 2021. https://doi.org/10.20383/102.0424.

101. Szechtman H, Dvorkin-Gheva A and Tucci MC. Project_12 Q42 StudyTitle Resynthesis of compulsive checking: NAc lesion plus mCPP (0.625 mg/kg) on injections 1-4. Federated Research Data Repository. 2021. https://doi.org/10.20383/102.0423.

102. Szechtman H, Dvorkin-Gheva A and Ray LB. A Virtual Library for Behavioral Performance in Standard Conditions – Experiment 01 Q22 Effect of an NMDA lesion of the infralimbic ctx on expression of compulsive checking. GigaScience Database. 2022.

103. Szechtman H, Dvorkin-Gheva A and Bisnaire LD. A Virtual Library for Behavioral Performance in Standard Conditions – Experiment 02 Q25 Effect of an NMDA lesion of the BLA on the development of sensitization and compulsive checking. GigaScience Database. 2022.

104. Szechtman H, Dvorkin-Gheva A and Bisnaire LD. A Virtual Library for Behavioral Performance in Standard Conditions – Experiment 03 Q25 Effect of an NMDA lesion of

the BLA on the expression of sensitization and compulsive checking. GigaScience Database. 2022.

105. Szechtman H, Dvorkin-Gheva A and McMurran TJA. A Virtual Library for Behavioral Performance in Standard Conditions – Experiment 04 Q28 Effect of an NMDA lesion of the OFC on the development of sensitization and compulsive checking. GigaScience Database. 2022.

106. Szechtman H, Dvorkin-Gheva A and McMurran TJA. A Virtual Library for Behavioral Performance in Standard Conditions – Experiment 05 Q28 Effect of an NMDA lesion of the OFC on the expression of sensitization and compulsive checking. GigaScience Database. 2022.

107. Szechtman H, Dvorkin-Gheva A, Silva CA and Ballester Gonzalez J. A Virtual Library for Behavioral Performance in Standard Conditions – Experiment 06 Q26 Effect of an NMDA lesion of the NAc on the development of sensitization and compulsive checking (batch 1). GigaScience Database. 2022.

108. Szechtman H, Dvorkin-Gheva A, Silva CA and Ballester Gonzalez J. A Virtual Library for Behavioral Performance in Standard Conditions – Experiment 07 Q26 Effect of an NMDA lesion of the NAc on the development of sensitization and compulsive checking (batch 2). GigaScience Database. 2022.

109. Szechtman H, Dvorkin-Gheva A, Silva CA and Ballester Gonzalez J. A Virtual Library for Behavioral Performance in Standard Conditions – Experiment 08 Q26 Effect of an NMDA lesion of the NAc on the expression of sensitization and compulsive checking. GigaScience Database. 2022.

110. Szechtman H, Dvorkin-Gheva A and Perreault ML. A Virtual Library for Behavioral Performance in Standard Conditions – Experiment 09 Q23 Effect of co-treatment with kappa agonist U69593 and QNP on the development of sensitization and compulsive checking. GigaScience Database. 2022.

111. Szechtman H and Dvorkin-Gheva A. A Virtual Library for Behavioral Performance in Standard Conditions – Experiment 10 Q29 Effect of CP809101 (5HT2C agonist) on the expression of sensitization and compulsive checking in an open field bordered by a low ledge. GigaScience Database. 2022.

112. Szechtman H and Dvorkin-Gheva A. A Virtual Library for Behavioral Performance in Standard Conditions – Experiment 11 Q33 Effect of substitution of QNP with 8OHDPAT on the expression of sensitization and compulsive checking. GigaScience Database. 2022.

113. Szechtman H, Dvorkin-Gheva A and Alkhatib AH. A Virtual Library for Behavioral Performance in Standard Conditions – Experiment 12 Q34 Comparison of the QNP (0.2 mg/kg) and the 8OHDPAT (1 mg/kg) effects on the development of sensitization and compulsive checking. GigaScience Database. 2022.

114. Szechtman H, Dvorkin-Gheva A and Alkhatib AH. A Virtual Library for Behavioral Performance in Standard Conditions – Experiment 13 Q34 Effect of QNP+8OHDPAT

co-treatment on the expression of sensitization and compulsive checking. GigaScience Database. 2022.

115. Szechtman H and Dvorkin-Gheva A. A Virtual Library for Behavioral Performance in Standard Conditions – Experiment 14 Q45 Effect of a low dose of 8OHDPAT (0.0625) co-injected with a threshold dose of QNP (0.0625 mg/kg) on the development of sensitization and compulsive checking. GigaScience Database. 2022.

116. Szechtman H and Dvorkin-Gheva A. A Virtual Library for Behavioral Performance in Standard Conditions – Experiment 15 Q46 Effect of 8OHDPAT dose (0 0.03125 0.0625 0.125 mg/kg) co-injected with a low dose of QNP (0.125 mg/kg) on the development of sensitization and compulsive checking (batch 1). GigaScience Database. 2022.

117. Szechtman H and Dvorkin-Gheva A. A Virtual Library for Behavioral Performance in Standard Conditions – Experiment 16 Q47 Effect of 8OHDPAT dose (0 0.03125 0.0625 0.125 mg/kg) co-injected with a low dose of QNP (0.125 mg/kg) on the development of sensitization and compulsive checking (batch 2). GigaScience Database. 2022.

118. Szechtman H and Dvorkin-Gheva A. A Virtual Library for Behavioral Performance in Standard Conditions – Experiment 17 Q30 Effect of QNP dose (0 0.125 0.25 and 0.5 mg/kg) on the development of sensitization and compulsive checking (batch 1). GigaScience Database. 2022.

119. Szechtman H and Dvorkin-Gheva A. A Virtual Library for Behavioral Performance in Standard Conditions – Experiment 18 Q30 Effect of QNP dose (0 0.0312 0.0625 0.125 0.25 and 0.5 mg/kg) on the development of sensitization and compulsive checking (batch 2). GigaScience Database. 2022.

120. Szechtman H, Dvorkin-Gheva A and Alkhatib AH. A Virtual Library for Behavioral Performance in Standard Conditions – Experiment 19 Q35 Effect of 8OHDPAT dose (0 0.03 0.125 0.25 and 1 mg/kg) on the development of sensitization and compulsive checking. GigaScience Database. 2022.

121. Szechtman H, Dvorkin-Gheva A and Tucci MC. A Virtual Library for Behavioral Performance in Standard Conditions – Experiment 20 Q36 Effect of mCPP dose (0 0.3125 0.625 or 1.25 mg/kg) on the development of sensitization and compulsive checking. GigaScience Database. 2022.

122. Szechtman H, Dvorkin-Gheva A and Tucci MC. A Virtual Library for Behavioral Performance in Standard Conditions – Experiment 21 Q37 Effect of mCPP dose (0.625 and 1.25 mg/kg) co-injected with a fixed dose of QNP (0.125 mg/kg) on the development of sensitization and induction of compulsive checking. GigaScience Database. 2022.

123. Szechtman H, Dvorkin-Gheva A and Tucci MC. A Virtual Library for Behavioral Performance in Standard Conditions – Experiment 22 Q38 Effect of mCPP dose (0.625 and 1.25 mg/kg) co-injected with a fixed dose of QNP (0.25 mg/kg) on the development of sensitization and compulsive checking. GigaScience Database. 2022.

124. Szechtman H, Dvorkin-Gheva A and Tucci MC. A Virtual Library for Behavioral Performance in Standard Conditions – Experiment 23 Q39 Effect of mCPP dose (0.3125 0.625 or 1.25 mg/kg) on the expression of sensitization and compulsive checking induced by QNP (0.125 mg/kg). GigaScience Database. 2022.

125. Szechtman H, Dvorkin-Gheva A and Alkhatib AH. A Virtual Library for Behavioral Performance in Standard Conditions – Experiment 24 Q34 Effect of acute escitalopram on the acute response to 8OHDPAT. GigaScience Database. 2022.

126. Szechtman H, Dvorkin-Gheva A and Alkhatib AH. A Virtual Library for Behavioral Performance in Standard Conditions – Experiment 25 Q34 Effect of acute escitalopram on the expression of sensitization and compulsive checking. GigaScience Database. 2022.

127. Szechtman H and Dvorkin-Gheva A. A Virtual Library for Behavioral Performance in Standard Conditions – Experiment 26 Q33 Effect of acute escitalopram or imipramine on the expression of compulsive checking. GigaScience Database. 2022.

128. Szechtman H, Dvorkin-Gheva A and Alkhatib AH. A Virtual Library for Behavioral Performance in Standard Conditions – Experiment 27 Q35 Effect of acute escitalopram on the expression of sensitization and compulsive checking. GigaScience Database. 2022.

129. Szechtman H, Dvorkin-Gheva A and Tucci MC. A Virtual Library for Behavioral Performance in Standard Conditions – Experiment 28 Q44 Effect of ritanserin (5-HT2A/C receptors blocker) on the attenuation by mCPP of the expression of quinpirole-induced compulsive checking. GigaScience Database. 2022.

130. Szechtman H, Dvorkin-Gheva A, Culver KE and Waxman D. A Virtual Library for Behavioral Performance in Standard Conditions – Experiment 29 Q17 Differential effects of clorgyline (a MAO inhibitor) on sensitization to QNP in rats tested in small and large environments. GigaScience Database. 2022.

131. Szechtman H, Dvorkin-Gheva A, Culver KE and Waxman D. A Virtual Library for Behavioral Performance in Standard Conditions – Experiment 30 Q18 Effects of hypophysectomy on compulsive checking and cortical dendrites. GigaScience Database. 2022.

132. Szechtman H, Dvorkin-Gheva A and Perreault ML. A Virtual Library for Behavioral Performance in Standard Conditions – Experiment 31 Q21 Development and temporal organization of compulsive checking induced by repeated injections of QNP (0.5 mg/kg). GigaScience Database. 2022.

133. Szechtman H, Dvorkin-Gheva A and Perreault ML. A Virtual Library for Behavioral Performance in Standard Conditions – Experiment 32 Q21 Effect of separating pups from maternal nest for 15 or 180 min on postnatal days 2 to 15 on the development of compulsive checking induced by repeated injections of quinpirole in adult male rats. GigaScience Database. 2022.

134. Szechtman H, Dvorkin-Gheva A and Ray LB. A Virtual Library for Behavioral Performance in Standard Conditions – Experiment 33 Q22 Effect of testing under infra-

red illumination on the expression of sensitization and compulsive checking. GigaScience Database. 2022.

135. Szechtman H and Dvorkin-Gheva A. A Virtual Library for Behavioral Performance in Standard Conditions – Experiment 34 Q24 Effect of a circular arena on development of sensitization and compulsive checking induced by QNP (0.5 mg/kg). GigaScience Database. 2022.

136. Szechtman H and Dvorkin-Gheva A. A Virtual Library for Behavioral Performance in Standard Conditions – Experiment 35 Q27 Effect of sensitization in activity chambers or in the home cage on performance of compulsive checking in the open field. GigaScience Database. 2022.

137. Szechtman H and Dvorkin-Gheva A. A Virtual Library for Behavioral Performance in Standard Conditions – Experiment 36 Q29 Effect of a small ledge on open field on the development of sensitization and compulsive checking. GigaScience Database. 2022.

138. Szechtman H and Dvorkin-Gheva A. A Virtual Library for Behavioral Performance in Standard Conditions – Experiment 37 Q47 Effect of testing with the 4 objects removed from open field on the expression of sensitization and compulsive checking. GigaScience Database. 2022.

139. Szechtman H, Dvorkin-Gheva A and Tucci MC. A Virtual Library for Behavioral Performance in Standard Conditions – Experiment 38 Q31 Effect on performance in tests of depression and anxiety by rats expressing compulsive checking. GigaScience Database. 2022.

140. Szechtman H and Dvorkin-Gheva A. A Virtual Library for Behavioral Performance in Standard Conditions – Experiment 39 Q48 Changes in gut microbiota during development of compulsive checking and locomotor sensitization induced by chronic treatment with QNP (0.25 mg/kg). GigaScience Database. 2022.

141. Szechtman H, Dvorkin-Gheva A and Tucci MC. A Virtual Library for Behavioral Performance in Standard Conditions – Experiment 40 Q40 Effects of NAc lesion plus 8OHDPAT (.0625 mg/kg on injections 1-4) or mCPP (0.625 mg/kg on injections 5-10) in producing compulsive checking. GigaScience Database. 2022.

142. Szechtman H, Dvorkin-Gheva A and Tucci MC. A Virtual Library for Behavioral Performance in Standard Conditions – Experiment 41 Q43 Effect of NAc lesion plus 8OHDPAT (0.0625 mg/kg on injections 1-4) in producing compulsive checking. GigaScience Database. 2022.

143. Szechtman H, Dvorkin-Gheva A and Tucci MC. A Virtual Library for Behavioral Performance in Standard Conditions – Experiment 42 Q41 Effects of NAc lesion plus mCPP (0.625 mg/kg on injections 1-6) or 8OHDPAT (.0625 mg/kg on injections 7-10) in producing compulsive checking. GigaScience Database. 2022.

144. Szechtman H, Dvorkin-Gheva A and Tucci MC. A Virtual Library for Behavioral Performance in Standard Conditions – Experiment 43 Q42 Effect of NAc lesion plus

mCPP (0.625 mg/kg on injections 1-4) in producing compulsive checking. GigaScience Database. 2022.

TABLES

Table 1. Projects, experiments and studies in datasets deposited in FRDR and GigaDB repositories

Project #	Title of Project	Experiment #	Title of Experiment	# of Trials in Experiment	Study Q##	Study Experiment Code	Title of Q## Study	Title of Q## Dataset in FRDR Repository	DOI of Q## Dataset in FRDR Repository	Title of Experiment ## Dataset in GigaDB Repository	DOI of Experiment ## Dataset in GigaDB Repository
1	Probing the neural circuit mediating sensitization and compulsive checking	1	Effect of an NMDA lesion of the infralimbic ctx on expression of compulsive checking	1621	Q22	ILi2	Probing the neural circuit mediating sensitization and compulsive checking: role of the infralimbic (IL) cortex on expression	Project_01 Q22 StudyTitle Probing the neural circuit mediating sensitization and compulsive checking: role of the infralimbic (IL) cortex on expression	10.20383/102.0442	A Virtual Library for Behavioral Performance in Standard Conditions – Experiment 01 Q22 Effect of an NMDA lesion of the infralimbic ctx on expression of compulsive checking	insert here
1	Probing the neural circuit mediating sensitization and compulsive checking	2	Effect of an NMDA lesion of the BLA on the development of sensitization and compulsive checking	784	Q25	Bla1	Probing the neural circuit mediating sensitization and compulsive checking: role of the basal lateral amygdala (BLA) on development	Project_01 Q25 StudyTitle Probing the neural circuit mediating sensitization and compulsive checking: role of the basal lateral amygdala (BLA) on development, and, on expression	10.20383/102.0439	A Virtual Library for Behavioral Performance in Standard Conditions – Experiment 02 Q25 Effect of an NMDA lesion of the BLA on the development of sensitization and compulsive checking	insert here
1	Probing the neural circuit mediating sensitization and compulsive checking	3	Effect of an NMDA lesion of the BLA on the expression of sensitization and compulsive checking	778	Q25	Bla2	Probing the neural circuit mediating sensitization and compulsive checking: role of the basal lateral amygdala (BLA) on expression	Project_01 Q25 StudyTitle Probing the neural circuit mediating sensitization and compulsive checking: role of the basal lateral amygdala (BLA) on development, and, on expression	10.20383/102.0439	A Virtual Library for Behavioral Performance in Standard Conditions – Experiment 03 Q25 Effect of an NMDA lesion of the BLA on the expression of sensitization and compulsive checking	insert here
1	Probing the neural circuit mediating sensitization and compulsive checking	4	Effect of an NMDA lesion of the OFC on the development of sensitization and compulsive checking	783	Q28	OFC1	Probing the neural circuit mediating sensitization and compulsive checking: role of the lateral orbitofrontal cortex (OFC) on development	Project_01 Q28 StudyTitle Probing the neural circuit mediating sensitization and compulsive checking: role of the lateral orbitofrontal cortex (OFC) on development, and, on expression	10.20383/102.0436	A Virtual Library for Behavioral Performance in Standard Conditions – Experiment 04 Q28 Effect of an NMDA lesion of the OFC on the development of sensitization and	insert here

										compulsive checking	
1	Probing the neural circuit mediating sensitization and compulsive checking	5	Effect of an NMDA lesion of the OFC on the expression of sensitization and compulsive checking	768	Q28	OFC2	Probing the neural circuit mediating sensitization and compulsive checking: role of the lateral orbitofrontal cortex (OFC) on expression	Project_01 Q28 StudyTitle Probing the neural circuit mediating sensitization and compulsive checking: role of the lateral orbitofrontal cortex (OFC) on development, and, on expression	10.20383/102.0436	A Virtual Library for Behavioral Performance in Standard Conditions – Experiment 05 Q28 Effect of an NMDA lesion of the OFC on the expression of sensitization and compulsive checking	insert here
1	Probing the neural circuit mediating sensitization and compulsive checking	6	Effect of an NMDA lesion of the NAc on the development of sensitization and compulsive checking (batch 1)	768	Q26	NAc1	Probing the neural circuit mediating sensitization and compulsive checking: role of the nucleus accumbens core (NAc) on development	Project_01 Q26 StudyTitle Probing the neural circuit mediating sensitization and compulsive checking: role of the nucleus accumbens core (NAc) on development, and, on expression	10.20383/102.0438	A Virtual Library for Behavioral Performance in Standard Conditions – Experiment 06 Q26 Effect of an NMDA lesion of the NAc on the development of sensitization and compulsive checking (batch 1)	insert here
1	Probing the neural circuit mediating sensitization and compulsive checking	7	Effect of an NMDA lesion of the NAc on the development of sensitization and compulsive checking (batch 2)	768	Q26	NAc1	Probing the neural circuit mediating sensitization and compulsive checking: role of the nucleus accumbens core (NAc) on development	Project_01 Q26 StudyTitle Probing the neural circuit mediating sensitization and compulsive checking: role of the nucleus accumbens core (NAc) on development, and, on expression	10.20383/102.0438	A Virtual Library for Behavioral Performance in Standard Conditions – Experiment 07 Q26 Effect of an NMDA lesion of the NAc on the development of sensitization and compulsive checking (batch 2)	insert here
1	Probing the neural circuit mediating sensitization and compulsive checking	8	Effect of an NMDA lesion of the NAc on the expression of sensitization and compulsive checking	767	Q26	NAc2	Probing the neural circuit mediating sensitization and compulsive checking: role of the nucleus accumbens core (NAc) on expression	Project_01 Q26 StudyTitle Probing the neural circuit mediating sensitization and compulsive checking: role of the nucleus accumbens core (NAc) on development, and, on expression	10.20383/102.0438	A Virtual Library for Behavioral Performance in Standard Conditions – Experiment 08 Q26 Effect of an NMDA lesion of the NAc on the expression of sensitization and compulsive checking	insert here
2	Probing the neurochemistry of sensitization and compulsive checking	9	Effect of co-treatment with kappa agonist U69593 and QNP on the development of sensitization and compulsive checking	599	Q23	U693	Probing the neurochemistry of sensitization and compulsive checking: dopamine and kappa opioid systems	Project_02 Q23 StudyTitle Probing the neurochemistry of sensitization and compulsive checking: dopamine and kappa opioid systems	10.20383/102.0441	A Virtual Library for Behavioral Performance in Standard Conditions – Experiment 09 Q23 Effect of co-treatment with kappa agonist U69593	insert here

									and QNP on the development of sensitization and compulsive checking	
2	Probing the neurochemistry of sensitization and compulsive checking	10	Effect of CP809101 (5HT2C agonist) on the expression of sensitization and compulsive checking in an open field bordered by a low ledge	32	Q29	WALL	Probing the neurochemistry of sensitization and compulsive checking: dopamine and serotonin systems	Project_09 Q29 StudyTitle Probing the environmental modulation of sensitization and compulsive checking: influence of a small ledge around OF	10.20383/102.0435	A Virtual Library for Behavioral Performance in Standard Conditions – Experiment 10 Q29 Effect of CP809101 (5HT2C agonist) on the expression of sensitization and compulsive checking in an open field bordered by a low ledge	insert here
2	Probing the neurochemistry of sensitization and compulsive checking	11	Effect of substitution of QNP with 8OHDPAT on the expression of sensitization and compulsive checking	48	Q33	AD8O	Probing the neurochemistry of sensitization and compulsive checking: dopamine and serotonin systems	Project_04 Q33 StudyTitle Probing for drugs that mitigate sensitization and compulsive checking: acute treatment with escitalopram or imipramine	10.20383/102.0432	A Virtual Library for Behavioral Performance in Standard Conditions – Experiment 11 Q33 Effect of substitution of QNP with 8OHDPAT on the expression of sensitization and compulsive checking	insert here
2	Probing the neurochemistry of sensitization and compulsive checking	12	Comparison of the QNP (0.2 mg/kg) and the 8OHDPAT (1 mg/kg) effects on the development of sensitization and compulsive checking	462	Q34	QN8O	Probing the neurochemistry of sensitization and compulsive checking: dopamine and serotonin systems	Project_02 Q34 StudyTitle Probing the neurochemistry of sensitization and compulsive checking: dopamine and serotonin systems	10.20383/102.0431	A Virtual Library for Behavioral Performance in Standard Conditions – Experiment 12 Q34 Comparison of the QNP (0.2 mg/kg) and the 8OHDPAT (1 mg/kg) effects on the development of sensitization and compulsive checking	insert here
2	Probing the neurochemistry of sensitization and compulsive checking	13	Effect of QNP+8OHDPAT co-treatment on the expression of sensitization and compulsive checking	36	Q34	QN8O	Probing the neurochemistry of sensitization and compulsive checking: dopamine and serotonin systems	Project_02 Q34 StudyTitle Probing the neurochemistry of sensitization and compulsive checking: dopamine and serotonin systems	10.20383/102.0431	A Virtual Library for Behavioral Performance in Standard Conditions – Experiment 13 Q34 Effect of QNP+8OHDPAT co-treatment on the	insert here

										expression of sensitization and compulsive checking	
2	Probing the neurochemistry of sensitization and compulsive checking	14	Effect of a low dose of 8OHDPAT (0.0625) co-injected with a threshold dose of QNP (0.0625 mg/kg) on the development of sensitization and compulsive checking	357	Q45	QIDI	Probing the neurochemistry of sensitization and compulsive checking: dopamine and serotonin systems	Project_02 Q45 StudyTitle Probing the neurochemistry of sensitization and compulsive checking: dopamine and serotonin systems	10.20383/102.0420	A Virtual Library for Behavioral Performance in Standard Conditions – Experiment 14 Q45 Effect of a low dose of 8OHDPAT (0.0625) co-injected with a threshold dose of QNP (0.0625 mg/kg) on the development of sensitization and compulsive checking	insert here
2	Probing the neurochemistry of sensitization and compulsive checking	15	Effect of 8OHDPAT dose (0 0.03125 0.0625 0.125 mg/kg) co-injected with a low dose of QNP (0.125 mg/kg) on the development of sensitization and compulsive checking (batch 1)	728	Q46	QMD3	Probing the neurochemistry of sensitization and compulsive checking: dopamine and serotonin systems	Project_02 Q46 StudyTitle Probing the neurochemistry of sensitization and compulsive checking: dopamine and serotonin systems	10.20383/102.0419	A Virtual Library for Behavioral Performance in Standard Conditions – Experiment 15 Q46 Effect of 8OHDPAT dose (0 0.03125 0.0625 0.125 mg/kg) co-injected with a low dose of QNP (0.125 mg/kg) on the development of sensitization and compulsive checking (batch 1)	insert here
2	Probing the neurochemistry of sensitization and compulsive checking	16	Effect of 8OHDPAT dose (0 0.03125 0.0625 0.125 mg/kg) co-injected with a low dose of QNP (0.125 mg/kg) on the development of sensitization and compulsive checking (batch 2)	718	Q47	QMD3	Probing the neurochemistry of sensitization and compulsive checking: dopamine and serotonin systems	Project_02 Q47 StudyTitle Probing the neurochemistry of sensitization and compulsive checking: dopamine and serotonin systems	10.20383/102.0418	A Virtual Library for Behavioral Performance in Standard Conditions – Experiment 16 Q47 Effect of 8OHDPAT dose (0 0.03125 0.0625 0.125 mg/kg) co-injected with a low dose of QNP (0.125 mg/kg) on the development of sensitization and compulsive checking (batch 2)	insert here

3	Pharmacology of sensitization and compulsive checking	17	Effect of QNP dose (0 0.125 0.25 and 0.5 mg/kg) on the development of sensitization and compulsive checking (batch 1)	783	Q30	DRCq	Pharmacology of sensitization and compulsive checking: dose-response profile of quinpirole (0 0.0312 0.0625 0.125 0.25 and 0.5 mg/kg)	Project_03 Q30 StudyTitle Pharmacology of sensitization and compulsive checking: dose-response profile of quinpirole (0 0.0312 0.0625 0.125 0.25 and 0.5 mg/kg)	10.20383/102.0434	A Virtual Library for Behavioral Performance in Standard Conditions – Experiment 17 Q30 Effect of QNP dose (0 0.125 0.25 and 0.5 mg/kg) on the development of sensitization and compulsive checking (batch 1)	insert here
3	Pharmacology of sensitization and compulsive checking	18	Effect of QNP dose (0 0.0312 0.0625 0.125 0.25 and 0.5 mg/kg) on the development of sensitization and compulsive checking (batch 2)	787	Q30	DRCq	Pharmacology of sensitization and compulsive checking: dose-response profile of quinpirole (0 0.0312 0.0625 0.125 0.25 and 0.5 mg/kg)	Project_03 Q30 StudyTitle Pharmacology of sensitization and compulsive checking: dose-response profile of quinpirole (0 0.0312 0.0625 0.125 0.25 and 0.5 mg/kg)	10.20383/102.0434	A Virtual Library for Behavioral Performance in Standard Conditions – Experiment 18 Q30 Effect of QNP dose (0 0.0312 0.0625 0.125 0.25 and 0.5 mg/kg) on the development of sensitization and compulsive checking (batch 2)	insert here
3	Pharmacology of sensitization and compulsive checking	19	Effect of 8OHDPAT dose (0 0.03 0.125 0.25 and 1 mg/kg) on the development of sensitization and compulsive checking	728	Q35	8Odr	Pharmacology of sensitization and compulsive checking: dose-response profile of 8OHDPAT (0 0.03 0.125 0.25 and 1 mg/kg)	Project_03 Q35 StudyTitle Pharmacology of sensitization and compulsive checking: dose-response profile of 8OHDPAT (0 0.03 0.125 0.25 and 1 mg/kg)	10.20383/102.0430	A Virtual Library for Behavioral Performance in Standard Conditions – Experiment 19 Q35 Effect of 8OHDPAT dose (0 0.03 0.125 0.25 and 1 mg/kg) on the development of sensitization and compulsive checking	insert here
3	Pharmacology of sensitization and compulsive checking	20	Effect of mCPP dose (0 0.3125 0.625 or 1.25 mg/kg) on the development of sensitization and compulsive checking	554	Q36	mCPP	Pharmacology of sensitization and compulsive checking: dose-response profile of mCPP (0 0.3125 0.625 or 1.25 mg/kg)	Project_03 Q36 StudyTitle Pharmacology of sensitization and compulsive checking: dose-response profile of mCPP (0 0.3125 0.625 or 1.25 mg/kg)	10.20383/102.0429	A Virtual Library for Behavioral Performance in Standard Conditions – Experiment 20 Q36 Effect of mCPP dose (0 0.3125 0.625 or 1.25 mg/kg) on the development of sensitization and compulsive checking	insert here
4	Probing for drugs that mitigate	21	Effect of mCPP dose (0.625 and 1.25 mg/kg)	546	Q37	mCPq	Probing for drugs that mitigate sensitization and	Project_04 Q37 StudyTitle Probing for drugs that mitigate	10.20383/102.0428	A Virtual Library for Behavioral Performance	insert here

	sensitization and compulsive checking		co-injected with a fixed dose of QNP (0.125 mg/kg) on the development of sensitization and induction of compulsive checking				compulsive checking: effect of mCPP dose (0 0.625 and 1.25 mg/kg) on development produced by a low dose of QNP (0.125 mg/kg)	sensitization and compulsive checking: effect of mCPP dose (0 0.625 and 1.25 mg/kg) on development produced by a low dose of QNP (0.125 mg/kg)		in Standard Conditions – Experiment 21 Q37 Effect of mCPP dose (0.625 and 1.25 mg/kg) co-injected with a fixed dose of QNP (0.125 mg/kg) on the development of sensitization and induction of compulsive checking	
4	Probing for drugs that mitigate sensitization and compulsive checking	22	Effect of mCPP dose (0.625 and 1.25 mg/kg) co-injected with a fixed dose of QNP (0.25 mg/kg) on the development of sensitization and compulsive checking	635	Q38	mCPh	Probing for drugs that mitigate sensitization and compulsive checking: effect of mCPP dose (0 0.625 and 1.25 mg/kg) on development produced by a medium dose of QNP (0.25 mg/kg)	Project_04 Q38 StudyTitle Probing for drugs that mitigate sensitization and compulsive checking: effect of mCPP dose (0 0.625 and 1.25 mg/kg) on development produced by a medium dose of QNP (0.25 mg/kg)	10.20383/102.0427	A Virtual Library for Behavioral Performance in Standard Conditions – Experiment 22 Q38 Effect of mCPP dose (0.625 and 1.25 mg/kg) co-injected with a fixed dose of QNP (0.25 mg/kg) on the development of sensitization and compulsive checking	insert here
4	Probing for drugs that mitigate sensitization and compulsive checking	23	Effect of mCPP dose (0.3125 0.625 or 1.25 mg/kg) on the expression of sensitization and compulsive checking induced by QNP (0.125 mg/kg)	767	Q39	mCPr	Probing for drugs that mitigate sensitization and compulsive checking: effect of mCPP dose (0 0.3125 0.625 and 1.25 mg/kg) on expression induced by a low dose of QNP (0.125 mg/kg)	Project_04 Q39 StudyTitle Probing for drugs that mitigate sensitization and compulsive checking: effect of mCPP dose (0 0.3125 0.625 and 1.25 mg/kg) on expression induced by a low dose of QNP (0.125 mg/kg)	10.20383/102.0426	A Virtual Library for Behavioral Performance in Standard Conditions – Experiment 23 Q39 Effect of mCPP dose (0.3125 0.625 or 1.25 mg/kg) on the expression of sensitization and compulsive checking induced by QNP (0.125 mg/kg)	insert here
4	Probing for drugs that mitigate sensitization and compulsive checking	24	Effect of acute escitalopram on the acute response to 8OHDPAT	12	Q34	QN8O	Probing for drugs that mitigate sensitization and compulsive checking: acute treatment with escitalopram on acute 8OHDPAT	Project_02 Q34 StudyTitle Probing the neurochemistry of sensitization and compulsive checking: dopamine and serotonin systems	10.20383/102.0431	A Virtual Library for Behavioral Performance in Standard Conditions – Experiment 24 Q34 Effect of acute escitalopram on the acute response to 8OHDPAT	insert here
4	Probing for drugs that mitigate	25	Effect of acute escitalopram on the	72	Q34	QN8O	Probing for drugs that mitigate sensitization and	Project_02 Q34 StudyTitle Probing the neurochemistry of	10.20383/102.0431	A Virtual Library for Behavioral Performance	insert here

	sensitization and compulsive checking		expression of sensitization and compulsive checking				compulsive checking: acute treatment with escitalopram	sensitization and compulsive checking: dopamine and serotonin systems		in Standard Conditions – Experiment 25 Q34 Effect of acute escitalopram on the expression of sensitization and compulsive checking	
4	Probing for drugs that mitigate sensitization and compulsive checking	26	Effect of acute escitalopram or imipramine on the expression of compulsive checking	526	Q33	AD8O	Probing for drugs that mitigate sensitization and compulsive checking: acute treatment with escitalopram or imipramine	Project_04 Q33 StudyTitle Probing for drugs that mitigate sensitization and compulsive checking: acute treatment with escitalopram or imipramine	10.20383/102.0432	A Virtual Library for Behavioral Performance in Standard Conditions – Experiment 26 Q33 Effect of acute escitalopram or imipramine on the expression of compulsive checking	insert here
4	Probing for drugs that mitigate sensitization and compulsive checking	27	Effect of acute escitalopram on the expression of sensitization and compulsive checking	112	Q35	8Odr	Probing for drugs that mitigate sensitization and compulsive checking: acute treatment with escitalopram	Project_03 Q35 StudyTitle Pharmacology of sensitization and compulsive checking: dose-response profile of 8OHDPAT (0 0.03 0.125 0.25 and 1 mg/kg)	10.20383/102.0430	A Virtual Library for Behavioral Performance in Standard Conditions – Experiment 27 Q35 Effect of acute escitalopram on the expression of sensitization and compulsive checking	insert here
5	Probing the mode of action of mCPP on compulsive checking	28	Effect of ritanserin (5-HT2A/C receptors blocker) on the attenuation by mCPP of the expression of quinpirole-induced compulsive checking	638	Q44	5HT5	Probing the mode of action of mCPP on compulsive checking: 5-HT2A/C receptors	Project_05 Q44 StudyTitle Probing the mode of action of mCPP on compulsive checking: 5-HT2A/C receptors	10.20383/102.0421	A Virtual Library for Behavioral Performance in Standard Conditions – Experiment 28 Q44 Effect of ritanserin (5-HT2A/C receptors blocker) on the attenuation by mCPP of the expression of quinpirole-induced compulsive checking	insert here
6	Probing the mode of action of quinpirole on behavior	29	Differential effects of clorgyline (a MAO inhibitor) on sensitization to QNP in rats tested in small and large environments	137	Q17	Clg3	Probing the mode of action of quinpirole on behavior: role of the monoamine oxidase inhibitor (MAOI) sensitive quinpirole binding site (MQB)	Project_06 Q17 StudyTitle Probing the mode of action of quinpirole on behavior: role of the monoamine oxidase inhibitor (MAOI) sensitive quinpirole binding site (MQB)	10.20383/102.0445	A Virtual Library for Behavioral Performance in Standard Conditions – Experiment 29 Q17 Differential effects of clorgyline (a MAO inhibitor) on sensitization to QNP in rats tested in small and large	insert here

										environments	
7	Probing the role of hormones in compulsive checking	30	Effects of hypophysectomy on compulsive checking and cortical dendrites	144	Q18	Hypx	Probing the role of hormones in compulsive checking: Is the endocrine system necessary for compulsive behavior?	Project_07 Q18 StudyTitle Probing the role of hormones in compulsive checking: Is the endocrine system necessary for compulsive behavior?	10.20383/102.0444	A Virtual Library for Behavioral Performance in Standard Conditions – Experiment 30 Q18 Effects of hypophysectomy on compulsive checking and cortical dendrites	insert here
8	Probing the parameters of sensitization and compulsive checking	31	Development and temporal organization of compulsive checking induced by repeated injections of QNP (0.5 mg/kg)	144	Q21	MSe3	Probing the parameters of sensitization and compulsive checking: characteristics of behavioral measures during pathogenesis of compulsive behavior	Project_08 Q21 StudyTitle Probing the parameters of sensitization and compulsive checking: characteristics of behavioral measures during pathogenesis of compulsive behavior	10.20383/102.0443	A Virtual Library for Behavioral Performance in Standard Conditions – Experiment 31 Q21 Development and temporal organization of compulsive checking induced by repeated injections of QNP (0.5 mg/kg)	insert here
9	Probing the environmental modulation of sensitization and compulsive checking	32	Effect of separating pups from maternal nest for 15 or 180 min on postnatal days 2 to 15 on the development of compulsive checking induced by repeated injections of quinpirole in adult male rats	287	Q21	MSe3	Probing the environmental modulation of sensitization and compulsive checking: influence of post-natal environment	Project_08 Q21 StudyTitle Probing the parameters of sensitization and compulsive checking: characteristics of behavioral measures during pathogenesis of compulsive behavior	10.20383/102.0443	A Virtual Library for Behavioral Performance in Standard Conditions – Experiment 32 Q21 Effect of separating pups from maternal nest for 15 or 180 min on postnatal days 2 to 15 on the development of compulsive checking induced by repeated injections of quinpirole in adult male rats	insert here
9	Probing the environmental modulation of sensitization and compulsive checking	33	Effect of testing under infra-red illumination on the expression of sensitization and compulsive checking	96	Q22	ILi2	Probing the environmental modulation of sensitization and compulsive checking: influence of darkness on expression	Project_01 Q22 StudyTitle Probing the neural circuit mediating sensitization and compulsive checking: role of the infralimbic (IL) cortex on expression	10.20383/102.0442	A Virtual Library for Behavioral Performance in Standard Conditions – Experiment 33 Q22 Effect of testing under infra-red illumination on the expression of sensitization and compulsive checking	insert here

9	Probing the environmental modulation of sensitization and compulsive checking	34	Effect of a circular arena on development of sensitization and compulsive checking induced by QNP (0.5 mg/kg)	140	Q24	rats	Probing the environmental modulation of sensitization and compulsive checking: effects of a walled circular arena	Project_09 Q24 StudyTitle Probing the environmental modulation of sensitization and compulsive checking: effects of a walled circular arena	10.20383/102.0440	A Virtual Library for Behavioral Performance in Standard Conditions – Experiment 34 Q24 Effect of a circular arena on development of sensitization and compulsive checking induced by QNP (0.5 mg/kg)	insert here
9	Probing the environmental modulation of sensitization and compulsive checking	35	Effect of sensitization in activity chambers or in the home cage on performance of compulsive checking in the open field	320	Q27	OAH3	Probing the environmental modulation of sensitization and compulsive checking: effect of having a different environment for the development and the expression of behavior under QNP	Project_09 Q27 StudyTitle Probing the environmental modulation of sensitization and compulsive checking: effect of having a different environment for the development and the expression of behavior under QNP	10.20383/102.0437	A Virtual Library for Behavioral Performance in Standard Conditions – Experiment 35 Q27 Effect of sensitization in activity chambers or in the home cage on performance of compulsive checking in the open field	insert here
9	Probing the environmental modulation of sensitization and compulsive checking	36	Effect of a small ledge on open field on the development of sensitization and compulsive checking	176	Q29	WALL	Probing the environmental modulation of sensitization and compulsive checking: influence of a small ledge around OF	Project_09 Q29 StudyTitle Probing the environmental modulation of sensitization and compulsive checking: influence of a small ledge around OF	10.20383/102.0435	A Virtual Library for Behavioral Performance in Standard Conditions – Experiment 36 Q29 Effect of a small ledge on open field on the development of sensitization and compulsive checking	insert here
9	Probing the environmental modulation of sensitization and compulsive checking	37	Effect of testing with the 4 objects removed from open field on the expression of sensitization and compulsive checking	55	Q47	QMD3	Probing the environmental modulation of sensitization and compulsive checking: influence of no objects in the open field on expression	Project_02 Q47 StudyTitle Probing the neurochemistry of sensitization and compulsive checking: dopamine and serotonin systems	10.20383/102.0418	A Virtual Library for Behavioral Performance in Standard Conditions – Experiment 37 Q47 Effect of testing with the 4 objects removed from open field on the expression of sensitization and compulsive checking	insert here
10	Probing for comorbidity	38	Effect on performance in tests of depression and anxiety by rats expressing compulsive	557	Q31	Como	Probing for comorbidity: evidence for other psychiatric disorders in compulsive checking	Project_10 Q31 StudyTitle Probing for comorbidity: evidence for other psychiatric disorders in compulsive checking	10.20383/102.0433	A Virtual Library for Behavioral Performance in Standard Conditions – Experiment 38 Q31 Effect	insert here

			checking							on performance in tests of depression and anxiety by rats expressing compulsive checking	
11	Probing for biological markers of the pathogenesis of compulsive checking	39	Changes in gut microbiota during development of compulsive checking and locomotor sensitization induced by chronic treatment with QNP (0.25 mg/kg)	281	Q48	QRNA	Probing for biological markers of the pathogenesis of compulsive checking: mRNA expression and changes in gut microbiota	Project_11 Q48 StudyTitle Probing for biological markers of the pathogenesis of compulsive checking: mRNA expression and changes in gut microbiota	10.20383/102.0417	A Virtual Library for Behavioral Performance in Standard Conditions – Experiment 39 Q48 Changes in gut microbiota during development of compulsive checking and locomotor sensitization induced by chronic treatment with QNP (0.25 mg/kg)	insert here
12	Resynthesis of compulsive checking	40	Effects of NAc lesion plus 8OHDPAT (.0625 mg/kg on injections 1-4) or mCPP (0.625 mg/kg on injections 5-10) in producing compulsive checking	557	Q40	5HT1	Resynthesis of compulsive checking: NAc lesion plus 8OHDPAT (0.0625 mg/kg) on injections 1-4 and mCPP (0.625 mg/kg) on injections 5-10	Project_12 Q40 StudyTitle Resynthesis of compulsive checking: NAc lesion plus 8OHDPAT (0.0625 mg/kg) on injections 1-4 and mCPP (0.625 mg/kg) on injections 5-10	10.20383/102.0425	A Virtual Library for Behavioral Performance in Standard Conditions – Experiment 40 Q40 Effects of NAc lesion plus 8OHDPAT (.0625 mg/kg on injections 1-4) or mCPP (0.625 mg/kg on injections 5-10) in producing compulsive checking	insert here
12	Resynthesis of compulsive checking	41	Effect of NAc lesion plus 8OHDPAT (0.0625 mg/kg on injections 1-4) in producing compulsive checking	190	Q43	5HT4	Resynthesis of compulsive checking: NAc lesion plus 8OHDPAT (0.0625 mg/kg) on injections 1-4	Project_12 Q43 StudyTitle Resynthesis of compulsive checking: NAc lesion plus 8OHDPAT (0.0625 mg/kg) on injections 1-4	10.20383/102.0422	A Virtual Library for Behavioral Performance in Standard Conditions – Experiment 41 Q43 Effect of NAc lesion plus 8OHDPAT (0.0625 mg/kg on injections 1-4) in producing compulsive checking	insert here
12	Resynthesis of compulsive checking	42	Effects of NAc lesion plus mCPP (0.625 mg/kg on injections 1-6) or 8OHDPAT (.0625 mg/kg on injections 7-10) in	505	Q41	5HT2	Resynthesis of compulsive checking: NAc lesion plus mCPP (0.625 mg/kg) on injections 1-6 and 8OHDPAT (0.0625 mg/kg) on injections	Project_12 Q41 StudyTitle Resynthesis of compulsive checking: NAc lesion plus mCPP (0.625 mg/kg) on injections 1-6 and 8OHDPAT (0.0625 mg/kg) on	10.20383/102.0424	A Virtual Library for Behavioral Performance in Standard Conditions – Experiment 42 Q41 Effects of NAc lesion plus	insert here

			producing compulsive checking				7-10	injections 7-10		mCPP (0.625 mg/kg on injections 1-6) or 8OHDPAT (.0625 mg/kg on injections 7-10) in producing compulsive checking	
12	Resynthesis of compulsive checking	43	Effect of NAc lesion plus mCPP (0.625 mg/kg on injections 1-4) in producing compulsive checking	210	Q42	5HT3	Resynthesis of compulsive checking: NAc lesion plus mCPP (0.625 mg/kg) on injections 1-4	Project_12 Q42 StudyTitle Resynthesis of compulsive checking: NAc lesion plus mCPP (0.625 mg/kg) on injections 1-4	10.20383/102.0423	A Virtual Library for Behavioral Performance in Standard Conditions – Experiment 43 Q42 Effect of NAc lesion plus mCPP (0.625 mg/kg on injections 1-4) in producing compulsive checking	insert here

Table 2. Arenas in which rats were tested, illumination in the testing room, and number of tests recorded in each arena and illumination. Column labels with prefix APPARATUS_ are names of annotation metadata variables. For locale numeral names, see Figure 3 .

Arena type in which rat is tested			Items in testing arena			Open Field (OF) # and Room Address			Illumination in testing room with APPARATUS		
APPARATUS_ArenaType_value	APPARATUS_ArenaType_valueLabel	# of Trials	APPARATUS_ArenaObjects_value	APPARATUS_ArenaObjects_valueLabel	# of Trials	APPARATUS_ArenaID_value	APPARATUS_ArenaID_valueLabel	# of Trials	APPARATUS_ArenaTestingRoomLightCondition_value	APPARATUS_ArenaTestingRoomLightCondition_valueLabel	# of Trials
1	large Open Field, 160x160 cm table surface on 60 cm high legs	19623	1	objects in arena locales 10 14 5 and 8	18689	1	OF #1 in room U59_south	5099	0	testing room ILLUMINATED (fluorescent lights ON)	5087
									1	testing room DARK (infrared lights ON)	12
						2	OF #2 in room U59_north	4785	0	testing room ILLUMINATED (fluorescent lights ON)	4773
									1	testing room DARK (infrared lights ON)	12
						3	OF #3 in room U60_south	4437	0	testing room ILLUMINATED (fluorescent lights ON)	4425
									1	testing room DARK (infrared lights ON)	12
						4	OF #4 in room U60_north	4368	0	testing room ILLUMINATED (fluorescent lights ON)	4356
									1	testing room DARK (infrared lights ON)	12
			2	objects in arena locales 18 22 9 and 4 (objects ROTATION 180 deg)	879	1	OF #1 in room U59_south		0	testing room ILLUMINATED (fluorescent lights ON)	226
						2	OF #2 in room U59_north		0	testing room ILLUMINATED (fluorescent lights ON)	225
						3	OF #3 in room U60_south		0	testing room ILLUMINATED (fluorescent lights ON)	215
						4	OF #4 in room U60_north		0	testing room ILLUMINATED (fluorescent lights ON)	213
			0	no objects in arena	55	1	OF #1 in room U59_south		0	testing room ILLUMINATED (fluorescent lights ON)	14
						2	OF #2 in room U59_north		0	testing room ILLUMINATED (fluorescent lights ON)	14
						3	OF #3 in room U60_south		0	testing room ILLUMINATED (fluorescent lights ON)	14
						4	OF #4 in room		0	testing room ILLUMINATED (fluorescent lights ON)	13

							U60_north			
2	large Open Field, 160x160x60 cm table with a 5 cm high Plexiglas border at edges	208	1	objects in arena locales 10 14 5 and 8	208	1	OF #1 in room U59_south	0	testing room ILLUMINATED (fluorescent lights ON)	104
						2	OF #2 in room U59_north	0	testing room ILLUMINATED (fluorescent lights ON)	104
3	circular large Open Field, 220 cm in diameter and with a 50 cm high wall	140	0	no objects in arena	140	5	Circular arena in room EPM_room	0	testing room ILLUMINATED (fluorescent lights ON)	140
9	Plexiglas box, 40x40x35 cm	5	0	no objects in arena	5	9	Activity box in room ActivityMonitorCages_room	0	testing room ILLUMINATED (fluorescent lights ON)	5

Table 3. Video recording systems (annotated under "exp_scanner") and the number of trials each APPARATUS was video recorded with the indicated system

Video recording system	Open field (OF) # and Central Animal Facility room address		# of Trials
exp_scanner	APPARATUS_ArenaID_value	APPARATUS_ArenaID_valueLabel	
Stationary overhead B&W CCD camera (Ikegami ICD-47) interfaced with a vcr recorder. VHS videotape converted offline to MPEG file using a realtime hardware MPEG encoder (Canopus MPEGPRO EMR/MVR)	1	OF #1 in room U59_south	4893
Stationary overhead B&W CCD camera (Panasonic WV-BP334) interfaced with a vcr recorder. VHS videotape converted offline to MPEG file using a realtime hardware MPEG encoder (Canopus MPEGPRO EMR/MVR)	2	OF #2 in room U59_north	4575
	3	OF #3 in room U60_south	4144
	4	OF #4 in room U60_north	4080
	5	Circular arena in room EPM_room	140
	9	Activity box in room ActivityMonitorCages_room	5
**Camera-vcr recording system Total**			**17837**
Stationary overhead B&W CCD camera (Ikegami ICD-47) connected to a realtime hardware MPEG encoder (Canopus MPEGPRO EMR) and Windows XP computer	1	OF #1 in room U59_south	550
Stationary overhead B&W CCD camera (Panasonic WV-BP334) connected to a realtime hardware MPEG encoder (Canopus MPEGPRO EMR) and Windows XP computer	2	OF #2 in room U59_north	553
Stationary overhead B&W CCD camera (Panasonic WV-BP334) connected to a realtime hardware MPEG encoder (Canopus MPEGPRO MVR) and Windows XP computer	3	OF #3 in room U60_south	522
	4	OF #4 in room U60_north	514
**Camera-computer recording system Total**			**2139**

Table 4. Drug treatments and number of trials with indicated treatment. Co-injected saline is not considered under Drug treatment (TREATMENT_DrugRx_NameOfDrugs) but is listed under Substances injected (TREATMENT_DrugRx_SubstancesAndDosesInjected). Numeral after name of drug is the dose in mg/kg.

Drug treatment<br>TREATMENT_DrugRx_NameOfDrugs	Substances injected<br>TREATMENT_DrugRx_SubstancesAndDosesInjected	# of Trials
CP809101	CP809101 7.5 and SAL 0	6
**CP809101 Total**		**6**
DPAT	DPAT 0.03	144
	DPAT 0.03 and SAL 0 at 24 and 10 and 1 hr before trial	12
	DPAT 0.03125 and SAL 0	147
	DPAT 0.0625	312
	DPAT 0.0625 and SAL 0	215
	DPAT 0.1 and SAL 0	139
	DPAT 0.125	154
	DPAT 0.125 and SAL 0	147
	DPAT 0.125 and SAL 0 at 24 and 10 and 1 hr before trial	12
	DPAT 0.25	144
	DPAT 0.25 and SAL 0 at 24 and 10 and 1 hr before trial	12
	DPAT 1	309
	DPAT 1 and SAL 0 at 24 and 10 and 1 hr before trial	28
	SAL 0[1]	1
**DPAT Total**		**1776**
DPAT+ES	DPAT 0.03 and escitalopram 10 at 24 and 10 and 1 hr before trial	12
	DPAT 0.125 and escitalopram 10 at 24 and 10 and 1 hr before trial	12
	DPAT 0.25 and escitalopram 10 at 24 and 10 and 1 hr before trial	12
	DPAT 1 and escitalopram 10 at 24 and 10 and 1 hr before trial	28
**DPAT+ES Total**		**64**
escitalopram	escitalopram 10 at 24 and 10 and 1 hr before trial and SAL 0	34
**escitalopram Total**		**34**
imipramine	imipramine 10 at 24 and 10 and 1 hr before trial and SAL 0	12
**imipramine Total**		**12**
mCPP	mCPP 0.3125	124
	mCPP 0.3125 and SAL 0	7
	mCPP 0.625	547
	mCPP 0.625 and SAL 0	7
	mCPP 1.25	124
	mCPP 1.25 and SAL 0	7
	mCPP 1.25 and SAL 0 and SAL 0	14
**mCPP Total**		**830**
mCPP+ritanserin	mCPP 1.25 and ritanserin 1 and SAL 0	7
	mCPP 1.25 and ritanserin 5 and SAL 0	6

**mCPP+ritanserin Total**		**13**
QNP	QNP 0.0312	204
	QNP 0.0625	191
	QNP 0.0625 and SAL 0	69
	QNP 0.1	12
	QNP 0.1 and SAL 0	139
	QNP 0.125	552
	QNP 0.125 and SAL 0	726
	QNP 0.125 and SAL 0 and SAL 0	14
	QNP 0.2	392
	QNP 0.2 and SAL 0 at 24 and 10 and 1 hr before trial	36
	QNP 0.25	346
	QNP 0.25 and SAL 0	188
	QNP 0.5	4982
	QNP 0.5 and SAL 0	161
**QNP Total**		**8012**
QNP+clorgyline	QNP 0.5 and clorgyline 1	48
**QNP+clorgyline Total**		**48**
QNP+CP809101	QNP 0.5 and CP809101 7.5	10
**QNP+CP809101 Total**		**10**
QNP+DPAT	QNP 0.0625 and DPAT 0.0625	68
	QNP 0.125 and DPAT 0.03125	147
	QNP 0.125 and DPAT 0.0625	147
	QNP 0.125 and DPAT 0.125	146
	QNP 0.2 and DPAT 1	24
**QNP+DPAT Total**		**532**
QNP+ES	QNP 0.2 and escitalopram 10 at 24 and 10 and 1 hr before trial	24
**QNP+ES Total**		**24**
QNP+IM	QNP 0.2 and imipramine 10 at 24 and 10 and 1 hr before trial	12
**QNP+IM Total**		**12**
QNP+mCPP	QNP 0.125 and mCPP 0.3125	13
	QNP 0.125 and mCPP 0.625	202
	QNP 0.125 and mCPP 1.25	192
	QNP 0.125 and mCPP 1.25 and SAL 0	13
	QNP 0.25 and mCPP 0.625	144
	QNP 0.25 and mCPP 1.25	146
	QNP 0.5 and mCPP 0.3125	7
	QNP 0.5 and mCPP 0.625	7
	QNP 0.5 and mCPP 1.25	7
**QNP+mCPP Total**		**731**
QNP+mCPP+ritanserin	QNP 0.125 and mCPP 1.25 and ritanserin 1	7

	QNP 0.125 and mCPP 1.25 and ritanserin 5	7
**QNP+mCPP+ritanserin Total**		**14**
QNP+ritanserin	QNP 0.125 and ritanserin 1 and SAL 0	7
	QNP 0.125 and ritanserin 5 and SAL 0	7
**QNP+ritanserin Total**		**14**
QNP+U69593	QNP 0.5 and U69593 0.3	140
**QNP+U69593 Total**		**140**
ritanserin	ritanserin 1 and SAL 0 and SAL 0	7
	ritanserin 5 and SAL 0 and SAL 0	6
**ritanserin Total**		**13**
SAL	DPAT 1[1]	1
	SAL 0	6461
	SAL 0 and SAL 0	856
	SAL 0 and SAL 0 and VEHb 0	12
	SAL 0 and SAL 0 at 24 and 10 and 1 hr before trial	46
	SAL 0 and VEHa 0	175
**SAL Total**		**7551**
U69593	U69593 0.3 and SAL 0	140
**U69593 Total**		**140**

[1] **Notations under metadata variable TRIAL_ObservationsAndNotes indicate that these substances were not the prescribed drug treatment and were administered in error. Rat Q34QN8O011 on trial Q34QN8O011_05_H_0153_0011888 got saline instead of DPAT and rat Q34QN8O017 on trial Q34QN8O017_05_0_0158_0011957 got DPAT instead of saline**

Table 5. Number of trials with indicated drug treatment on the successive administration of the treatment.

Drug treatment	Drug treatment number														
	1	2	3	4	5	6	7	8	9	10	11	12	13	14	15
CP809101	6	0	0	0	0	0	0	0	0	0	0	0	0	0	0
DPAT	384	192	185	184	107	106	106	107	107	107	79	58	54	0	0
DPAT+ES	64	0	0	0	0	0	0	0	0	0	0	0	0	0	0
escitalopram	34	0	0	0	0	0	0	0	0	0	0	0	0	0	0
imipramine	12	0	0	0	0	0	0	0	0	0	0	0	0	0	0
mCPP	136	122	119	119	95	94	42	42	40	21	0	0	0	0	0
mCPP+ritanserin	13	0	0	0	0	0	0	0	0	0	0	0	0	0	0
QNP	1114	612	636	601	638	602	636	593	638	617	496	401	369	59	0
QNP+clorgyline	6	6	6	6	6	6	5	7	0	0	0	0	0	0	0
QNP+CP809101	10	0	0	0	0	0	0	0	0	0	0	0	0	0	0
QNP+DPAT	72	48	49	49	49	49	48	49	49	49	21	0	0	0	0

QNP+ES	24	0	0	0	0	0	0	0	0	0	0	0	0	0	0
QNP+IM	12	0	0	0	0	0	0	0	0	0	0	0	0	0	0
QNP+mCPP	130	81	73	77	67	53	55	56	56	55	28	0	0	0	0
QNP+mCPP+ritanserin	14	0	0	0	0	0	0	0	0	0	0	0	0	0	0
QNP+ritanserin	14	0	0	0	0	0	0	0	0	0	0	0	0	0	0
QNP+U69593	10	10	10	10	10	10	10	10	10	10	10	10	10	10	0
ritanserin	13	0	0	0	0	0	0	0	0	0	0	0	0	0	0
SAL	1200	593	633	592	581	541	579	540	574	580	420	329	289	82	18
U69593	10	10	10	10	10	10	10	10	10	10	10	10	10	10	0

Table 6. Number of trials with different brain treatments and number of trials with treatment outcomes.

Brain Treatment<br>TREATMENT_BrainSurgery_manipulation_valueLabel	Success of Brain Treatment<br>TREATMENT_BrainSurgery_HistologyFindingsOutcome_valueLabel	# of Trials
No surgery done	No lesion present	13649
**No surgery done Total**		**13649**
Sham lesion done	No lesion present	2269
**Sham lesion done Total**		**2269**
Basal Lateral Amygdala (BLA) targeted	Lesion meets criterion of at least 55% of ROI lesioned bilaterally	432
	Lesion does NOT meet criteria	268
	No lesion present	14
**Basal Lateral Amygdala (BLA) targeted Total**		**714**
Infralimbic Ctx (ILC) targeted	Lesion meets criterion of at least 55% of ROI lesioned bilaterally	114
	Lesion does NOT meet criteria	66
	No lesion present	224
**Infralimbic Ctx (ILC) targeted Total**		**404**
Nucleus Accumbens Core (NAc) targeted	Lesion meets criterion of at least 55% of ROI lesioned bilaterally	805
	Lesion does NOT meet criteria	925
	No lesion present	378
	Histology not available	27
**Nucleus Accumbens Core (NAc) targeted Total**		**2135**
Orbital Frontal Ctx (OFC) targeted	Lesion meets criterion of at least 55% of ROI lesioned bilaterally	405
	Lesion does NOT meet criteria	208
	No lesion present	72
	Histology not available	48
**Orbital Frontal Ctx (OFC) targeted Total**		**733**
Pituitary targeted	Complete hypophysectomy	72
**Pituitary targeted Total**		**72**

Table 7. Number of trials with available raw data objects

Raw Data Objects Available from Trial		# of Trials
TRIAL_RAWDATAObjectsAvailable_value	TRIAL_RAWDATAObjectsAvailable_valueLabel	
1	Video+Trackfile+Pathplot	18436
2	Trackfile+Pathplot	910
3	Video+Trackfile	554
4	Video+Pathplot	1
5	Trackfile	18
6	Video	57

FIGURE CAPTIONS

Figure 1. Transformation in the spatiotemporal pattern of locomotion induced by repeated injections of quinpirole. The left and right images show the routes of locomotion after 10 injections of saline and quinpirole, respectively. Trajectories during the entire 55 min session are shown, and each line represents a trajectory of locomotion. Green squares indicate locations of four objects in the open field. Note that the quinpirole-treated rat moved much more than the saline animal, and that the spatial distribution of locomotion in the quinpirole rat was much more restricted compared to the saline rat, which explored and moved all around the open field.

Figure 2. Photographs of the standard open field and the four objects at their regular locales.

Figure 3. Open field virtual grid and location of 4 objects in it. Top row shows the position of 4 objects (green squares) in the open field during regular trials (left) and on the object rotation test (right); bottom row shows trajectories of locomotion on those two trials by a rat treated with quinpirole. Note the shift in the routes of locomotion on the object rotation test, towards the new position of the objects. The green squares in the bottom right image are not at the objects' new position but at their regular trial locations. Numbers in the virtual grid are the names of the 25 locales in the open field.

Figure 4. Photographs of experimental set up in studies using an animal model of OCD. Top row shows photographs of the ceiling-mounted video camera focused on the open field, fluorescent lighting illuminating the testing room, and a vcr-monitor station standing on shelves attached to the wall. Middle row, left photo, shows the partially drawn curtain between the open field and the vcr-monitor station; right photo is a screen capture image from a video file showing the experimenter placing a rat onto the open field. Bottom photo is of the workbench located between the two curtains separating the open fields. On the workbench are two home cages with a rat inside. Experimenter prepared syringes with the drug solution, administered the drug treatment, and placed the rat on the open field. Experimenter was present in the testing room throughout the trial, but was hidden behind a curtain from the animal's sight. Experimenter observed activity of the rat on a TV monitor and retrieved the rat if it fell or jumped off the arena. The rat was generally placed back at the spot from which it left the arena. Montage is of photos taken at different times, from 2004 to 2010.

Figure 5. Photographs of the method used in calibration of the open field. Top photo is of the camera view of the open field with black markers positioned every 40 cm. Such a video image was obtained for each open field and before the start of every study any time adjustments were made in the experimental set-up. Bottom photo is a screen capture of the Advanced Calibration tab in EthoVision. The coordinate values of the black markings constitute the spatial calibration of the open field and are used by the program to assign the XY coordinates of rat locomotion.

FOOTNOTES

[a] Metadata variables constructed by the authors to annotate the data objects from studies using the animal model are indicated in bold font. Metadata variables from GigaDB vocabulary are indicated in quotation marks.

Click here to access/download;Figure;Figure1_szechtman.pdf

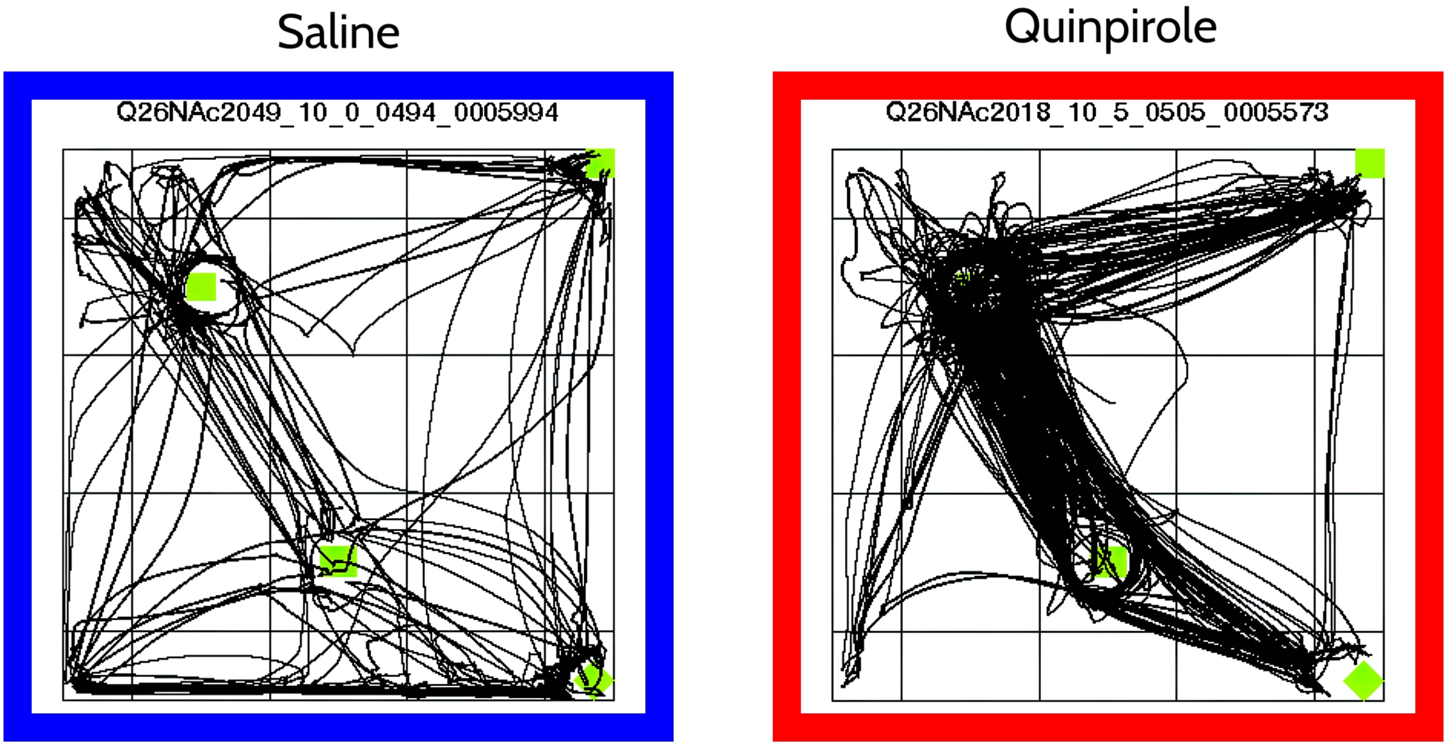

Figure 2 pdf

Click here to access/download;Figure;Figure2_szechtman.pdf

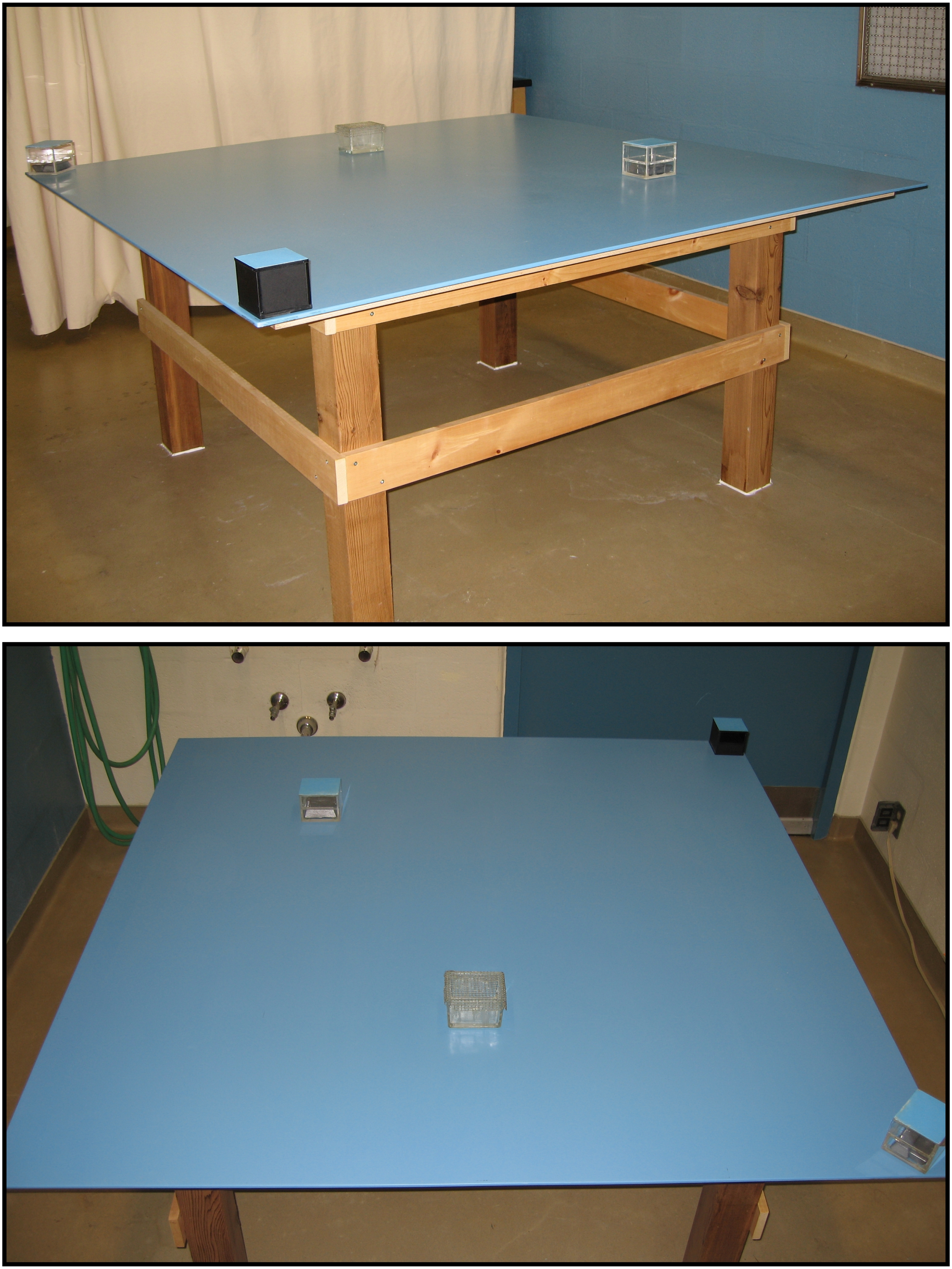

Figure 3 pdf

Click here to access/download;Figure;Figure3_szechtman.pdf

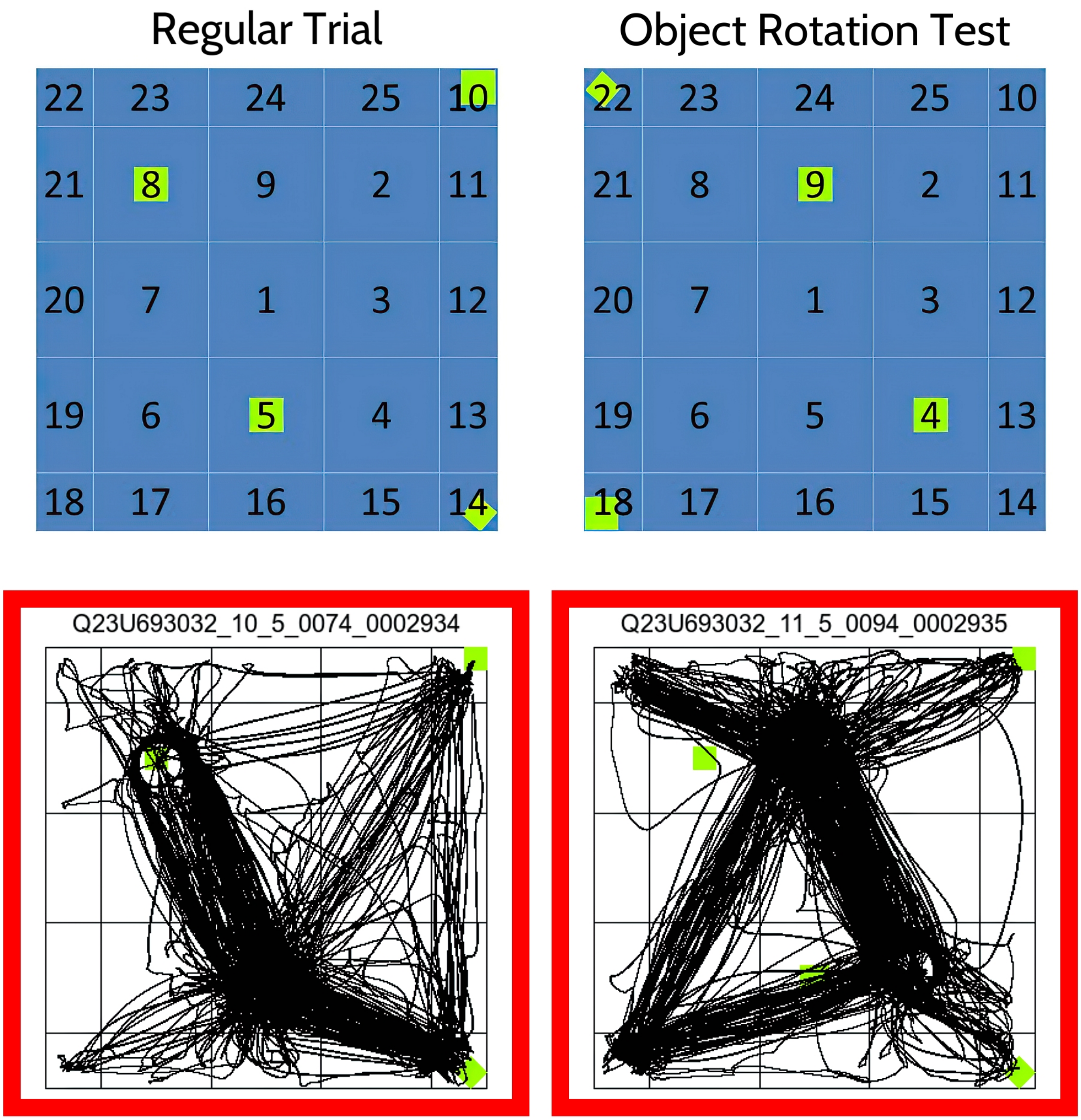

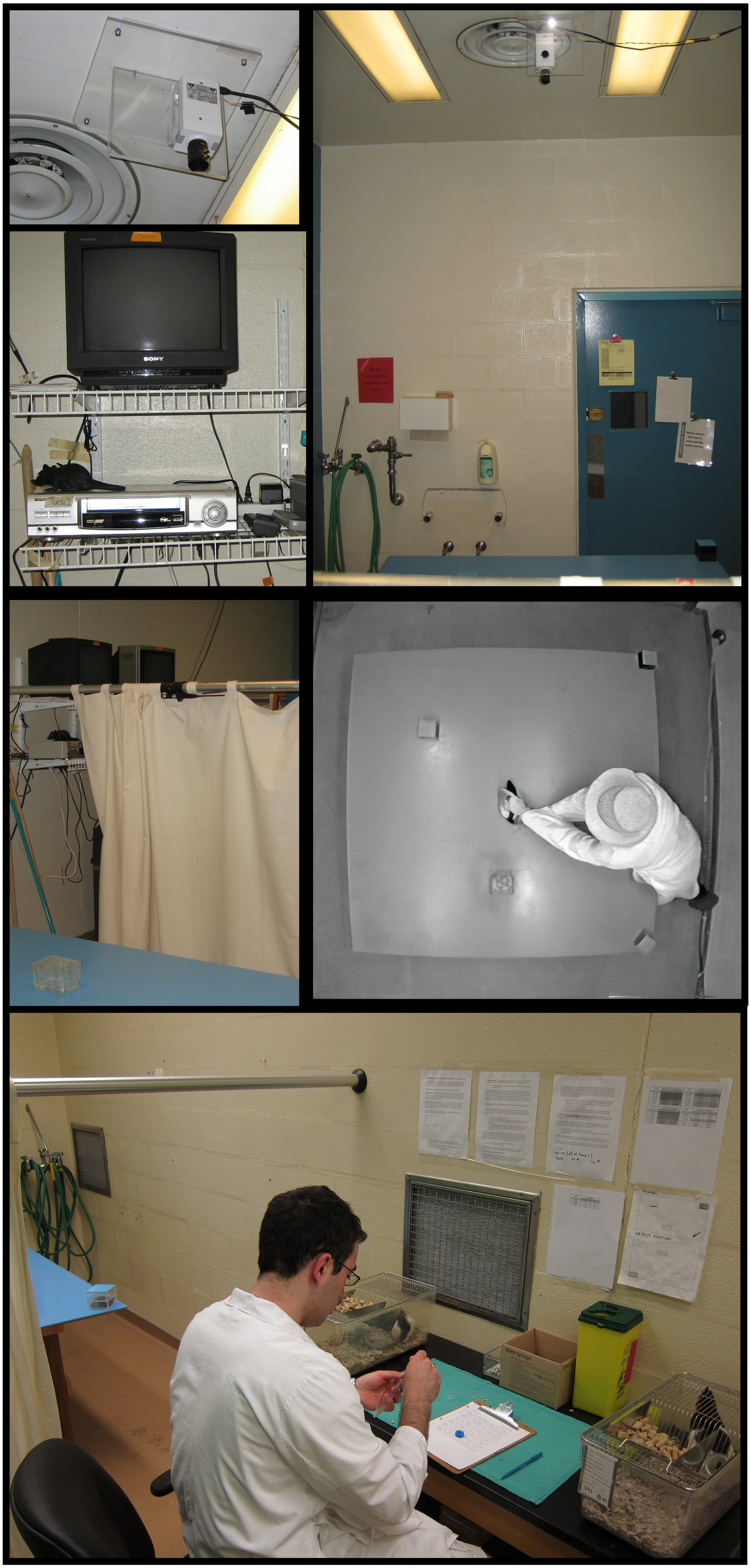

 Click here to access/download;Figure;Figure5_szechtman.pdf

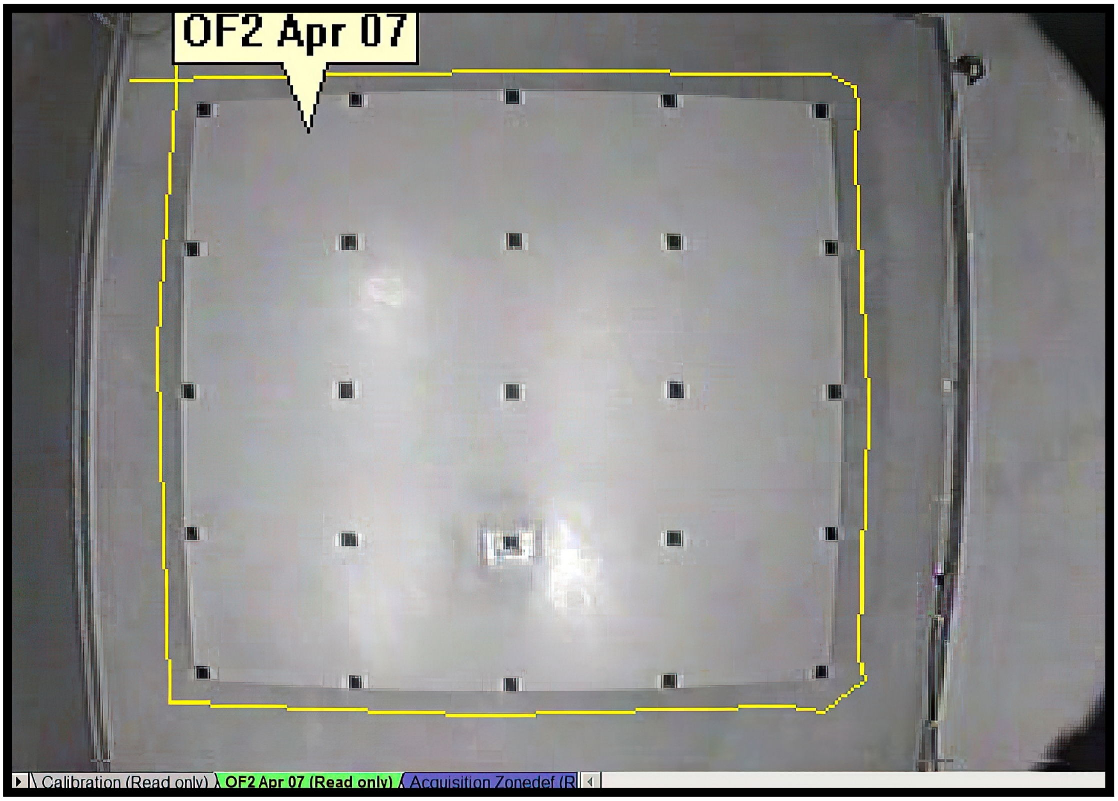

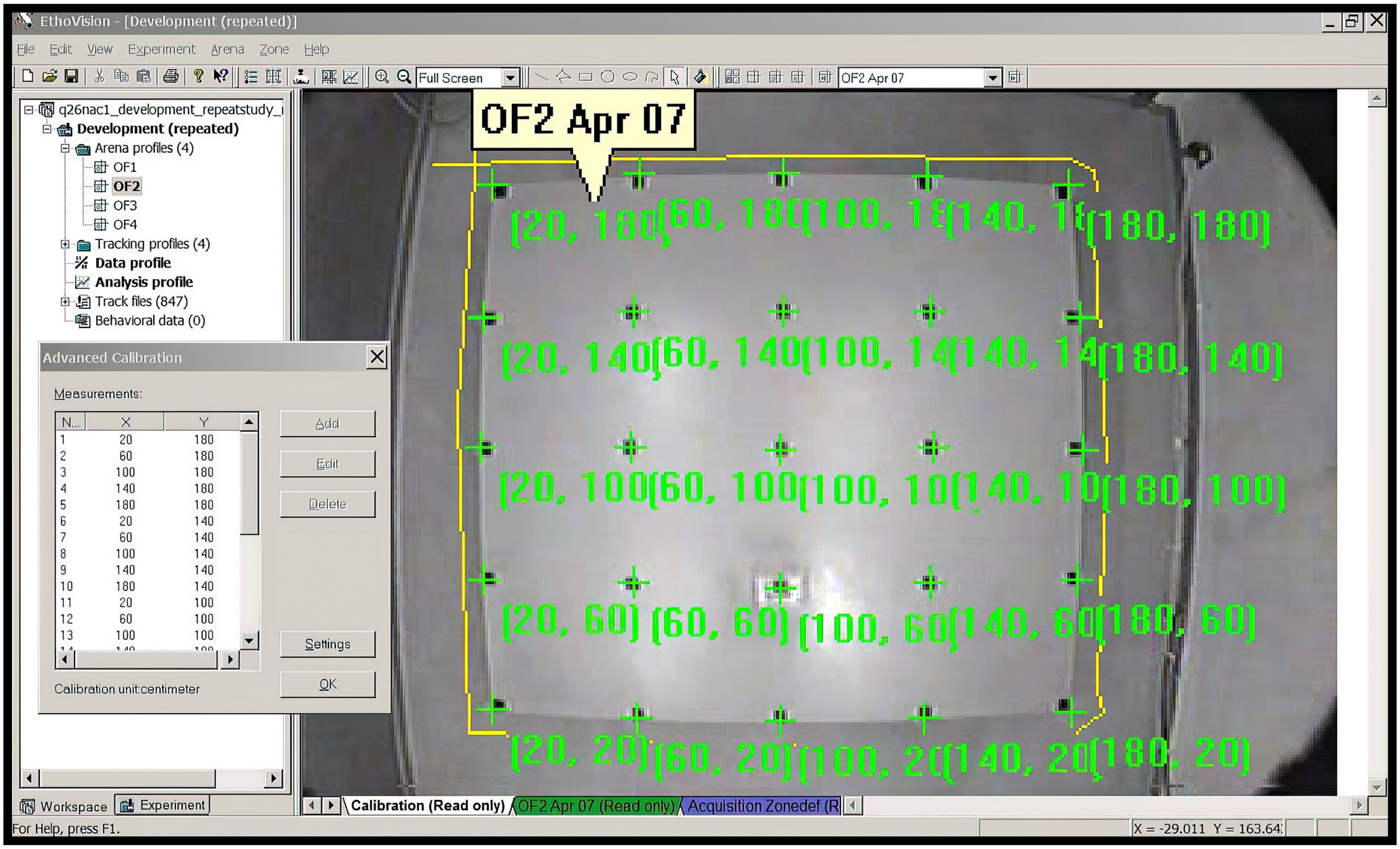

Supplementary Material 1

Click here to access/download

Supplementary Material

GigaDB_SampleAndFilesTabs_20220420_SupplementaryMaterial.xlsx

Supplementary Material 2

Click here to access/download
Supplementary Material
FRDR_interface_20211029_SupplementaryMaterial.xlsx

Supplementary Material 3

Click here to access/download

Supplementary Material

GigaDB_MetaDataTable_20220421_SupplementaryMaterial.xlsx
