# Supplementary material for: A virtual library for behavioral performance in standard conditions—rodent spontaneous activity in an open field during repeated testing and after treatment with drugs or brain lesions
Source: Gigascience. 2022 Oct 20;11:giac092. doi: 10.1093/gigascience/giac092 (PMC9581716; doi:10.1093/gigascience/giac092)
Supplement: giac092_Response_to_Reviewer_Comments_Original_Submission [file giac092_response_to_reviewer_comments_original_submission.pdf]

Author's Response To Reviewer Comments

Close

Dear Dr Nogoy
We thank you and the two reviewers for their positive and helpful comments on our manuscript. Below we respond to the reviewers' comments and indicate how we revised the manuscript.

Reviewer 1
Reviewer was overall positive about the submission but had 4 areas of concern:
1. Reviewer did not believe that the Introduction was sufficiently comprehensive in putting our database into the context of other available datasets. Furthermore, we believe the Reviewer was concerned that our lack of comprehensiveness could be interpreted by readers that video datasets of behavior do not exist and ours is the first one, or completely unique.
a. We apologize if the Introduction gave the impression that ours is the only video dataset of behavior. We certainly did not intend to suggest this. The references cited in the first paragraph of the Introduction point to the breadth of video analysis tools and explicitly or by implication to video datasets. In the revised manuscript, we added several more references to the list of video analysis tools/datasets that we became aware of, following the Reviewer's critique. We did not add a citation to OpenMonkeyStudio as this tool (using 64 cameras) seems to be in a league way above our video recordings.
2. Reviewer suggests we should be "more specific about impacts and intended use cases", by describing in greater detail the type of analyses that should be performed on the dataset.
a. We added to the section "Re-use Potential" (p. 25) a few more examples of the type of questions that could be explored using the deposited digital library. However, we do not go into detail because the purpose of the submitted Data Note is quite focused—describe in detail the properties of a database that has re-use potential. Unlike a GigaScience Research Report, a Data Note is not intended to demonstrate novel findings obtained by some re-analysis of the database.
3. Reviewer suggests that "sample data analysis scripts would elevate the manuscript's impact".
a. We considered following this suggestion and including the Mathematica scripts we employed in our workflow. However, because of dependencies of those scripts on the SEE Workshop, the task became onerous since the Mathematica scripts would need modifications and this would be a project in itself. Similarly, the Reviewer's suggestion that we provide "some simple analysis scripts" in python, is not something that we have available or can do for inclusion with this Data Note.
4. Reviewer suggests that the readability of the paper would be enhanced by converting some of the tables to figures, e.g., Table 4 to a series of bar graphs.
a. We looked whether the reader would be served better by converting the tables (including Table 4) to a series of graphs. We do not believe so. Tables provide lots of detail information, which is not easily visible with graphs; graphs are best for spotting trends with a glance, not for conveying details.

Reviewer 2
We are grateful to the Reviewer for the detailed summary of the manuscript content. Grateful—because the summary makes clear that the manuscript achieved its intended goal of describing the properties of a database that has re-use potential. Nevertheless, the Reviewer stated several specific concerns, to which we responded in the revised manuscript as follows:

I. Reviewer highlights that our "annotation schema is well constructed" and could be taken as an example to organize "own data". Thank you for this feedback!
II. In points ii.and iii, Reviewer comments that the manuscript does not mention limitations on re-use potential such as image resolution and effective size. Moreover, the Reviewer indicates that there is no clear information as to the effective size of available data objects meeting more than two conditions.
a. On pages 25-26 we added a section called Limitations on Re-use Potential where we address the points raised by the Reviewer, including how to obtain the count of available data objects meeting multiple criteria. We trust that this section is satisfactory.
b. Although in the manuscript we did not elaborate on it, it seems that in terms of video resolution the issue is not the dimensions of the image per se but rather how much of the image is occupied by the object of interest. For example, in the paper on whisker analysis cited in subsection Video resolution,

video resolution is also 352x240 pixels but almost the entire head of the mouse occupies the video frame.
III. This comment is a continuation of point ii addressed above.
IV. As we understand it, Reviewer seems to suggest that because activity in the open field is affected by environmental structure this limits the re-use potential of the database to those interested in OCD.
a. We don't fully understand the above comment and the implication of database usefulness limited OCD research. Spontaneous behavior in general is subject to environmental constraints, and the empirical question is to what degree this is the case in specified situations, a question that could be addressed using the library since the conditions are well specified. Moreover, not all treatments yield OCD behavior; e.g., saline injections. We did not respond to point iv in the revision of the manuscript.
V. Reviewer indicates that the Data Note lacks "scientific novelty" but this would be remedied if the manuscript included results from some analysis using the library to address "new questions".
a. As noted in the response to Reviewer 1 (point 2) we added a few more examples of interesting questions that could be addressed in re-using the database. We do not provide results from any potential analysis since this is not a requirement of a Data Note publication, and was not our intended objective. Rather, our scientific objective was to "describe in detail the properties of a database that has re-use potential", as noted above in response to Reviewer 1.
VI. Reviewer notes that "even the database itself is not novel because at least partly is "available in publications of the original studies and in the FRDR".
a. On top of page 6, we indicated that much of the pertinent information describing the raw data objects was collated from our published reports, which may have led the Reviewer to infer that together with those publications we deposited the underlying raw data objects. However, this is not the case. Consequently, we appended the following sentence to the end of the paragraph: "Note that those reports were not accompanied with the publication of datasets containing the raw data objects described in the present Data Note paper."
b. It is the case that the raw data objects were deposited in FRDR and the submitted Data Note manuscript indicates that those data objects would be mirrored in the GigaDB database. However, the latter is no longer the case (see below), so there is only one deposit of the raw data objects, and that is in the FRDR repository. Importantly, what is new and novel is the Data Note report, which provides in a publication form a detailed exposition of the annotation schema, the associated metadata, the re-use potential of the database, and the suggestion that our library could be used as a seed towards an effort to develop a schema and a platform which would allow the construction of a "machine searchable virtual library of behavioral performance in defined conditions".
VII. Reviewer suggests we would make a more significant scientific contribution if we developed our "elegant schema further and transform it into an indexing tool that organizes broader datasets, already available in different repositories".
a. Alas, what the Reviewer suggests is correct. When preparing to put our data in a repository I looked for templates how to annotate the raw data objects, but did not find a suitable one. Further inquiries on metadata annotations made it abundantly clear that this is a complex issue, beyond my current skill set, and hence came out with the annotation schema described here. Nevertheless, as stated in the last paragraph of the Re-use Potential section: "The library may have yet another utility, a less obvious one but potentially impactful. As raised in the Introduction, the underlying motivation in making the raw data objects publically available is to ultimately create a machine searchable virtual library of behavioral performance in defined conditions. The approach used here in annotations of the raw data objects is a step towards this goal. However, to achieve the goal of searchable linked open datasets requires a more sophisticated and formal schema [68-70]. The library described here could be of value both as the seed and test bed in this endeavor."

Other changes in the revised manuscript
After the manuscript was submitted, the journal editors re-evaluated their original suggestion to mirror in the GigaScience Database the raw data objects available in the FRDR repository. Instead, it was proposed that the metadata annotations be deposited in GigaDB and include download links to the files in FRDR. Consequently, we changed the pertinent text in the revised manuscript to reflect this state.
In addition, the revised manuscript underwent some minor edits, including the addition of a few references.

We thank you for your efforts to make our report a first rate publication – we truly appreciate this. We hope you find this revision suitable and we look forward to hearing from you.

Sincerely yours,
Henry Szechtman, PhD
Professor Emeritus

Close
