# Supplementary material for: A virtual library for behavioral performance in standard conditions—rodent spontaneous activity in an open field during repeated testing and after treatment with drugs or brain lesions
Source: Gigascience. 2022 Oct 20;11:giac092. doi: 10.1093/gigascience/giac092 (PMC9581716; doi:10.1093/gigascience/giac092)
Supplement: giac092_Response_to_Reviewer_Comments_Revision_1 [file giac092_response_to_reviewer_comments_revision_1.pdf]

Author's Response To Reviewer Comments

Close

Dear Dr Nogoy
We are glad and thank the reviewers for finding our revisions satisfactory. However, Reviewer 2 expressed concerns regarding our response to the issue of low video resolution, especially how it pertains to the feasibility of tracking kinematics and body postures using currently available software systems. We indicated that the issue of feasibility in tracking body parts is "an empirical question" but Reviewer 2 interpreted our response as being "dismissive" of the stated concern. Reviewer 2 indicated that "advertising a potential involves the responsibility of demonstrating its feasibility"; specifically, that we should provide a "concrete estimation" of this feasibility. Furthermore, Reviewer 2 volunteered that this is "so easily achievable (for example, training with DeepLabCut, which is open source and beautifully documented)".

We thank the Reviewer for spotting that our language may have slipped from descriptive into "advertising" – we have corrected this slippage in the revised manuscript. We have also explained why it is not "easy" for us to do the suggested examination – I no longer have an active research lab. (BTW, I know from years of experience that "nothing difficult is ever easy"). The revised paragraphs in the "Video resolution" section read as follows:
"Our video recordings are a top-down view of a large open field (1.6 x 1.6 m) and of a (relatively small) rat within it. Clearly, the 352x240 pixel image size was more than adequate to track whole body locomotion, and the same image size could be used even to analyze small movements of body parts such as whiskers (e.g., [72]), when the entire field is occupied by the region of interest—the nose and vibrissae of the mouse. . However, importantly, is the image size of our videos good enough to resolve in the (relatively small) rat the kinematics and body postures tracked by several modern video analysis systems cited in the Introduction? This is an empirical question to which unfortunately we are not in a position to provide a satisfactory answer since this would require tracking our videos with those software platforms, but the principal investigator cannot undertake such a task given that he no longer has an active research laboratory. We can only offer the speculation that even though those video analysis systems were likely developed using higher resolution images, their method could work nevertheless on a lower resolution video or be adapted to work on it. This is not unlike the situation for the methods in molecular biology which were originally developed to analyze living tissue under optimum conditions but which can be employed now to examine archeological samples.
However, should video resolution indeed be a problem, conceivably, we speculate, such a limitation could be remedied with software to enhance and upscale videos (e.g., Topaz Video Enhance AI; [73]), an option which an interested user could explore."

In the revised manuscript, we also tweaked the text in the Re-use Potential section to de-emphasize the analysis of body parts, as from the reviewer's comments it would seem that we consider that this would be the only/essential re-use potential. The revised sentence is:
"For example, the library could be used to train neural networks to identify and measure ritual behavior of relevance to OCD (along the lines scored manually in [27]); or to classify effects of different drugs on behavioral activity; or, perhaps, to track body parts and extract postural kinematics."

Finally, we added reference 71 in the sentence "However, to achieve the goal of searchable linked open datasets requires a more sophisticated and formal schema" in the last paragraph of section Re-use Potential; this reference should have been included in the original draft.

We hope that this revision is suitable and we look forward to hearing from you.

Sincerely yours,
Henry Szechtman, PhD
Professor Emeritus

Close
