# Supplementary material for: A virtual library for behavioral performance in standard conditions—rodent spontaneous activity in an open field during repeated testing and after treatment with drugs or brain lesions
Source: Gigascience. 2022 Oct 20;11:giac092. doi: 10.1093/gigascience/giac092 (PMC9581716; doi:10.1093/gigascience/giac092)
Supplement: giac092_Response_to_Reviewer_Comments_Revision_2 [file giac092_response_to_reviewer_comments_revision_2.pdf]

Author's Response To Reviewer Comments

Close

Dear Dr Nogoy
I made all the changes as requested. For the Funding section, I moved the last paragraph from Acknowledgements to Funding.
With many thanks for the consideration and review of our manuscript
Henry Szechtman

Close
