# Supplementary material for: A virtual library for behavioral performance in standard conditions—rodent spontaneous activity in an open field during repeated testing and after treatment with drugs or brain lesions
Source: Gigascience. 2022 Oct 20;11:giac092. doi: 10.1093/gigascience/giac092 (PMC9581716; doi:10.1093/gigascience/giac092)
Supplement: giac092_Reviewer_1_Report_Original_Submission — Jesse Marshall -- 6/4/2022 Reviewed [file giac092_reviewer_1_report_original_submission.pdf]

Reviewer Report

Title: A Virtual Library for Behavioral Performance in Standard Conditions â€“ Rodent Spontaneous Activity in an Open Field during Repeated Testing and after Treatment with Drugs or Brain Lesions

Version: Original Submission **Date:** 6/4/2022

Reviewer name: Jesse Marshall

Reviewer Comments to Author:

The manuscript presents a large, 11 TB database of ~20,000 55 minute video recordings of rats behaving in an open field arena over the course of fifteen years. The rats are given various drugs and brain lesions, and some of the experiments have many repeated trials per animal. The general focus of the experiments is investigations of a quinpirole-induced model of OCD> In the recordings the XY center of mass of animals were tracked using EthoVision. These recordings are subdivided into 43 separate sub-experiments. The large data corpus is being hosted by GigaDB. The manuscript painstakingly describes the experimental design, video recording conditions and metadata for these experiments. This includes experiments organization into different projects, the types of drugs and brain lesions administered to animals, and the number of repeated trials for animals. This metadata is stored as excel files.
Overall I am enthusiastic about the idea of this manuscript and the assembly of large corpuses of animal behavior. That said, the bulk of the present manuscript is focused on low-level explication of properties of the database, rather than comparing itself with other behavioral databases or more specific potential impacts this manuscript could have. The stated purpose is rather vague and I think low hanging fruit can be identified. The number of figures is also lacking and I think could make the work more digestible. I think this work could have benefit to the field, but needs to do a bit more work contextualizing itself within the modern framework, and the manuscript can be streamlined to be of more interest.
Major Points
1) Contextualization within the field. Other animal behavioral datasets exist, mostly focused around pose estimation, eg OpenMonkeyStudio or the Multi-animal behavior estimation challenge datasets. These also touch on applications for large datasets, eg through self-supervised learning. Other labs have large datasets (eg the schaevitz lab/Gordon berman and likely others) that may or may not be publicly released. This dataset is much larger, it has clear value, but the specific value should be made more clear.
2) Being more specific about impacts and intended use cases. What are the discoveries that can be made? What are the first things that should be tried? The states uses are fairly vague, and don't reference the specific types of drug data and experiments included here. For the amount of description given to describing the metadata, this needs to be more concrete.
3) Sample data analysis scripts would elevate the manuscript's impact. The biggest risk of this database is that it doesn't get used. The authors make many steps to addressing this by having well documented metadata. However without some code entry points, researchers may not want to spend the time invested in using this data. Some simple analysis scripts that show e.g. how to load the center of mass tracks into python, filter metadata table using pandas dataframes, and aggregate statistics (e.g. compare

average velocity of animals on saline to quinpirole) would go a long way to ensuring the datasets reuse. 4) Additional figures, and potential metaanalyses. The manuscript is heavy on tables and description of the data and light on summary plots. Table 4 for instance could just be a series of bar graphs? This would raise the readability and interest considerably.

Level of Interest

Please indicate how interesting you found the manuscript: Choose an item.

Quality of Written English

Please indicate the quality of language in the manuscript: Choose an item.

Declaration of Competing Interests

Please complete a declaration of competing interests, considering the following questions:

- Have you in the past five years received reimbursements, fees, funding, or salary from an organisation that may in any way gain or lose financially from the publication of this manuscript, either now or in the future?
- Do you hold any stocks or shares in an organisation that may in any way gain or lose financially from the publication of this manuscript, either now or in the future?
- Do you hold or are you currently applying for any patents relating to the content of the manuscript?
- Have you received reimbursements, fees, funding, or salary from an organization that holds or has applied for patents relating to the content of the manuscript?
- Do you have any other financial competing interests?
- Do you have any non-financial competing interests in relation to this paper?

If you can answer no to all of the above, write 'I declare that I have no competing interests' below. If your reply is yes to any, please give details below.

No

I agree to the open peer review policy of the journal. I understand that my name will be included on my report to the authors and, if the manuscript is accepted for publication, my named report including any attachments I upload will be posted on the website along with the authors' responses. I agree for my report to be made available under an Open Access Creative Commons CC-BY license (http://creativecommons.org/licenses/by/4.0/). I understand that any comments which I do not wish to be included in my named report can be included as confidential comments to the editors, which will not be published.

Choose an item.

To further support our reviewers, we have joined with Publons, where you can gain additional credit to further highlight your hard work (see: https://publons.com/journal/530/gigascience). On publication of this paper, your review will be automatically added to Publons, you can then choose whether or not to claim your Publons credit. I understand this statement.

Yes Choose an item.
