# Supplementary material for: A virtual library for behavioral performance in standard conditions—rodent spontaneous activity in an open field during repeated testing and after treatment with drugs or brain lesions
Source: Gigascience. 2022 Oct 20;11:giac092. doi: 10.1093/gigascience/giac092 (PMC9581716; doi:10.1093/gigascience/giac092)
Supplement: giac092_Reviewer_2_Report_Original_Submission — Inbar Saraf-Sinik -- 6/18/2022 Reviewed [file giac092_reviewer_2_report_original_submission.pdf]

Reviewer Report

Title: A Virtual Library for Behavioral Performance in Standard Conditions â€“ Rodent Spontaneous Activity in an Open Field during Repeated Testing and after Treatment with Drugs or Brain Lesions

Version: Original Submission **Date:** 6/18/2022

Reviewer name: Inbar Saraf-Sinik

Reviewer Comments to Author:

A Virtual Library for Behavioral Performance in Standard Conditions - Rodent Spontaneous Activity in an Open Field during Repeated Testing and after Treatment with Drugs or Brain Lesions - Review
General
In the manuscript, Szechtman et al. propose an open access repository constructed of behavioral data they have acquired while studying obsessive-compulsive disorder (OCD) in rats as an animal model. The authors suggest that, much like blood or tissue databases, behavioral data, if large enough and well-annotated, can be reused and potentially lead to new discoveries. Specifically, their database includes video (352x240 px, 30 fps), derived X-Y coordinates of center of mass, trajectories and their plots of free locomotion by rats ("raw data objects"), and a structured annotation of metadata information ("general schema") for each raw data record (test).
The authors define two main requirements for a virtual library and elaborate on how their proposed database meets them: 1) it is large, and 2) it is clearly annotated in a well-organized and easily searchable structure. These two strong points, the authors claim, endow the database with a high potential for future reuse. Very generally, they explain that the data may be of value for future, yet unknown purposes, mentioning AI-based applications, statistical evaluation of subtle factors, simulation of new experiments, or as an educational resource.
The manuscript then focuses on a detailed description of the structure and specific values of the annotation schema. It is evident that the authors put much thought and effort into the annotation schema. They dedicate most of the manuscript to providing a comprehensive view of its structure and the specific parameters it addresses, including the labels and possible values each parameter can have. Indeed, they produced a detailed, precise, and easily searchable structure that associates individual raw data records (tests) with metadata information. Noteworthy, despite the large amount of annotated data (collected for over 15 years of research), this schema is surprisingly practical. It allows potential future users to easily orientate and select subsets of data deemed relevant for their research. The proposed manuscript is likewise clearly written and well organized, almost like a user manual to allow a starting point for any user.
While commendable, the annotation schema, which the authors describe throughout the manuscript, does not contribute a novel scientific finding. Contrary to GigaScience publications that allow access to the data and also demonstrate novel analyses and results, this manuscript focuses entirely on data access and their mapping. As such, I believe that in its current version, the manuscript does not comply with GigaScience publication.
Yet, the authors make excellent points about the potential contribution of such a virtual library to the

scientific community, and I encourage them to develop this endeavor further. One direction can be to actually reuse their data and present new scientific findings. With years of experience in OCD research and their accumulated data, the authors are in an optimal position to suggest concrete new questions that can use their data. Another intriguing direction is to transform their annotation schema into some indexing library for data produced by the broader scientific community and available in different repositories. Establishing an updating library can significantly facilitate data sharing and reuse for scientific and educational purposes and realize the potential to lead to new findings. Broadening the library reduces the risk of data biases that result from implementing the same experimental protocols or working within the framework of specific fields. In addition, it has the advantage that it can be detached from the actual data repositories, also allowing the inclusion of data already available is existing repositories or as part of the supplementary material of publications. Other than saving space, it also avoids duplications. For example, of the 43 experiments that compose the proposed database, 29 were already deposited in the FRDR. I appreciate that establishing such a tool will require overcoming many technical, scientific, and legal challenges. Still, I believe that it is feasible and even essential that such a resource exists.

Specific comments

The authors state that the potential reuse value of such a virtual library is determined by i) its straightforward and practical organization and ii) its large data size. To their list, I also add iii) the compatibility of data it encompasses with new analyses tools and measurements.

i. The annotation schema is well constructed. It provides detailed metadata information that is associated with large data volume, and yet, it is clearly organized, allowing easy access and selection of relevant portions from the database. It is truly a remarkable achievement by the authors that I hope to take an example from organizing my own data. In that respect, it complies with the authors' objective of producing a virtual library for behavioral data.

ii. The authors assert that the large size of a virtual library determines its high reuse potential. Referring to an overall number of 19,976 tests, each 55 minutes long, from 43 experiments, involving 1758 rats and mounting to over two years of recorded data and about 11 terabytes, the authors emphasize the large size of this database. While I agree with the general statement, I think that to estimate the potential value of such a library, one should address its effective-, rather than overall- size. The effective size, considers the amount of data that would be utilized in practice for the potential types of usage. Two possible approaches can benefit from such a database: a) selective data: phrasing new specific questions directly related to one or few of the experimental designs and conditions implemented in the original studies. Those relevant portions will be selected from the larger database, and b) Non-selective data: phrasing new general questions, which either depend on large variability across the data or can afford to ignore variability originating from the conditions and treatments implemented in the original studies, assuming it will be averaged out. While the overall size of the database may be relevant for the latter, for the specific questions, the effective size is defined by the datasets selected. Following the annotation details of treatment and conditions across the database revealed that it is composed of many subgroups:

1. Brain lesions: in 4 possible brain regions (BLA, NAc, IL, or OFC) with variable success levels as confirmed by histology, shame surgeries, or no surgeries.
2. Pharmacological treatments: 9 different drugs, and combinations of 2 or 3 together, or vehicle (saline

or other) controls. In addition, studies that checked dose dependence
effects introduce more subgroups, for example quinpirole was tested using 7 different dosages.
3. Arenas: the prevalent arenas were 1 of 4 possible elevated tables (160x160 cm), positioned in 1 of 2 possible rooms, one closer to the entrance door and the other farther away.
In some tests, these tables were enclosed by walls. Additional arenas that were used are a large cylindric (140 cm diameter) arena and a small locomotor box (40x40x35 cm).
4. Object arrangements: objects were arranged in consistent positions, rotated in 180 degrees, or absent.
5. Illumination conditions: tests were acquired in the dark (IR illumination) or in visible light. It is not clear if changes of illumination were done while keeping the subjective night/day of the rats or if testing outside the rats' subjective day introduces yet another difference.
6. Housing: rats were housed in standard or enriched home cages (supplementing objects).
7. Experience: rats were repeatedly tested, gaining experience that may affect their behavior in the arena. Moreover, repeated tests sometimes followed different drug administration protocols, making it hard to understand the effect of experience on rats' locomotion trajectories.
While the authors state how many of the tests were acquired in each category and value (e.g., how many tests followed injection of drug X), the overlap between them is not clear (e.g., injected with X AND tested in arena Y AND object arrangement Z…etc.), making it hard to evaluate the effective sizes of specific subgroups in the database.
iii. Data compatibility- similar to the effective size, data compatibility relates to the potential usage. The videos acquired were of a spatial resolution of 352x240 pixels, and captured free locomotion by rats in an area of 160x160 cm (for the most frequently used arena). While sufficient for tracking the center of mass coordinates, this resolution is too low to satisfy more subtle tracking of body parts (key-points), 3D pose estimation, clustering, and the use of statistical models and machine learning tools of the sort the authors cite (references 7-18). These are current, not future, applications utilizing large data repositories, for example, to identify and characterize behavioral motifs and their sequences in freely moving animals. The low spatial resolution poses considerable limitations on the type of analysis and, thus, new measurements that can be extracted from the raw data included in the proposed database.
iv. If the center of mass tracking is sufficient for the general questions approach (e.g., characterizing path or velocity patterns in free locomotion by rats), the reuse potential of this database depends on the degree to which it represents the behavioral repertoire. In this database, the particular properties of the arena (size, shape, objects) significantly affect behavior, effectively narrowing the behavioral repertoire. Re-testing in the same area further converges such patterns, especially following OCD- relevant treatments (specific lesions and drugs implemented across these data). The resulting data biases limit the reuse potential of these data in contexts outside the OCD field.
v. Serving a selective data approach, while possible, it is not likely that questions of this specific type will be addressed by the general scientific community or even outside the lab that produced these data. For the non-selective data approach, these data suffer from resolution-derived limitations on the potential measurements and data biases resulting from a reduction of behavioral repertoire. However, if the authors can demonstrate concrete examples for new questions and reuse their data or parts, such results would satisfy the scientific novelty currently lacking in the manuscript.
vi. Of note, the proposed database is at least partly available in publications of the original studies and in

the FRDR. Thus, even the database itself is not novel.
vii. Ultimately, the manuscript should explicitly demonstrate what exactly is the added value of putting all these data in one large database? How is this database functionally distinct from and advantageous compared to creating an indexing link to already available data in different repositories? Suppose instead (or in addition), the coherent organization structure of data is the main reason, it should correspondingly be the main product of this manuscript. In that case, I encourage the authors to develop their elegant schema further and transform it into an indexing tool that organizes broader datasets, already available in different repositories, including their own data that is still not available for access.

Summary

This manuscript essentially offers access to data acquired for research of OCD in rats as an animal model, and focuses on a detailed description of the metadata organization. The database itself is very impressive in terms of its large volume, as well as organization and consistency. However, the specificity of subgroups within the data dictates a much smaller effective size of datasets than the overall volume, hindering the authors' assertion regarding the high potential reuse stemming from its large overall size. In addition, the low video resolution makes the data subpar compared with current-day analysis tools. Had it been the only way to preserve this impressive body of data produced during a lab's lifetime, I would consider pushing towards publication. However, most of the data are already available in a public repository.

The annotation schema for data organization functionality added here is desirable but does not constitute by itself sufficient advancement to warrant a separate publication. Regardless, in my view, the authors excelled constructing this schema. It is very detailed and informative, organizing a large volume of data, and yet, the authors succeeded in making it clear, practical and easily searchable. I commend their idea, effort and achievement.

I hope that the authors either add novel analyses and present new results and insights using their data or add an extension of the 'library' functionality to include broader datasets; in either case a re-evaluation of such publications would be most welcome.

Level of Interest

Please indicate how interesting you found the manuscript: Choose an item.

Quality of Written English

Please indicate the quality of language in the manuscript: Choose an item.

Declaration of Competing Interests

Please complete a declaration of competing interests, considering the following questions:

- Have you in the past five years received reimbursements, fees, funding, or salary from an organisation that may in any way gain or lose financially from the publication of this manuscript, either now or in the future?
- Do you hold any stocks or shares in an organisation that may in any way gain or lose financially from the publication of this manuscript, either now or in the future?
- Do you hold or are you currently applying for any patents relating to the content of the manuscript?
- Have you received reimbursements, fees, funding, or salary from an organization that holds or has applied for patents relating to the content of the manuscript?
- Do you have any other financial competing interests?
- Do you have any non-financial competing interests in relation to this paper?

If you can answer no to all of the above, write 'I declare that I have no competing interests' below. If your reply is yes to any, please give details below.

No to all of the above

I agree to the open peer review policy of the journal. I understand that my name will be included on my report to the authors and, if the manuscript is accepted for publication, my named report including any attachments I upload will be posted on the website along with the authors' responses. I agree for my report to be made available under an Open Access Creative Commons CC-BY license (http://creativecommons.org/licenses/by/4.0/). I understand that any comments which I do not wish to be included in my named report can be included as confidential comments to the editors, which will not be published.

Choose an item.

To further support our reviewers, we have joined with Publons, where you can gain additional credit to further highlight your hard work (see: https://publons.com/journal/530/gigascience). On publication of this paper, your review will be automatically added to Publons, you can then choose whether or not to claim your Publons credit. I understand this statement.

Yes Choose an item.
