# Supplementary material for: A virtual library for behavioral performance in standard conditions—rodent spontaneous activity in an open field during repeated testing and after treatment with drugs or brain lesions
Source: Gigascience. 2022 Oct 20;11:giac092. doi: 10.1093/gigascience/giac092 (PMC9581716; doi:10.1093/gigascience/giac092)
Supplement: giac092_Reviewer_2_Report_Revision_1 — Inbar Saraf-Sinik -- 8/8/2022 Reviewed [file giac092_reviewer_2_report_revision_1.pdf]

**Reviewer Report**

**Title: A Virtual Library for Behavioral Performance in Standard Conditions â€“ Rodent Spontaneous Activity in an Open Field during Repeated Testing and after Treatment with Drugs or Brain Lesions**

**Version: Revision 1** **Date:** 8/8/2022

**Reviewer name: Inbar Saraf-Sinik**

**Reviewer Comments to Author:**

In the original version, the authors presented their annotation schema, which was commendably clear and well organized despite including very detailed information and describing a large volume of data. In the revised version, the authors complemented this achievement by adding and elaborating on the reuse potential and limitations of their data. Considering the various conditions and treatments implemented in different experiments they included in the database, they explicitly distinguished between the overall size of the database and the effective size and constructed a simple to use tool for potential users to select the relevant data from the entire dataset. With the editors, the authors also decided to provide links to data that are already available in other repositories instead of mirroring the data in the GigaScience database. While the annotation schema and the effective size of probable datasets contribute to the reuse potential of the data, the low video resolution and thus the compatibility of the data with potential reuse the authors suggest, remains unknown, also in the revised version. Demonstrating the feasibility of utilizing these data, with any of the cited machine learning tools, to extract additional features and posture reconstruction should be completed to support the authors' claim regarding such reuse potential.
Reuse potential and Limitations - The reuse potential and complimentary reuse limitations sections are significant additions that the authors chose to introduce. In fact, I think such sections should be mandatory in manuscripts of this type. As a user, it is unfortunate to spend time and effort working with a database, only to learn that it is incompatible with the research question. An honest estimation of a database's reuse potential and limitations contributes to the paper's integrity, demonstrates respect for the time and work of potential users, and, in general, promotes good science. I commend the Authors' choice to introduce and elaborate on them in the revised version.
Database overall and effective size- I am also content with the authors' choice to explicitly distinguish between the overall- and the effective- size of the database. The added paragraph clearly explains how to filter and select the relevant data using the Excel file. I find it easy and efficient to use when selecting the relevant data that potential users require. Including data links further facilitates data accessibility.
Video resolution- The authors have demonstrated that video resolution is well adequate for measuring locomotion trajectories and derivatives. In the reuse potential section, the first example the authors give is: "the library could be used to train neural networks to track body parts and extract postural kinematics;". Throughout the manuscript, they lead towards the significant potential of raw videos, from which one can extract features additional to those originally intended, using current machine learning tools. They do not demonstrate that such appealing projections are even possible using their videos. Which body parts can be reliably tracked by any of the cited tools? To what extent can it be used for

posture reconstruction and kinematics measurements? To what extent do the suggested video-enhancing and upscaling tools facilitate the tracking performance (e.g., increase the range of trackable body parts? contribute to a more elaborated reconstruction of postures? increase SNR and tracking quality?). Does the video resolution support all these?
In response to my concern about the low video resolution, the authors referred to another work that used an entire 352x240 px. ROI to record head and whiskers. Clearly, this example is very different than recording a rat running in a 160x160 cm arena. It is simply irrelevant to whether the video resolution is compatible with the potential reuse the authors suggested. Indeed, as the authors replied, data compatibility is an empirical question. And one that can be easily answered. I realize that the authors cannot be expected to demonstrate the compatibility of their data with every potential data-analysis tool or resolution; however, their choices of citations and examples for reuse follow that, at this level at least, the authors should address this empirical question and provide a concrete estimation. Because such an examination is so easily achievable (for example, training with DeepLabCut, which is open source and beautifully documented), I do not understand the dismissive statement that data compatibility with these products is an empirical question. Advertising a potential involves the responsibility of demonstrating its feasibility.
Behavioral repertoire- The second paragraph in the introduction explains: "Video records, by capturing the entire richness of behavior, have potential utility well beyond their immediate value in a particular experiment. They could be reused as inputs for new experiments to test different hypotheses. They can be reused to analyze aspects of behavior not of interest in the particular experiment. Importantly, they could serve as data for new types of analysis of behavior, not yet available." This paragraph inspired me to consider the behavioral richness in the dataset and led to comment iv in my remarks on the previous version of the manuscript, dealing with the convergence and reduction of behavioral repertoire due to repeated measurements in the same environment. It is true that saline-injected animals should not exhibit OCD-like behaviors, which is the reason they make a good control for the OCD-model groups (brain and pharmacological treatments). Still, repeated exposure to the same stimuli considerably reduces the suggested richness of behavior and any utility that is beyond the original context. With that, the authors cleverly chose examples in the reuse potential section that either relate to the OCD field ("to identify and measure ritual behavior of relevance to OCD …; or to classify effects of different drugs on behavioral activity."), or specifically address the longitudinal questions ("circadian, seasonal or annual rhythms…quantify across trials the range of possible responses, … and across time the change with repeated testing."). Their choice of examples indicates a full understanding of the content of comment iv, and I accept their implicit approach to this issue.
Novelty- I also accept the authors' reply regarding the novelty comment. I believe that their annotation schema clearly details the properties of the database and is elegantly constructed. With the supplements to the reuse potential section, I think the authors did a good job achieving their goals. Furthermore, I support the decision to avoid loading their data to GigaDB, creating unnecessary, and expensive mirroring, but suffice with providing links to the data available in other repositories.
To summarize, I find the authors' replies and revisions satisfactory, with the exception of addressing the limitations posed on the reuse potential due to low video resolution (compared with the typical currently available video cameras). The unique value of this manuscript is the reuse potential of the data, which is a function of size, the annotation schema, and the data quality and compatibility. The

authors achieved the two first points with the straightforward distinction of effective size, the addition of a simple selection tool, and the impressive annotation schema. Regarding data quality and compatibility, I expect the authors to support their claim for the potential reuse with machine learning tools and extraction of additional features, providing concrete evidence for the feasibility of such analysis.

**Level of Interest**

Please indicate how interesting you found the manuscript: Choose an item.

**Quality of Written English**

Please indicate the quality of language in the manuscript: Choose an item.

**Declaration of Competing Interests**

Please complete a declaration of competing interests, considering the following questions:

- Have you in the past five years received reimbursements, fees, funding, or salary from an organisation that may in any way gain or lose financially from the publication of this manuscript, either now or in the future?
- Do you hold any stocks or shares in an organisation that may in any way gain or lose financially from the publication of this manuscript, either now or in the future?
- Do you hold or are you currently applying for any patents relating to the content of the manuscript?
- Have you received reimbursements, fees, funding, or salary from an organization that holds or has applied for patents relating to the content of the manuscript?
- Do you have any other financial competing interests?
- Do you have any non-financial competing interests in relation to this paper?

If you can answer no to all of the above, write 'I declare that I have no competing interests' below. If your reply is yes to any, please give details below.

'I declare that I have no competing interests

I agree to the open peer review policy of the journal. I understand that my name will be included on my report to the authors and, if the manuscript is accepted for publication, my named report including any attachments I upload will be posted on the website along with the authors' responses. I agree for my report to be made available under an Open Access Creative Commons CC-BY license (http://creativecommons.org/licenses/by/4.0/). I understand that any comments which I do not wish to

be included in my named report can be included as confidential comments to the editors, which will not be published.

Choose an item.

To further support our reviewers, we have joined with Publons, where you can gain additional credit to further highlight your hard work (see: https://publons.com/journal/530/gigascience). On publication of this paper, your review will be automatically added to Publons, you can then choose whether or not to claim your Publons credit. I understand this statement.

Yes Choose an item.
